# Cellulose-enabled Hydrovoltaic Energy Generation: from Molecular and Materials Design to Device Integration

**DOI:** 10.1007/s40820-026-02243-3

**Published:** 2026-06-22

**Authors:** EunAe Shin, Guangtao Zan, Kaiying Zhao, Shengyou Li, Gwanho Kim, Minji Kwon, HoYeon Kim, Jin Kie Shim, Cheolmin Park

**Affiliations:** 1 Department of Materials Science and Engineering, Yonsei University, Seoul, 03722 Republic of Korea; 2 Korea Packaging Center, Korea Institute of Industrial Technology, Bucheon, 14449 Republic of Korea

**Keywords:** Cellulose, Hydrovoltaic technology, Sustainable energy harvesting, Self-powered sensor

## Abstract

Hydrovoltaic energy generation (HEG) offers a sustainable route for converting water–solid interactions into electricity; however, guidance tailored to cellulose-based systems remains fragmented. This review provides a comprehensive overview of cellulose-based HEG across the four principal device classes—moisture, evaporation, osmotic, and droplet-induced electricity generators. Further, the review clarifies the role of the structural hierarchy of cellulose, its surface chemistry, and its hydration behavior in electrohydrodynamic transport and device-level performance. Beyond summarizing the prior work, we present system-level benchmarking through a consolidated performance table. The table explicitly links the material composition and device architecture with power output, robustness, and representative application scenarios such as power sources, self-powered sensors, and environmental monitoring, thereby offering actionable design guidelines. We further systematically discuss the chemical principles underlying cellulose-enabled HEG, including interfacial charge regulation, electric double-layer formation and overlap, and Donnan and ion-exchange effects, establishing a coherent theoretical basis for optimizing the voltage, current density, power density, and long-term stability. This mechanism-guided perspective connects the design of cellulose materials to the functional performances of moisture, evaporation, osmotic, and droplet systems. Additionally, it outlines practical directions for the sustainable sourcing, standardized testing, and scalable fabrication of efficient and environmentally friendly hydrovoltaic technologies.

## Hightlights

- This review systematically categorizes emerging cellulose-enabled hydrovoltaic energy generators into four types based on their working mechanisms and synthesizes their research progress.
- Molecular and microstructure–property–performance relationships governing hydration and ion transport are elucidated to provide engineering strategies that leverage cellulose’s intrinsic surface molecular chemistry and structural tunability.
- Emerging applications in user-interactive electronics based on cellulose-enabled hydrovoltaic energy are comprehensively reviewed across power sources and self-powered sensors for biological and environmental activities.

## Graphical abstract

**Figure:**
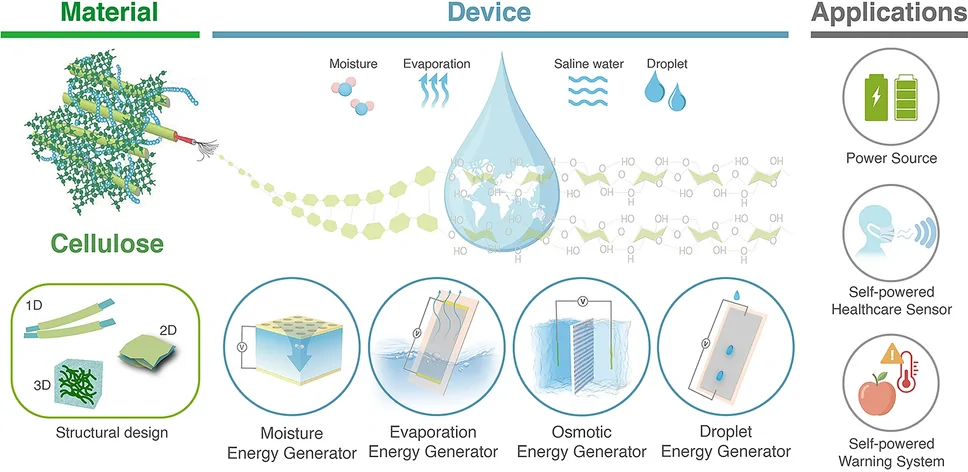

## Introduction

Green energy-harvesting technologies that capture and convert energy from natural sources offer sustainable alternatives to conventional fossil-fuel- and nuclear-based power generation. Several approaches have been developed depending on the energy source, including solar cells that utilize sunlight, thermoelectric generators driven by temperature gradients, piezoelectric and triboelectric devices that convert mechanical energy, and hydrovoltaic generators that harvest energy from water [1–14]. Among these, hydrovoltaic energy generation (HEG) is attracting increasing attention owing to several distinctive advantages. Unlike solar or thermally driven technologies that depend strongly on illumination or heat availability, HEG systems can operate across diverse times and locations [1, 15–20]. Furthermore, HEG devices can generate electricity with minimal direct carbon emissions, positioning them as a promising class of low-carbon energy systems [19, 21–28]. Therefore, hydrovoltaic technologies offer potential routes to address environmental pollution and energy scarcity simultaneously.

**Fig. 1:**
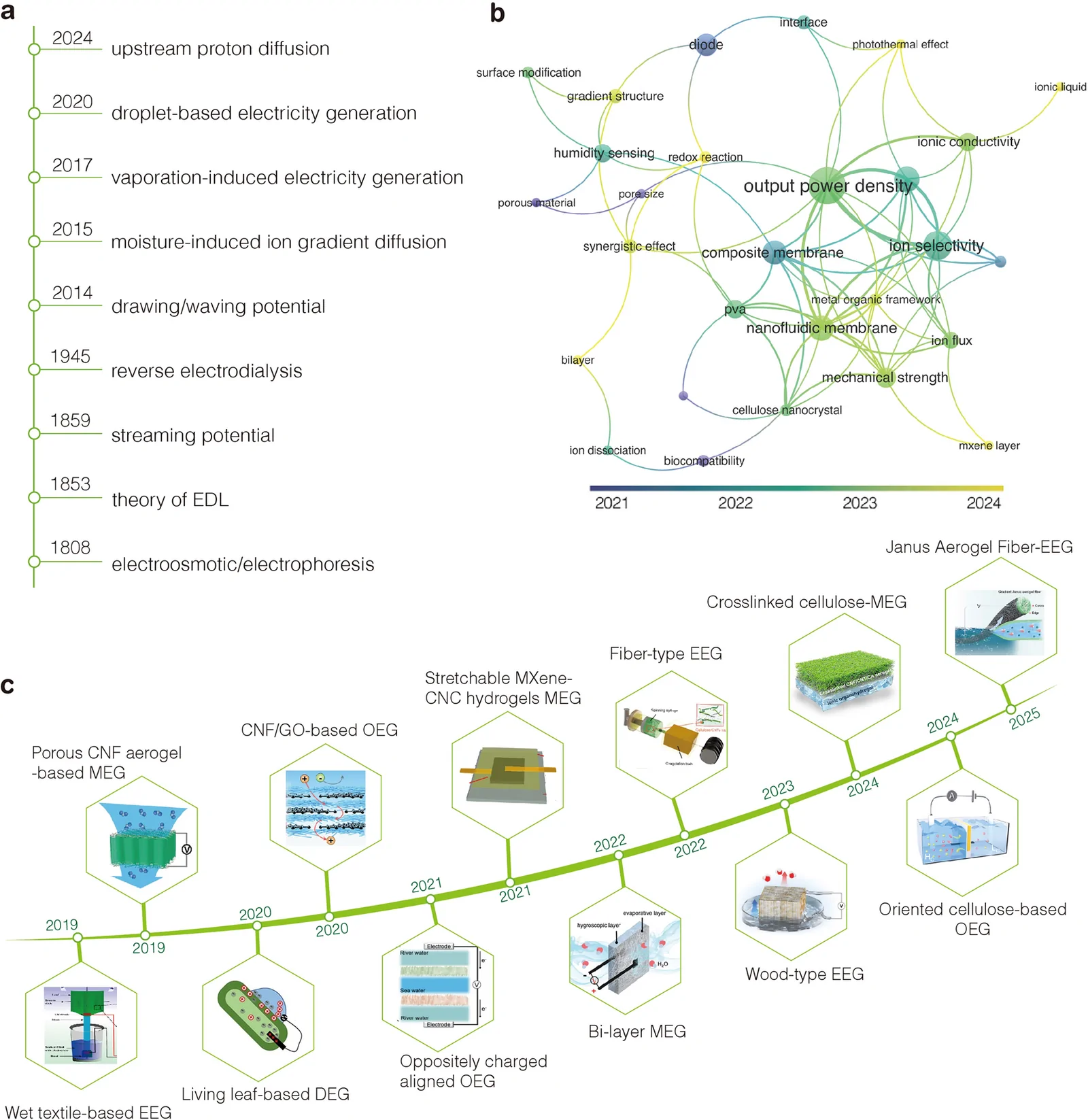
Overview of cellulose-enabled HEGs. **a** Evolution of key theories related to HEGs. **b** VOSviewer network map of keywords associated with cellulose-enabled HEGs. **c** Development timeline of cellulose-enabled HEGs. Reproduced with permission [31]. Copyright 2019, American Chemical Society. Reproduced with permission [32]. Copyright 2019, Wiley–VCH. Reproduced with permission [33]. Copyright 2019, American Chemical Society. Reproduced with permission [34]. Copyright 2020, Royal Society of Chemistry. Reproduced with permission [35]. Copyright 2021, Elsevier. Reproduced with permission [36]. Copyright 2021, Elsevier. Reproduced with permission [37]. Copyright 2022, Springer Nature. Reproduced with permission [38]. Copyright 2022, Wiley–VCH. Reproduced with permission [39]. Copyright 2023, Wiley–VCH. Reproduced with permission [40]. Copyright 2024, Royal Society of Chemistry. Reproduced with permission [41]. Copyright 2024, American Chemical Society. Reproduced with permission [42]. Copyright 2025 Wiley–VCH

Water covers more than 70% of the Earth’s surface and is among the most abundant and renewable natural resources. Beyond its sheer abundance, it is present in diverse forms, including atmospheric moisture (humidity and vapor), freshwater (lakes and rivers), seawater, rainfall, and biofluids such as sweat. This ubiquity and versatility make water an attractive medium for sustainable energy harvesting. HEG systems are designed to directly convert interactions between functional materials and water into usable electricity [29, 30]. Although the term “hydrovoltaic energy generation” has only gained prominence in recent years, its theoretical and experimental foundations extend back more than two centuries (Fig. 1a). In 1808, the discovery of electro-osmosis and electrophoresis laid the groundwork for understanding interfacial ion transport. The subsequent development of the electrical double-layer (EDL) theory further elucidated the charge separation and potential formation mechanisms, as well as structure–function relationships at the water–material interface. The streaming potential—defined as the voltage generated when a liquid flows along a charged solid surface and entrains mobile ions in an EDL—was first reported in 1859 [15] and is widely considered an early manifestation of HEG. Reverse electrodialysis, which was introduced in 1945, provided a theoretical basis for harvesting osmotic energy. Thereafter, the progress on HEG was gradual until the twenty-first century, when advances in nanotechnology accelerated discovery and device development, including the drawing and waving potentials, moisture-induced gradient diffusion, and evaporation-induced electricity generation [6]. On the basis of the interaction mechanisms between water (in its different forms) and functional materials, HEG devices are now commonly categorized into four representative classes: moisture energy generators (MEGs), evaporation energy generators (EEGs), osmotic energy generators (OEGs), and droplet energy generators (DEGs). Each class relies on distinct physicochemical processes to extract electrical energy from water, underpinning a versatile and rapidly expanding field of sustainable energy technologies.

In HEG systems, the active material is the central component and largely dictates the efficiency and long-term stability of electricity generation [43, 44]. Accordingly, several materials have been investigated for HEG applications, including inorganic materials (e.g., carbon-based structures, metal oxides, SiO₂, and MXenes) [45–48], natural materials (e.g., cellulose, alginate, silk fibroin, chitosan, gelatin) [49–51], synthetic polymers (e.g., poly(styrene sulfonic acid) (PSSA), and poly(3,4-ethylenedioxythiophene):polystyrene sulfonate (PEDOT:PSS)) [52–54], and organic–inorganic hybrids such as metal–organic frameworks (MOFs) [55–57]. Among these, cellulose-based materials have emerged as particularly attractive candidates because of their natural abundance, strong hydrophilicity, tunable structure, processability, robust mechanical properties, biocompatibility, biodegradability, and cost-effectiveness. Moreover, cellulose is inherently renewable, nontoxic, and biodegradable, aligning with sustainability and carbon-neutrality objectives. Conversely, many inorganic and hybrid materials, including nanocarbons and MOFs, are not readily biodegradable and may present environmental concerns when deployed at scale [58, 59]. Likewise, many synthetic polymers face end-of-life limitations because they are typically incinerated (releasing greenhouse gases) or landfilled, contributing to long-term plastic accumulation and the environmental burden.

Cellulose is the most abundant natural polymer on Earth and has long-standing industrial relevance [60–63]. Its renewability, biodegradability, mechanical robustness, and chemical tunability have enabled its widespread use across diverse sectors, including textiles (rayon and lyocell), paper and packaging, pharmaceuticals (tablet binders and drug-delivery matrices), food (stabilizers and thickeners), cosmetics, filtration membranes, biomedical scaffolds, and flexible electronics [64–67]. Market analysts estimate that the global cellulose market reached approximately USD 44 billion by 2023, with continued growth expected through 2028. Given this extensive industrial footprint, identifying next-generation functionalities of cellulose beyond traditional applications is both scientifically compelling and strategically important. Cellulose-based HEGs represent a promising direction for value-added utilization, leveraging the intrinsic hydrophilicity, processability, and sustainability of cellulose. Bibliometric mapping using VOSviewer (Fig. 1b) further depicts the diverse and deep research on cellulose-enabled HEG, which spans fundamental material synthesis, composite design, structural modulation, and mechanistic studies. Furthermore, key milestones in the development timeline of cellulose-based HEG devices (Fig. 1c) indicate a dual trajectory: advances in material chemistry alongside the emergence of new HEG mechanisms and continued progress in device dimensionality and architectural design. Together, these trends underscore the rapid, multidisciplinary evolution of the field, which is discussed in detail in Section 3 of this review.

**Fig. 2:**
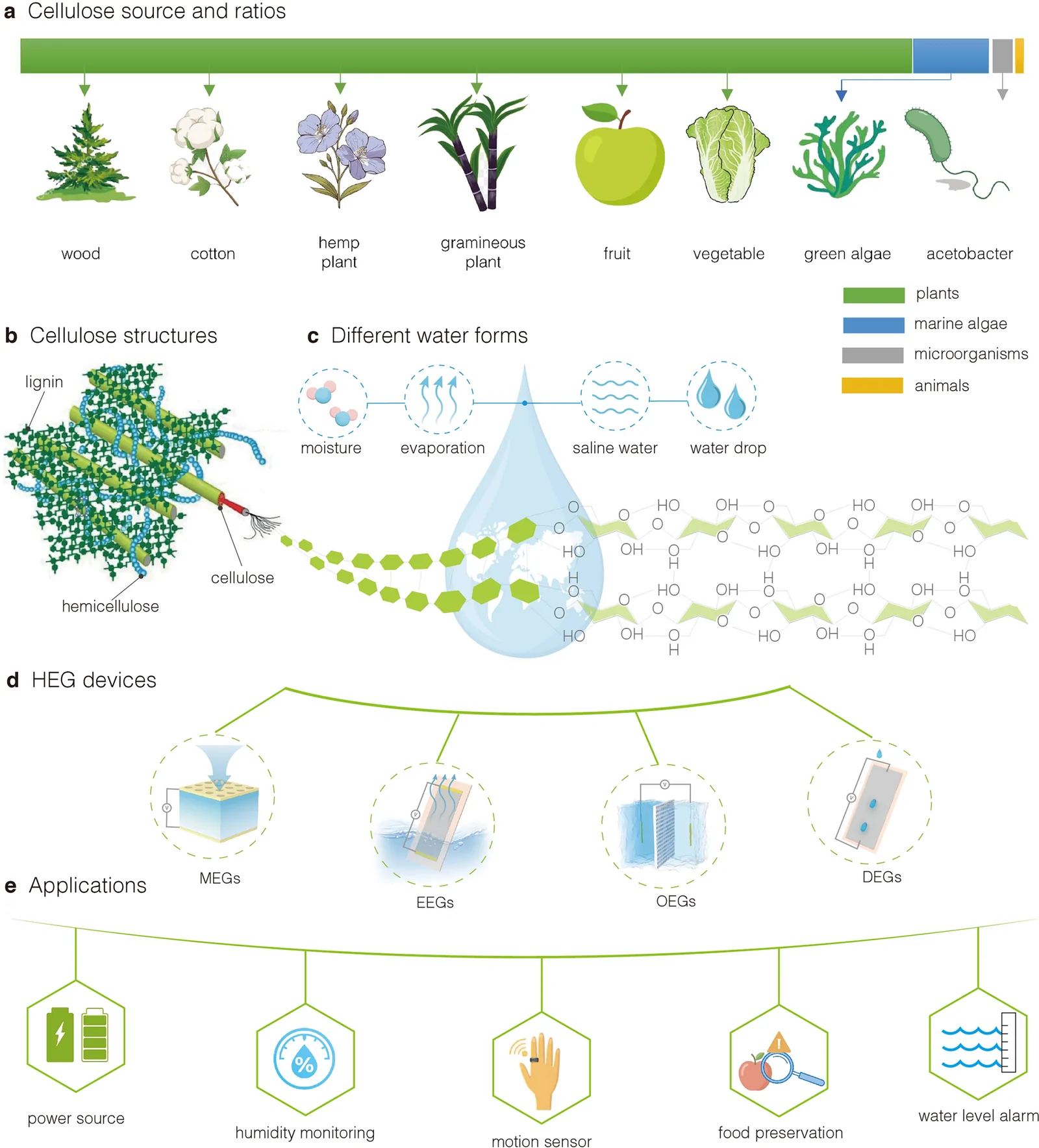
Overview of the structure of this review. **a** Sources and relative proportions of cellulose materials. **b**–**d** Cellulose structures and their interactions with different forms of water, leading to four types of HEGs. **e** Application scenarios for cellulose-enabled HEGs

Despite increasing interest in cellulose-based materials for HEG, a comprehensive and in-depth review of this rapidly evolving field is lacking. As HEG research continues to expand and the area emerges as a frontier in sustainable energy technologies, a timely and systematic review of the progress is needed. Such an overview can clarify the current landscape and help guide the development of practical and scalable cellulose-based HEG systems. Here, we summarize the operating principles of four major HEG categories: MEGs, EEGs, OEGs, and DEGs. We then describe the sources, structural characteristics, fabrication and processing methods, and key physicochemical properties of cellulose materials (Fig. 2a, b). Subsequently, we systematically review advances in cellulose-based HEG across these four device types, emphasizing how interactions between cellulose and different forms of water enable electricity generation (Fig. 2c, d). We further connect the performance attributes to realistic application scenarios, highlighting recent progress in deployment and system integration (Fig. 2e). Finally, we provide a concise assessment of the current status of the field and propose future research directions to accelerate the translation of cellulose-based HEG technologies from laboratory concepts to real-world energy solutions.

## Overview of Cellulose

### Structure of Cellulose and its Derivatives

Cellulose is a linear homopolysaccharide composed of β-(1 → 4)-linked D-glucose units and is the most abundant biopolymer in nature. Each glucose monomer contains three hydroxyl groups that enable extensive intra- and intermolecular hydrogen bonding. These interactions drive the self-assembly of cellulose chains into elementary fibrils (approximately 3–5 nm), which further aggregate into microfibrils (approximately 10–30 nm) and ultimately form macroscopic fibers in plant cell walls or bacterial cellulose (BC) pellicles [68, 69]. This hierarchical organization includes crystalline regions with highly ordered chain alignment and amorphous regions with less-ordered structures. The relative abundance of these domains governs key physicochemical properties of cellulose, including its mechanical strength, swelling behavior, and chemical reactivity [70, 71]. Crucially, the hierarchical porosity originating from these nanoscale interstices between microfibrils provides an ideal structural framework for capillary-driven water transport and nanoconfined fluidic phenomena, which are fundamental to the operation of hydrovoltaic devices.

The structural complexity and polymorphic diversity of cellulose underpin a broad range of functional material properties. The abundance of hydroxyl groups confers strong hydrophilicity and hygroscopicity, enabling efficient water absorption and retention [72–74]. These characteristics are particularly advantageous for HEG systems, wherein continuous interaction with water is required for sustained output. Moreover, the high tensile strength associated with crystalline microfibril domains supports mechanically robust yet flexible devices [70, 71]. Cellulose is biocompatible, consistent with its natural origin, which favors biointegrated applications. Also, its thermal stability and flexibility facilitate operation across diverse environmental conditions and integration into wearable, textile-based, or irregularly shaped platforms. Furthermore, the dense distribution of surface hydroxyl groups facilitates the formation of a robust EDL within the hydrated cellulose matrix, enabling efficient charge separation and ion-selective transport, which govern the voltage-generating mechanisms in hydrovoltaic systems.

Especially, the biodegradability of cellulose provides a critical material-level justification for the sustainability of HEG systems, aligning with the broader goal of eco-friendly energy conversion. This biodegradation primarily proceeds via enzymatic hydrolysis, where cellulase enzymes produced by bacteria and fungi cleave the β-(1,4)-glycosidic linkages [75, 76]. This process breaks down long polymer chains into shorter oligosaccharides and eventually glucose, which is then metabolized by microorganisms. The rate of degradation is highly dependent on environmental conditions, such as humidity and temperature, and the diversity of microbial populations. Specifically, the presence of cellulolytic fungi (e.g., *Trichoderma* and *Aspergillus*) and bacteria (e.g., *Cellulomonas* and *Clostridium*) plays a decisive role, as these species secrete distinct cellulase systems that synergistically breakdown the crystalline and amorphous regions of the cellulose fibers [75]. While pure cellulose typically decomposes within several weeks to a few months in soil or composting environments, the exact timeline can be modulated by the degree of crystallinity and chemical modifications. The biodegradability of cellulose has been extensively exploited in fields such as sustainable packaging, biomedical scaffolds, and transient electronics, in addition to HEG systems. However, in the specific context of hydrovoltaics, quantitative empirical data regarding degradation behavior—particularly the balance between operational stability and end-of-life decomposability—remains relatively limited. Further systematic studies are required to substantiate the long-term environmental benefits of cellulose-based HEGs compared to synthetic alternatives.

**Fig. 3:**
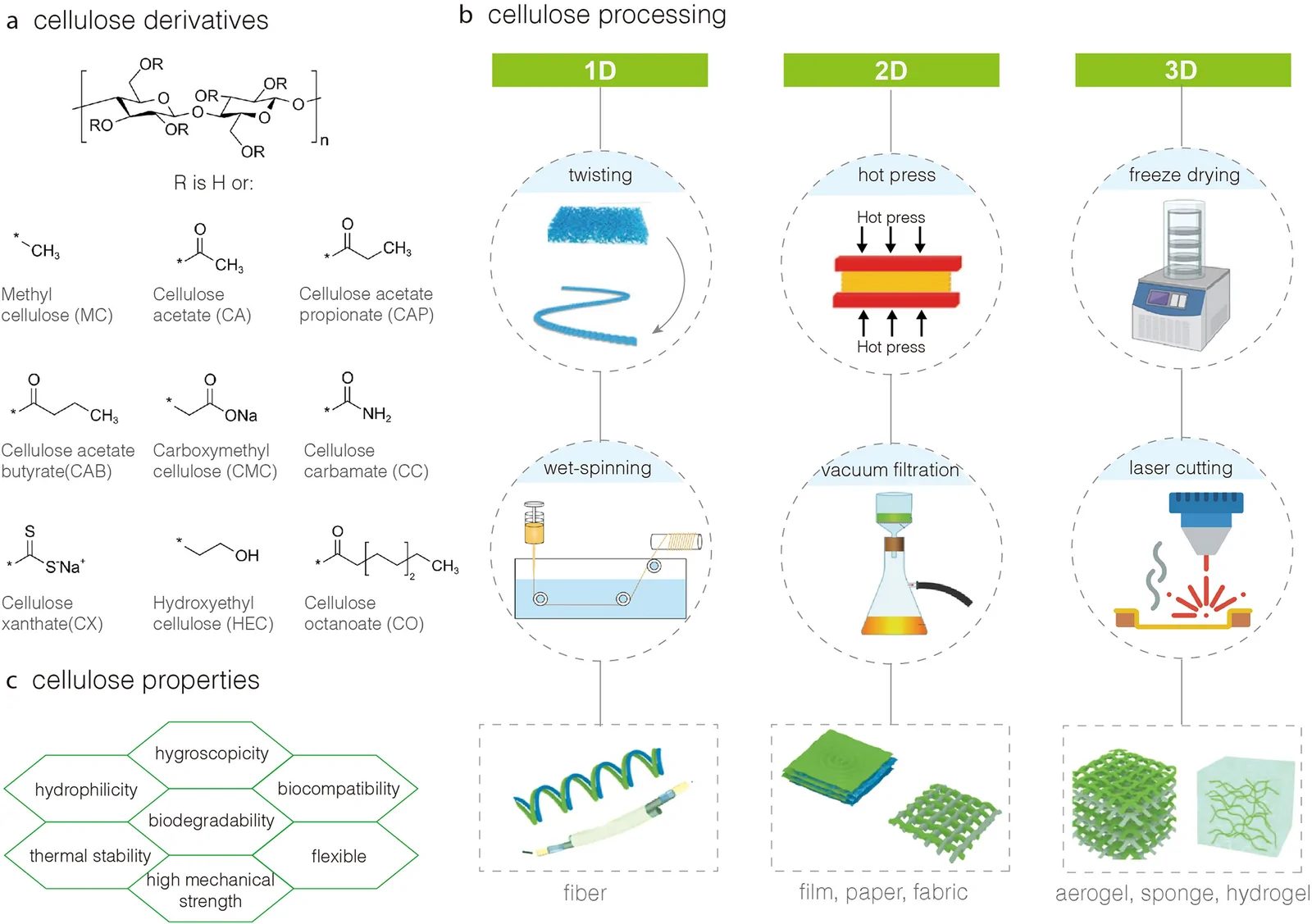
Types, processing methods, and properties of cellulose-based materials. **a** Cellulose derivatives. **b** Cellulose processing, and **c** Cellulose properties. Reproduced with permission [78]. Copyright 2023, American Chemical Society

The structural versatility of cellulose is fundamentally rooted in its molecular chemistry (Fig. 3a). This stems from the reactivity of the three hydroxyl (–OH) groups present on each anhydroglucose unit (AGU). The AGU serves as the basic repeating monomer of the cellulose chain and is essentially a single dehydrated glucose molecule. These intrinsic functional groups are strategically positioned at the C2, C3, and C6 positions, each exhibiting distinct chemical reactivity. The primary hydroxyl group at the C6 position typically shows higher reactivity due to lower steric hindrance, making it a preferred site for selective modifications, such as TEMPO-mediated oxidation which converts –OH into negatively charged carboxylate (–COO⁻) groups. In contrast, the secondary hydroxyl groups at C2 and C3 participate in dense intra- and intermolecular hydrogen-bonding networks, which govern the crystallinity and internal cohesion of the polymer. Beyond its native form, cellulose can be chemically modified at hydroxyl sites to generate derivatives with tailored functionalities. Such modifications, which are most commonly introduced via etherification or esterification, enable the tuning of the solubility, hydrophobicity, thermal behavior, and interfacial characteristics. Accordingly, numerous cellulose derivatives have been developed to adapt the physicochemical properties of cellulose to specific applications [76, 77]. Among these, carboxymethyl cellulose (CMC), produced by the etherification of hydroxyl groups with chloroacetic acid, introduces dense carboxylate functionalities. These groups increase the surface charge density, enhancing ionic conductivity and interfacial charge transport in moisture- and osmotic-driven HEG devices. Conversely, cellulose acetate (CA), synthesized through the esterification of –OH groups with acetic anhydride, offers controlled hydrophilicity and mechanical stability, enabling sustained water diffusion under evaporative conditions. Methyl cellulose (MC), characterized by the partial replacement of –OH with methoxy groups, exhibits reversible thermoresponsive behavior, supporting adaptive power generation. Recent studies also indicate that integrating these molecularly engineered derivatives with ionic liquids (ILs) or conductive nanofillers can synergistically enhance EDL formation and overall ion mobility, further advancing the performance and durability of cellulose-based HEG platforms.

Despite these advantages, the molecular architecture of cellulose—defined by an extensive network of intra- and intermolecular hydrogen bonds—presents inherent challenges for long-term operational stability. In moisture-rich or fully immersed environments, water molecules act as aggressive competitors for the hydroxyl (–OH) bonding sites, particularly within the less-ordered amorphous domains [69, 78]. This water–solid interaction triggers a ‘plasticization’ effect, where the penetration of water molecules disrupts the inter-chain cohesion and induces significant structural relaxation, leading to macroscopic swelling. Mechanically, this structural expansion compromises the dimensional stability of the framework, often resulting in the softening or irreversible deformation of the device. Consequently, the gradual decay in surface charge density and the loss of nanoconfinement lead to a sustained reduction in both voltage and current output.

Overall, the synergy between the inherent physicochemical attributes of native cellulose and structural versatility afforded by its derivatives establishes a highly tunable platform for modulating the surface charge (zeta potential, ζ), hierarchical porosity, hydration kinetics, and ion-selective transport—parameters that are fundamentally central to optimizing the performance of cellulose-based HEG systems.

### Processing and Properties of Cellulose

A defining attribute of cellulose is the synergy between its surface molecular chemistry and structural versatility, which enables the fabrication of multiple dimensional architectures, including one-dimensional (1D) fibers, two-dimensional (2D) films, and three-dimensional (3D) aerogels (Fig. 3b). While its physical dimensionality dictates the fluidic transport pathways, the surface chemistry of cellulose—primarily governed by the density and orientation of hydroxyl (–OH) groups—serves as the primary driver for interfacial charge separation. These active sites allow for precise molecular engineering, such as the introduction of charged functional groups (e.g., carboxyl, amine, or phosphate groups) that tune the zeta potential and ion-exchange capacity of the resulting framework. Consequently, the processing of cellulose is not merely a structural shaping but a systematic interfacial engineering that optimizes the interaction between water molecules, mobile ions, and the solid surface (Fig. 3c).

#### 1D Fibers, Cellulose Nanofibers, and Cellulose Nanocrystals

The linear backbone and strong intermolecular hydrogen bonding of cellulose facilitate the formation of fibrous and nanofibrillar architectures. In addition to conventional spinning and mechanical fibrillation, emerging approaches such as dry-jet wet spinning and field-assisted alignment can produce highly ordered, directionally aligned fibrillar networks [76, 79]. These aligned cellulose fibers exhibit anisotropic charge conduction and guided ion transport, thereby increasing the current and voltage by maximizing the streaming potentials along the fiber axis.

Electrospun or field-aligned nanocellulose fibers inherently contain interconnected capillary structures that promote rapid water transport via capillary action, sustaining a continuous evaporation-driven water flux. This persistent flow maintains a stable electrochemical potential gradient and enhances evaporation-induced electricity generation. Recent fabrication strategies further incorporate multiscale control of the hierarchical porosity—within individual fibers and across fiber bundles—to optimize the water transport and charge separation. Such architectures accelerate wicking, maintain sustained moisture flow along the fiber axis, and increase both the stability and power density of the hydrovoltaic output.

At the nanoscale, cellulose nanofibers (CNFs) and cellulose nanocrystals (CNCs) offer a tunable surface chemistry and structural regularity that can promote ion diffusion and streaming-potential generation. CNFs form entangled, flexible networks that support continuous water pathways, whereas CNCs introduce rigid crystalline domains that favor stable charge separation. When the effective diameter of these nanochannels approaches the Debye length (λ_D)_ of the electrolyte, EDLs at the cellulose–water interfaces overlap. This EDL overlap breaks charge-transport symmetry and promotes preferential counterion migration, thereby amplifying the streaming potential induced by water flow. The resulting asymmetric ion transport enhances both open-circuit voltage and ion-separation efficiency, two key metrics governing hydrovoltaic conversion.

In addition to mechanical alignment, CNCs produced via sulfuric acid hydrolysis inherently carry negatively charged sulfate half-ester groups, whereas TEMPO-oxidized CNFs introduce carboxylate functionalities. These surface charge engineering of these 1D nanostructures provide strong electrostatic repulsion, which prevents fiber aggregation and ensures a uniform porous network during processing. Beyond structural stability, the elevated zeta potential creates a charge-dense environment within the nanofibrillar matrix. Such molecular-level tuning facilitates selective counterion migration through nanoconfined channels, a critical requirement for maximizing streaming potential and overall hydrovoltaic efficiency.

By integrating hierarchical porosity engineering with control over the structural alignment, 1D cellulose-based architectures can evolve from passive supports into directionally active energy transducers that couple the wetting dynamics with ion streaming. Collectively, these advances enable the simultaneous optimization of the voltage, current, response stability, and cycling durability in hydrovoltaic systems.

#### 2D Papers, Films, and Membranes

Cellulose can be processed into 2D formats such as papers, films, and membranes, in which extensive hydrogen bonding among fibrils yields mechanically robust yet flexible planar networks. Recent processing strategies emphasize the hierarchical control of the porosity within these 2D structures, enabling concurrent regulation of water transport, ion migration, and charge separation—key determinants of hydrovoltaic energy generation. In contrast to 1D fibers that primarily rely on axial flow, 2D cellulose assemblies can exploit lateral or through-plane gradients, providing greater design flexibility for planar and wearable applications.

A major advance in this area is the generation of multiscale, anisotropic porosity using approaches such as directional drying, phase-separation-assisted casting, and templated filtration. These methods introduce nanochannels within the cellulose matrix or between stacked layers, creating confined pathways for ion transport. When the characteristic channel size or interlayer spacing approaches the λ_D_ of the electrolyte, EDLs overlap at the cellulose–water interfaces. This nanoconfinement breaks the charge-transport symmetry and enhances selective counterion migration, thereby significantly improving open-circuit voltage and ion-separation efficiency under moisture flow or evaporation.

Such geometric optimization is further augmented by tailoring the interfacial chemistry of the cellulose fibrils. By modifying the surface hydroxyl groups with specific ionic or hydrophobic molecules, the interaction between water and the pore walls can be precisely controlled. This chemical tuning regulates the capillary driving force that pulls water into the membrane, which is essential for maintaining a steady flow and maximizing electrical output. Furthermore, introducing functional groups such as carboxyl or phosphate units optimizes the local charge density. This molecular-level regulation of transport kinetics allows 2D cellulose networks to move beyond passive structural confinement, acting as active, ion-selective layers that enhance overall energy-conversion efficiency.

The hierarchically porous 2D cellulose films and membranes act as ion-selective and moisture-responsive energy layers. Their coupled micro- and nanopore architecture supports continuous water diffusion and regulated evaporation, sustaining a stable electrochemical potential gradient during hydrovoltaic operation. Moreover, the tunability of the porosity—from dense nanopaper to open-structured, filter-like membranes—allows precise balancing of mechanical durability, flexibility, and ion mobility.

This structural versatility makes 2D cellulose particularly suitable for paper-based hydrovoltaic devices, membrane-type moisture harvesters, and textile-integrated energy fabrics. Paper-like films provide lightweight and biodegradable substrates for ambient electricity generation; membrane filters enable directional ion transport for a sustained voltage output; and cellulose fabrics leverage the hierarchical porosity to convert natural humidity gradients into electrical energy while maintaining breathability and wearability. Together, these developments highlight how advanced porosity engineering can transform conventional cellulose papers and membranes from passive separators into functional planar energy transducers that combine environmental adaptability with sustainable hydrovoltaic power conversion.

#### 3D Aerogels, Hydrogels, and Scaffolds

Beyond planar architectures, cellulose can be assembled into 3D hierarchical frameworks, including aerogels, hydrogels, and scaffolds [80]. These volumetric networks contain interconnected macro-, meso-, and micropores, providing a high surface area and tunable water-retention capacity. Hierarchical porosity supports sustained water absorption and evaporation over large areas, thereby expanding the active region for charge separation and prolonging the potential difference required for hydrovoltaic operation. Methods such as freeze casting, supercritical drying, and sol–gel templating have been developed to control the pore interconnectivity and directional alignment within 3D cellulose networks.

Recent progress has focused on engineering the interfacial molecular chemistry of 3D cellulose frameworks through hybridization with ionic liquids (ILs) [81, 82]. Beyond disrupting internal hydrogen bonding for uniform processing, ILs serve as molecular templates that define the electrochemical environment at the cellulose–water interface. By interacting with the dense surface hydroxyl groups, ILs facilitate the formation of stable EDLs within the regenerated 3D matrix.

To further stabilize these molecular networks, chemical crosslinking strategies—using agents such as citric acid or epichlorohydrin—are integrated to prevent structural collapse and regulate swelling behavior. These chemical bridges do not only provide mechanical robustness but also introduce additional ionic sites that sustain the EDL throughout the volumetric scaffold. This molecular-level integration ensures high ion mobility and charge-separation efficiency, even in fully saturated states. Furthermore, the ability to tune the IL’s hydrophilicity allows for precise control over water adsorption and ion dissociation kinetics, directly addressing the requirements for stable, high-output hydrovoltaic conversion in diverse 3D environments.

Collectively, these advances demonstrate that 3D cellulose architectures integrate structural hierarchy with interfacial electrochemistry to enable efficient and durable hydrovoltaic energy conversion. The synergy between multiscale porosity, moisture-retentive hydrogen-bond networks, and ion-responsive interfaces sustain continuous water transport and charge separation across the bulk material. Consequently, 3D aerogels and hydrogels deliver increased power output and operational stability while offering design flexibility for scalable, lightweight, and environmentally adaptive hydrovoltaic systems. This integration of architectural design and ionic functionality represents an important step toward sustainable electricity generation from ambient water and humidity.

### Cellulose in HEGs

Cellulose plays multifaceted roles in HEG. First, it functions as an efficient active material for power generation. Its hierarchical porosity and surface charges facilitate ion transport, directly contributing to the electrical output. Second, cellulose acts as a structural substrate or processing aid in composite systems. It integrates seamlessly with other materials such as carbon, metal oxides, and ionic polymers. In these configurations, cellulose serves as a dispersant or template for nanofiber fabrication, ensuring both structural integrity and processability. While other components may dominate the electricity generation in some composites, cellulose remains essential for overall device performance. Finally, cellulose performs auxiliary roles by acting as a hygroscopic or hydrophilic layer. These layers leverage the intrinsic ability of cellulose to manage moisture gradients and water adsorption, supporting the operational environment of other functional components.

## Working Mechanisms of Cellulose-enabled HEGs

Cellulose-based materials are attractive for HEG applications because they combine abundant surface functionality, hierarchical porosity, hydrophilicity, and mechanical flexibility. These properties support multiple, distinct yet interrelated energy-transduction mechanisms, each governed by interfacial interactions between water (liquid, vapor, or droplets) and the charged solid framework. Sections 3.1–3.5 systematically describe the principal pathways for electricity generation in cellulose-based HEG devices: (i) EDL formation, (ii) evaporation-driven ion transport, (iii) moisture-gradient-induced diffusion, (iv) osmotic-pressure-driven ion exchange, and (v) droplet-interface-induced charge displacement. By clarifying these mechanisms, we establish a theoretical basis for optimizing materials and device architectures for high-performance, eco-friendly HEG.

### EDL

**Fig. 4:**
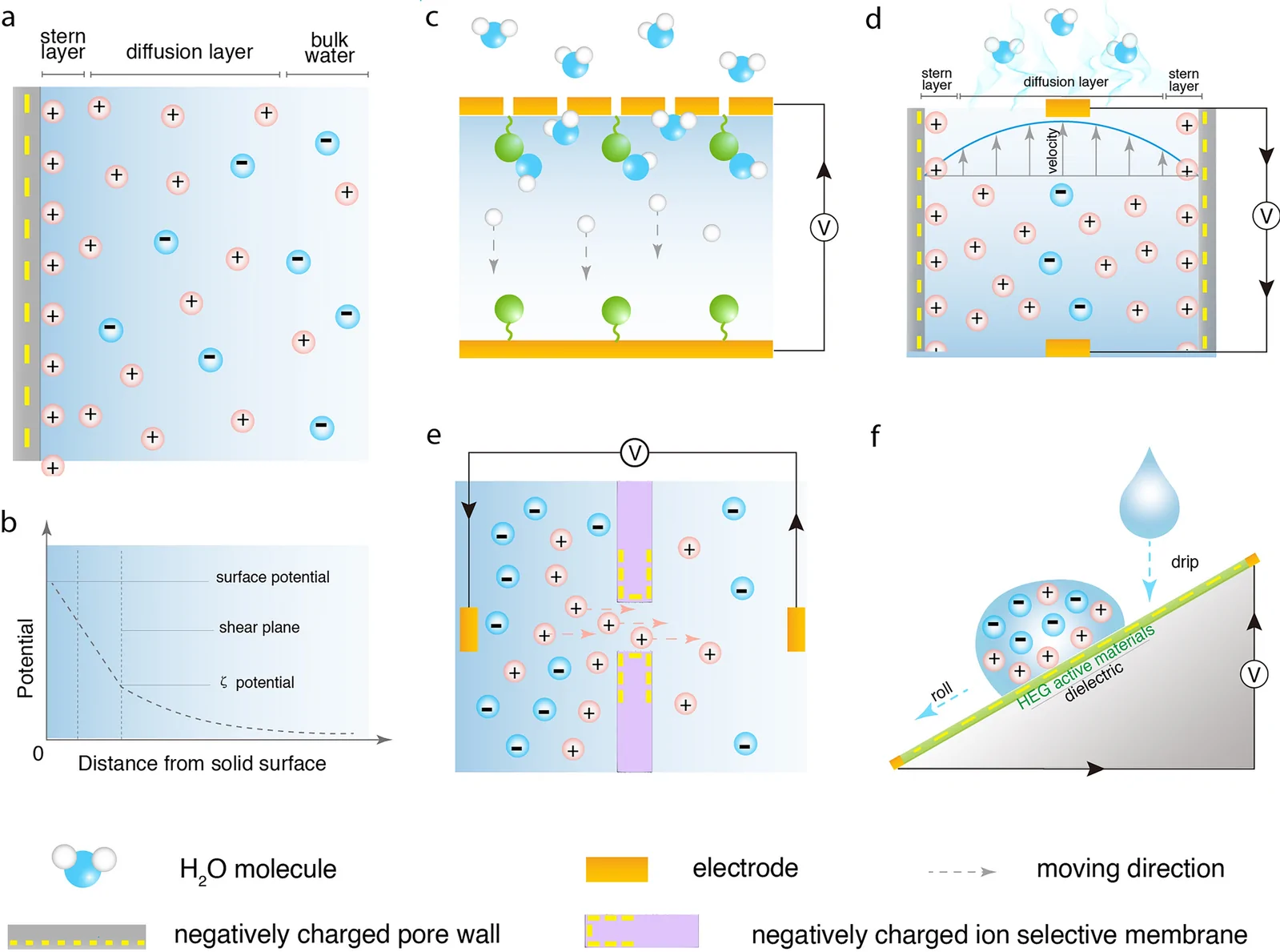
Energy generation mechanisms of different types of HEGs: **a, b** EDL, **c** MEG, **d** EEG, **e** OEG, and **f** DEG

An EDL is a fundamental interfacial structure that forms at the boundary between a charged solid and an adjacent electrolyte and is central to ion–electron coupling in HEG devices. An EDL arises from the spatial separation of opposite charges across the solid–liquid interface, which generates an interfacial potential gradient that can be harnessed for energy conversion. It comprises two regions: (1) a compact, immobile layer of counterions adsorbed on the charged solid surface via electrostatic attraction or specific chemical interactions and (2) a diffuse layer extending into the bulk liquid in which counterions and co-ions are more loosely distributed as electrostatic forces compete with thermal (Brownian) motion (Fig. 4a). Within this diffuse layer, a shear plane separates immobilized from mobile fluid regions; the electrostatic potential at this plane is the ζ (Fig. 4b). This structure is commonly described by the Helmholtz–Gouy–Chapman–Stern model, in which specifically adsorbed ions form a compact Helmholtz layer and the diffuse ionic distribution follows the Poisson–Boltzmann relation. The characteristic thickness of the diffuse region, defined by the λ_D_, determines the range over which the potential extends into the liquid phase of a symmetric monovalent electrolyte:

1 $${\lambda}_{D}=\sqrt{\frac{\varepsilon {k}_{B}T}{2{N}_{A}{e}^{2}I}}$$

for a symmetric monovalent electrolyte, where **ε** is the absolute permittivity of the medium, k_B_ is the Boltzmann constant, T is the absolute temperature, N_A_ is Avogadro’s number, e is the elementary charge of a proton, and I is the ionic strength of the electrolyte. Notably, the Debye length is inversely related to ionic strength; therefore, dilute electrolytes produce thicker diffuse layers and can increase the interfacial selectivity.

Cellulose is rich in hydroxyl (–OH) groups and can also present carboxyl (–COOH) or sulfonic (–SO₃H) functionalities after chemical modification, making it a suitable scaffold for EDL formation in aqueous environments. Upon hydration, these surface groups partially ionize and impart a net surface charge (often negative due to deprotonated hydroxyl or carboxyl groups). This charging promotes the formation of a counterion-enriched Stern layer, which typically contains protons (H⁺) or hydrated metal cations (Na⁺, K⁺), followed by a diffuse layer that compensates the remaining surface charge. The intrinsic hydrophilicity and nanoscale fibrillar architecture of cellulose enhance water uptake and support extensive EDL formation throughout its porous network, making it an effective medium for harvesting interfacial ion–electron coupling effects.

In cellulose-based HEGs, the EDL is not merely a passive interfacial feature but a functional element that enables key transduction processes. (1) Ion-selective transport: the EDL-associated electric field promotes preferential counterion migration through confined nanochannels, enhancing directional charge separation and suppressing recombination losses. (2) Streaming-potential amplification: capillary-driven water flow within cellulose nanopores advects mobile ions from the diffuse layer, generating a streaming current and an associated potential difference proportional to the flow rate and zeta potential. (3) Increased surface charge density: chemical functionalization of cellulose (e.g., via sulfonation or carboxylation) can raise surface charge density, strengthen EDL, and improve device output.

In saline-driven systems, the dominant charge carrier depends on nanochannel surface charge polarity, pH, ionic strength, and ion mobility. For negatively charged cellulose nanochannels, cations are selectively transported. Under acidic or low-ionic-strength conditions, protons (H⁺) can dominate charge transport due to their exceptionally high mobility. In contrast, under neutral or high-salinity conditions, ion transport is typically dominated by conventional cations such as Na⁺ or K⁺ because of their much higher concentration. For positively charged nanochannels, anions become the dominant charge carriers. Proton transport via the Grotthuss mechanism becomes significant when a continuous hydrogen-bond network exists in confined hydrophilic nanochannels or polymer electrolyte networks containing abundant hydrogen-bonding groups. When the nanochannel size increases or the ionic strength becomes higher, electrostatic screening and ion–water interactions progressively disrupt the continuity and lifetime of the hydrogen-bond network. As a result, proton transport via the Grotthuss mechanism becomes less favorable, and ion transport gradually shifts toward conventional vehicular diffusion of hydrated ions. In addition, the dynamic and partially disordered nature of hydrogen-bond networks in swollen cellulose matrices further limits long-range proton hopping, making the relative contribution of Grotthuss transport highly sensitive to crystallinity, chemical modification, and hydration state.

Overall, HEG performance is governed by the interplay between EDL characteristics and the physicochemical properties of cellulose. Accordingly, rational design strategies that tune surface charge, optimize pore architecture, and control electrolyte interactions are essential for advancing cellulose-based HEGs. It should be noted that classical EDL theory assumes rigid and uniformly charged interfaces, which may not fully apply to cellulose-based systems. In hydrated cellulose matrices, swelling, heterogeneous pore structures, and dynamic surface chemistry lead to spatially nonuniform and time-dependent EDL configurations. Therefore, the EDL in such systems is better described as a dynamically evolving and heterogeneous interfacial structure, where local EDL overlap and non-overlap coexist across different length scales.

### Mechanism of MEGs

MEGs harness spontaneous interactions between atmospheric water vapor and hydrophilic materials to induce ion separation and directional migration, thereby generating electrical power (Fig. 4c). In cellulose-based systems, this mechanism is governed by the intrinsic chemical functionality and hierarchical porous architecture of the materials.

Electricity generation in MEGs can be described in three stages: 1) Water adsorption: hygroscopic cellulose fibers readily absorb atmospheric moisture owing to their abundant hydroxyl (–OH) groups and—after chemical modification—carboxyl (–COOH) or sulfonic (–SO₃H) groups, forming a surface hydration layer. 2) Surface ionization and hydrolysis: water–material interactions partially ionize surface functionalities (e.g., –COOH → –COO⁻ + H⁺), producing mobile protons (H⁺) or other cations and leaving immobilized anionic groups anchored to the polymer backbone. 3) Asymmetric ion diffusion: spatial gradients—arising from intrinsic material asymmetry (e.g., nonuniform functional-group distribution) or externally imposed environmental gradients (e.g., humidity differences)—drive directional ion migration, resulting in charge separation and a measurable electric potential.

The chemical and physical characteristics of cellulose make it a particularly suitable platform for MEGs. 1) Functional-group density: the polymer surface provides numerous ionizable sites, enabling substantial generation of mobile ionic species upon moisture uptake. 2) Moisture-responsive architecture: the porous and fibrillar morphology provides a large accessible surface area for water adsorption and establishes continuous pathways for ion diffusion. 3) Gradient engineering: through selective chemical modification or controlled environmental exposure, cellulose can be engineered to exhibit asymmetric distributions of functional groups or hydration levels, thereby strengthening the directional ion flux required for sustained power output.

The efficiency of cellulose-based MEGs is governed by both intrinsic and extrinsic factors. 1) Material composition: hydrophilic polymers with a high density of weakly bound protons (e.g., sulfonated or carboxylated celluloses) typically yield more charge carriers under humid conditions. 2) Environmental conditions: a higher relative humidity (RH) and temperature promote water uptake and surface ionization, thereby increasing the concentration of charge carriers. 3) Ion mobility and selectivity: the pore architecture of the material controls the ion-transport rates, whereas asymmetric channel designs or functionalization patterns reinforce unidirectional diffusion, which is essential for maximizing the voltage output.

Ion migration is driven by chemical potential gradients established by asymmetric moisture uptake or nonuniform functional-group distributions, which together create a built-in electrochemical potential difference across the material. Accordingly, optimization strategies for cellulose-based MEGs include increasing the density of ionizable surface groups via chemical modification, engineering multilayer or gradient-structured materials to enhance the ion-concentration gradients, designing pore networks that facilitate rapid ion transport while maintaining directional control, and precisely controlling environmental exposure (e.g., imposing humidity gradients or applying patterned hydrophobic coatings) to generate robust asymmetric hydration profiles.

In summary, cellulose-based MEGs convert ubiquitous, passive interactions between atmospheric moisture and hydrophilic materials into directed ion transport and electrical energy. The rational optimization of both material properties and device architecture is essential for unlocking their full potential.

### Mechanism of EEGs

EEGs leverage the continuous and spontaneous phase transition of water from a liquid to a vapor to drive ion transport and generate electrical energy (Fig. 4d). When coupled with cellulose-based materials, this mechanism is particularly effective owing to the intrinsic hydrophilicity, high porosity, and capillary-active architecture of the cellulose matrix.

EEG operation is governed by the coupling between capillary water transport and interfacial charge separation. As water evaporates from the exposed regions of a porous, charged material, capillary action continuously replenishes the liquid from the submerged or hydrated regions. This sustained flow entrains mobile ions (primarily counterions within an EDL) through confined nano- or microporous channels, creating a net streaming current and establishing an electric potential across the material. This process is closely related to the classical *streaming-potential effect,* in which pressure-driven flow through a charged porous medium induces ion migration and charge accumulation at the boundaries, generating an electrical signal. However, in EEG systems, the applied pressure differential is effectively replaced by an evaporation-induced capillary gradient, enabling self-sustained operation under ambient conditions. The resulting streaming potential (Vₛ) and streaming current (Iₛ) scale with the ζ potential and driving pressure gradient according to the Helmholtz–Smoluchowski relation:

2 $${V}_{s}=\frac{{\varepsilon}_{0}{\varepsilon}_{r}\zeta \Delta P}{\sigma \eta }$$

3 $${I}_{s}=\frac{{\varepsilon}_{0}{\varepsilon}_{r}\zeta A\Delta P}{\eta l}$$

where ε_0_ is the vacuum permittivity, ε_r_ is the dielectric constant of the solution, ζ is the zeta potential, ΔP is the driving pressure (here replaced by the evaporation-induced capillary pressure), σ is the specific conductivity of the solution, η is the fluid viscosity, A is the cross-sectional area, and l is the flow path length. This framework quantitatively links EDL properties to macroscopic voltage and current generation in EEGs and provides a basis for performance optimization.

The nanofibrillar and mesoporous structure of cellulose offers several advantages for an EEG. (1) Enhanced capillary flow: the dense network of interconnected pores enables rapid water transport from the hydrated reservoir to the evaporation interface, maintaining a stable ion flux. (2) Surface charge-mediated ion selectivity: functional groups on cellulose fibers (e.g., –OH, –COOH, and –SO₃H) impart surface charge, promoting selective counterion enrichment in the flowing liquid and improving charge-separation efficiency. (3) EDL-overlap effects: in sufficiently narrow cellulose channels, the Debye layers from the opposing walls overlap, excluding co-ions and enabling near single–charge-carrier transport, thereby maximizing energy-conversion efficiency.

The EDL-overlap regime typically occurs when the Debye length (λ_D_) becomes comparable to or larger than the effective nanochannel radius (r), i.e., λ_D_ ≈ r. Under such conditions, the electrical double layers from opposite channel walls overlap, leading to ion-selective transport and enhanced streaming potential [43, 44]. For aqueous electrolytes at room temperature, the Debye length can be estimated by: λ_D_ ≈ 0.304/I^0.5^ (nm), where I is the ionic strength in mol L⁻^1^. This relationship suggests that EDL overlap in cellulose nanochannels (typically 2–100 nm) generally occurs in low ionic-strength conditions, typically in the range of ~ 10^–4^ to 10^–2^ M, depending on the nanochannel size and surface charge density. This provides a practical guideline for designing cellulose-based nanochannel systems operating in the EDL-overlap regime.

The performance of cellulose-based EEGs depends on several key parameters. (1) Evaporation rate: Higher ambient temperature, increased airflow, or lower humidity accelerates evaporation, thereby enhancing capillary flow and the associated ionic current. (2) Zeta potential: A higher surface potential strengthens the coupling between water flow and ion migration, thereby increasing the streaming potential. (3) Channel dimensions and pore connectivity: Optimized nanochannel sizes promote EDL overlap without compromising permeability, balancing ion selectivity and flow capacity. (4) Material modification: Chemical treatments (e.g., oxidation and sulfonation) can tailor the cellulose surface chemistry to tune charge density and hydrophilicity, providing a practical route to optimize power output.

Cellulose-based EEGs convert the ubiquitous energy of water evaporation into electrical signals by coordinating capillary water transport, surface charge effects, and nanoscale ion dynamics. Further advances in materials engineering—particularly in pore architecture and surface chemistry—will be central to realizing the full potential of this emerging hydrovoltaic platform.

### Mechanism of OEGs

OEGs harvest free energy released from ion transport driven by salinity gradients, typically between high- and low-concentration electrolyte solutions (Fig. 4e) [83–85]. Because this process is governed by chemical potential differences, it can be effectively implemented using ion-selective membranes or channels, where cellulose-based materials, with their structural tunability and surface chemistry, offer a versatile platform. The driving force in OEGs originates from the chemical potential difference between two electrolyte reservoirs of unequal ionic strength. When an ion-selective membrane bridges these reservoirs, the preferential migration of either cations or anions produces a net charge transport, establishing a potential difference that can be converted into electrical energy through an external circuit.

For an effective OEG, membranes must combine high ion selectivity with sufficient ionic permeability. Upon appropriate modification (e.g., sulfonation or carboxylation), cellulose acquires fixed surface charges that induce EDL formation within its nanochannels. The resulting EDLs repel co-ions and enrich counterions, imparting charge-selective transport properties. When the channel diameter approaches or falls below the λ_D_, EDL overlap suppresses co-ion penetration and enables predominantly unidirectional counterion migration. This EDL-driven ion selectivity is essential for sustaining a net ionic current under a salinity gradient.

In saline-driven systems, the dominant charge carrier depends on both the surface charge polarity of the nanochannels and the ionic mobility of the electrolyte ions. For negatively charged cellulose nanochannels, cations are selectively transported, and protons (H⁺) can play a significant role because of their exceptionally high mobility. In hydrophilic nanochannels with hydrogen-bond networks, proton transport may occur via the Grotthuss mechanism, whereas at higher ionic strength or in larger channels, ion transport is typically dominated by the conventional vehicular diffusion of ions such as Na⁺.

In cellulose-based OEGs, maximizing the EDL efficiency requires tuning the zetapotential via surface functionalization (e.g., TEMPO-mediated oxidation), adjusting pore diameters toward λ_D_ to promote diffuse-layer overlap, and balancing the ion selectivity with permeability—design principles that bridge fundamental electrokinetics and device-level performance. A key challenge in OEG membrane design is this selectivity—permeability trade-off: narrow channels enhance the selectivity via EDL overlap but impede the ionic flux owing to increased resistance, whereas larger pores enhance conductivity but reduce the selectivity due to diminished interfacial interactions. Cellulose materials enable the navigation of this trade-off via hierarchical structuring, including gradient porosity or layered architectures that combine selective and conductive regions. Several strategies can be employed to increase the power density in cellulose-based OEG devices: (1) charge-density optimization by increasing the fixed-charge sites to strengthen EDL effects without excessively constricting the transport pathways; (2) gradient structuring by imposing concentration or pH gradients across the membrane to reinforce ionic asymmetry; (3) hybrid composite engineering through the incorporation of inorganic nanoparticles (e.g., graphene oxide (GO) and MOFs) to modulate the charge and channel morphology; and (4) multilayer designs employing asymmetric cellulose membranes to create directional ion-transport pathways.

### Mechanism of DEGs

In an early study demonstrating electricity generation from the sliding of ILs on graphene, DEGs leveraged the dynamic interaction between moving water droplets and charged solid interfaces to induce a transient charge redistribution and generate an electrical output (Fig. 4f). When an electrolyte droplet contacts a charged surface, an EDL forms spontaneously at the liquid–solid interface, consisting of surface-bound ions and a diffuse layer of counterions in the adjacent liquid. As the droplet moves across the surface, either by rolling, sliding, or oscillating, the EDL shifts spatially, causing temporal variations in the local charge density and producing a potential difference along the droplet trajectory. This moving EDL boundary behaves analogously to a pseudocapacitor, where charge accumulates at the advancing edge and is released at the trailing edge, driving a net electron flow through the external circuit. The induced voltage depends on droplet the velocity, ionic concentration, and strength of the interfacial electrostatic interactions between the substrate and ions.

Beyond moving-EDL-boundary effects, DEGs based on electrostatic induction have been extensively studied. In such devices, electrets are often incorporated to enhance the performance, and various electrode configurations have been developed to improve charge collection. Cellulose-based DEG devices can similarly exploit electrostatic induction by integrating electret components or charge-storage layers. For example, when a dielectric cellulose film is triboelectrically charged and subsequently contacted by a droplet, repeated contact–separation cycles induce alternating polarization and relaxation, producing a pulsed current output.

HEGs can be described within a unified thermodynamic framework governed by gradients in chemical potential (∇μ), which drive both interfacial charge generation and subsequent charge transport. Within this perspective, different types of HEGs represent distinct manifestations of chemical potential gradients at solid–liquid interfaces. Specifically, MEGs and EEGs are primarily driven by gradients in water chemical potential associated with adsorption and evaporation processes, respectively. OEGs originate from ion chemical potential gradients induced by salinity differences and ion-selective transport in nanochannels. In contrast, DEGs are governed by transient variations in interfacial free energy during dynamic liquid–solid contact and separation, which can be interpreted as time-dependent interfacial chemical potential gradients. Despite sharing common electric double layer and nanofluidic principles, these systems differ in their dominant driving forces and rate-limiting steps, leading to distinct energy-transduction pathways under a unified chemical potential-driven framework.

### Chemical Principles in Cellulose-based HEGs

Electricity generation in cellulose-based hydrovoltaic systems arises from interfacial chemical processes that couple water transport to ionic charge separation at charged cellulose surfaces. Beyond the macroscopic device geometry, the output voltage, current density, and operational stability are fundamentally governed by surface ionization equilibria, hydrogen-bond-mediated ion transport, and EDL dynamics within hydrated cellulose networks. In this context, cellulose is not merely a passive scaffold but an active electrochemical transducer that converts gradients in water chemical potential into electrical energy via a coordinated sequence: surface ionization → water–ion interactions → ion migration → charge separation → electrical output. This general mechanism applies across all four HEG types, with the principal differences arising from the specific driving force.

#### Interfacial Chemistry of Cellulose

Cellulose is a polyhydroxylated biopolymer whose surface –OH groups—and, when chemically modified, –COOH or –SO_3_H functionalities—can partially ionize in aqueous environments (e.g., R–COOH ⇌ R–COO⁻ + H⁺). Such deprotonation generates fixed anionic sites on the cellulose framework and mobile counterions in solution, thereby establishing an EDL at the cellulose–water interface. For a symmetric monovalent electrolyte, the equilibrium electrostatic potential ψ(*x*) along the surface normal *x* is described by the Poisson–Boltzmann equation [86, 87]:

4 $$\frac{{d}^{2}\psi }{d{x}^{2}}=\frac{2{n}_{0}e}{{\varepsilon}_{0}{\varepsilon}_{r}}sinh\left(\frac{e\psi }{{k}_{\mathrm{B}}T}\right)$$

where *ψ* is the electric potential (V), *n*_0_ is the bulk number density of monovalent ions (m^−3^), *e* is the elementary charge (1.602 × 10^−19^ C), *ε*₀ is the vacuum permittivity (8.85 × 10^−12^ F m^−1^), ε_r_ is the relative permittivity of water (≈78 in bulk), *k*_B_ is the Boltzmann constant (1.38 × 10^−23^ J K^−1^), and T is the absolute temperature (K).

The Navier–Stokes equation, coupled with electrostatic body forces, describes fluid flow in nanochannels:

5 $$\rho \left( {\frac{{\partial {\mathbf{u}}}}{{\partial t}} + {\mathbf{u}} \cdot \nabla {\mathbf{u}}} \right) =  - \nabla p + \eta \nabla ^{2} {\mathbf{u}} + {\mathbf{F}}_{e}$$

where ρ is the fluid density, u is the velocity field, p is the pressure, η is the dynamic viscosity, and F_*e*_ represents the body force, which typically includes the electrostatic force (ρeE) in charged nanochannels. This equation enables the prediction of flow-induced charge transport and streaming current.

Ion transport in hydrovoltaic systems can be described by the Nernst–Planck equation, which accounts for diffusion driven by concentration gradients, migration under electric fields, and convection induced by fluid flow:

6 $${\mathbf{J}}_{\mathrm{i}}=-{D}_{i}{\nabla}_{{c}_{i}} - \frac{{z}_{i}e{D}_{i}}{{k}_{B}T}{c}_{i}\nabla \psi + {c}_{i}\mathbf{u}$$

where J_i_ is the ionic flux, D_i_ is the diffusion coefficient, c_i_ is the ion concentration, z_i_ is the ionic valence, e is the elementary charge, k_B_ is the Boltzmann constant, T is the temperature, ψ is the electric potential, and u is the fluid velocity. The three terms represent diffusion, electric migration, and convection, respectively. In this framework, the ion flux is governed by the combined effects of concentration distribution, electrostatic potential, and fluid velocity. Coupled with the Poisson–Boltzmann equation for electric double-layer structure and the Navier–Stokes equation for fluid dynamics, the Nernst–Planck equation provides a comprehensive theoretical basis for understanding ion transport and charge generation in hydrovoltaic systems.

This EDL provides a reservoir of mobile charge that can be converted into electrical output under nonequilibrium water transport. The characteristic EDL thickness, quantified by the Debye length λ_D_ (as defined above), decreases with increasing ionic strength I [88]. When cellulose pore diameters or interfibrillar spacings approach λ_D_ (typically 1–10 nm), overlapping EDLs promote counterion selectivity, suppress co-ion transport, and thereby enhance charge separation and open-circuit voltage. The electrostatic potential at the shear plane, ζ (typically − 20 to − 60 mV for oxidized celluloses), directly modulates electrokinetically generated currents and therefore serves as a key descriptor linking interfacial chemistry to device performance.

Hydrogen-bond networks between cellulose chains and adsorbed water further stabilize oriented dipoles and facilitate proton transport via the Grotthuss mechanism. At the device scale, the resulting proton conduction can be expressed as an effective drift current,

7 $$J=ne{\mu}_{{H}^{+}}E\approx {\sigma}_{\mathrm{ion}}E$$

which relates the carrier density n (m^−3^), proton mobility μ_H⁺_ (m^2^ V^−1^ s^−1^), electric field E (V m^−1^), and ionic conductivity σ_ion_ (S m^−1^). Surface modification can tune the fixed-charge density *ρ*_f_ (C m^−2^), wettability, and local dielectric constant *ε*_r_, thereby adjusting both the zeta potential ζ and effective EDL capacitance, C_EDL_ = *ε*₀*ε*_r_A/d, where A is the interfacial area (m^2^) and d is an effective charge-separation distance (m). Collectively, these parameters govern both transient charge accumulation and steady-state electrical output in cellulose-based HEG devices.

#### Chemical Driving Forces across Different HEG Devices

Although MEGs, EEGs, OEGs, and DEGs differ in their external stimuli, they share a common electrochemical basis: nonequilibrium perturbation of the EDL converts interfacial ion distributions into macroscopic electrical signals. The dominant chemical driving force in each mode dictates how surface charge, ion mobility, and water transport are coupled, thereby determining the attainable voltage, current density, and power output.

1. Chemical driving forces in MEGs

Water adsorption induces functional-group ionization and establishes a proton-concentration gradient,

8 $$\Delta \mu = RT{\text{ ln}}\frac{{a_{1} }}{{a_{2} }}$$

where R is the gas constant, T is temperature, and a denotes proton activity. The corresponding open-circuit voltage can be approximated as follows:

9 $$V\approx \frac{{k}_{\mathrm{B}}T}{e}ln\left(\frac{{a}_{{H}^{+},{\mathrm{moist}}}}{{a}_{{H}^{+},{\mathrm{dry}}}}\right)$$

which shows that the maximum output voltage in MEGs depends logarithmically on the humidity-induced contrast in proton activity. Proton transport within the hydrated cellulose follows Fick’s law:

10 $${J}_{{H}^{+}}=-{D}_{{H}^{+}}\frac{d{C}_{{H}^{+}}}{dx}$$

where *D*_H⁺_ and *C*_H⁺_ are the proton diffusion coefficient and concentration, respectively. Although the activity gradient primarily determines the voltage, the diffusion coefficient and effective transport length govern the short-circuit current and response time. Sulfonated or TEMPO-oxidized celluloses, which provide high densities of weak acid sites, sustain a large Δμ, whereas chemically patterned or bilayer architectures stabilize the built-in electrochemical gradients and improve the output reproducibility.

(2) Chemical driving forces in EEGs

In evaporation-driven generators, capillary flow induced by evaporation convects EDL counterions through charged pores, generating a streaming potential and current described by the Helmholtz–Smoluchowski relations introduced above. Here, capillary pressure ΔP can be approximated as follows:

11 $$\Delta P\approx \frac{2\gamma cos\theta }{r}$$

where γ is the surface tension, θ is the contact angle, and r is the pore radius.

These relations indicate that the output voltage scales with the zeta potential ζ and capillary pressure, whereas the current is additionally governed by the pore geometry and hydraulic resistance. Increasing ζ via surface oxidation or ionic coordination strengthens the electrokinetic coupling, while the wettability (θ) and pore radius (r) regulate both the magnitude and temporal stability of the evaporation-driven pressure gradient. Consequently, chemical modification tunes not only the instantaneous output but also the long-term operational durability.

(3) Chemical driving forces in OEGs

Under a salinity gradient, ion transport across charged cellulose membranes generates an electrochemical potential described by the Nernst relation:

12 $$E=\frac{RT}{zF}ln\left(\frac{{a}_{{\mathrm{hig}}{\mathrm{h}}}}{{a}_{\mathrm{low}}}\right)$$

where z is the ion valence, F is the Faraday constant, and a_high_/a_low_ are the ionic activities in the two reservoirs. This relation sets the theoretical upper limit of the open-circuit voltage. Within charged cellulose nanochannels, an additional Donnan potential arises:

13 $${\phi}_{D}\approx \frac{{k}_{\mathrm{B}}T}{e}ln\left(\frac{{C}_{+}+{C}_{f}}{{C}_{-}}\right)$$

where C_f_ denotes the fixed-charge concentration and C_±_ are the mobile ion densities.

Carboxylated or sulfated celluloses, often crosslinked with polycarboxylic acids, provide high C_f_ and nanochannel sizes comparable to λ_D_, promoting EDL overlap and nearly unipolar ion conduction that enhances the power density. Chemically asymmetric cellulose membranes further suppress back-diffusion, thereby improving the current rectification and energy-conversion efficiency.

(4) Chemical driving forces in DEGs

When a liquid droplet contacts a charged cellulose surface, such as a natural leaf epidermis, dynamic deformation of the EDL generates a transient current,

14 $$I=\sigma \frac{dA}{dt}$$

where σ is the surface charge density and dA/dt reflects the spreading/retraction velocity. The advancing edge accumulates a charge, whereas the receding edge releases it, producing an alternating electron flow. The waxy coating on the cellulose of a plant epidermis modulates *θ* and *σ*, thereby controlling charge-transfer kinetics. A low ionic strength maximizes *σ* by minimizing electrostatic screening, whereas high salinity compresses EDL and reduces the voltage. The underlying cellulose tissue, which is rich in hydroxyl and carboxyl groups, provides ionic conduction pathways that connect the droplet interface to the electrodes.

#### Environmental and Chemical Modulators

Environmental and chemical conditions play a decisive role in regulating the stability, reproducibility, and efficiency of cellulose-based HEGs by modulating interfacial charge regulation and EDL structure. The surface charge density of cellulose follows a site-binding relation,

15 $${\sigma}_{s}=e{N}_{s}\left({\alpha}_{+}-{\alpha}_{-}\right)$$

where N_s_ (sites m^−2^) is the surface site density, and α_+_ and α_−_ denote the protonation and deprotonation fractions governed by pH and the intrinsic pKa values of surface functional groups. Accordingly, pH directly tunes the zeta potential: lower pH favors protonation, decreasing negative surface charge and weakening electrokinetic coupling, whereas higher pH promotes deprotonation, increases ζ, and strengthens ion-selective transport. Consequently, pH largely determines the attainable open-circuit voltage in MEGs and EEGs and the extent of ion selectivity in OEGs. For example, in cellulose-based wood nanochannel EEG systems, tuning the pH enabled output voltages approaching ~ 1 V, highlighting the strong dependence of hydrovoltaic output on electrolyte conditions [39].

Ionic strength provides a second, equally important lever by setting the Debye screening length (*λ*_D_ ∝ I^−1/2^). In dilute electrolytes, extended EDLs promote overlap within cellulose nanochannels, enhancing counterion selectivity and amplifying electrostatic potentials across HEG modes. Conversely, high salinity compresses EDL, reduces effective charge separation, and diminishes voltage output, particularly in DEG and OEG configurations. These trends explain why identical HEG architectures can exhibit markedly different performances under varying electrolyte conditions. A flexible horizontal hydrovoltaic device was reported by Ge et al., where the output voltage progressively decreased with increasing NaCl concentration from 10⁻⁷ to 1 M. This strong dependence on ionic strength highlights its potential for selective ion sensing and real-time monitoring of sweat electrolytes [89].

Temperature further modulates hydrovoltaic behavior through its effects on fluid viscosity (η) and dielectric constant (*ε*_r_). Higher temperatures reduce viscosity and increase ion mobility, thereby enhancing current density in diffusion- and flow-driven modes, while concomitant changes in *ε*_r_ modify EDL capacitance and charge-storage dynamics. However, excessive temperature fluctuations can destabilize hydration equilibria, highlighting a trade-off between instantaneous output and long-term operational stability. For instance, Tan et al. reported a hydrovoltaic generator constructed by integrating a LiCl-impregnated cellulose paper, which enhances moisture uptake, with a carbon-black-coated cellulose layer that facilitates evaporation. With increasing temperature from − 5 to 25 °C, the device output voltage rose markedly from ~ 0.3 V to above 0.7 V. A further temperature increase to 35 °C led to a slight decline in voltage, with a reduction of approximately 0.02 V [37].

Collectively, pH, ionic strength, and temperature are not merely external perturbations; they function as controllable design variables that determine the magnitude and temporal stability of hydrovoltaic output by modulating surface charge density, EDL overlap, and electrochemical driving forces. By deliberately tuning these chemical parameters—rather than relying exclusively on structural optimization—cellulose-based HEGs can achieve improved reproducibility, reduced performance drift, and enhanced durability across diverse operating conditions.

#### Unified Framework and Design Guidelines

From a theoretical perspective, hydrovoltaic phenomena in nanochannels are commonly described by coupled electrokinetic equations, including the Poisson–Boltzmann equation, the Navier–Stokes equation, and the Nernst–Planck equation. The Poisson–Boltzmann equation describes the distribution of ions and the formation of the electrical double layer near charged surfaces. The Navier–Stokes equation governs fluid flow in nanochannels driven by pressure, capillary forces, or evaporation. The Nernst–Planck equation describes ion transport driven by concentration gradients, electric fields, and fluid flow. The coupling of these equations forms the basis of electrokinetic models used to describe streaming potential, ion diffusion, and charge transport in hydrovoltaic systems. These theoretical frameworks provide useful tools for predicting device performance and guiding material and structural design.

Across HEG devices, three chemically tunable factors dominate performance: (1) surface charge density (ρ_f_), which can be increased via oxidation or sulfonation to enhance ζ and strengthen EDL interactions; (2) hydrophilicity–hydrophobicity balance, which regulates water flux and mitigates oversaturation; and (3) ionic conductivity (*σ*_ion_), which can be improved through hydrogen-bond-mediated pathways or by incorporating ILs, polyelectrolytes, or MXenes.

Their combined influence can be qualitatively expressed as follows:

16 $${V}_{\mathrm{out}}\propto \zeta \left(\frac{dA}{dt}\right)f\left({\lambda}_{D},{L}_{p}\right)$$

where V_out_ is the output voltage, ζ is the zeta potential, dA/dt represents the time-dependent wetting rate (or, equivalently, the water flux in MEGs and EEGs), λ_D_ is the Debye length, and L_p_ is the characteristic pore length. Dimensionless function f(λ_D_, L_p_) captures nanoconfinement and EDL-overlap effects, highlighting that chemical functionality actively governs electrohydrodynamic coupling rather than serving as a passive material attribute.

Precise chemical control, therefore, provides a fundamental route to advance cellulose-enabled hydrovoltaic technologies. Manipulating ionization equilibria, hydrogen-bond dynamics, and EDL overlap enables quantitative tuning of output voltage, current density, and long-term durability. Future studies integrating in situ spectroscopic characterization with Poisson–Nernst–Planck and Born-solvation modeling are expected to further elucidate how molecular-level chemistry determines macroscopic power generation, thereby enabling the rational design of chemically optimized, sustainable cellulose materials that integrate multiple hydrovoltaic mechanisms within a unified, eco-friendly framework.

#### Redox Effects by Non-inert Electrodes

The employment of reactive metal electrodes (e.g., Al, Zn, Cu, Mg, and Fe) can frequently introduce Faradaic reaction, resulting in combining the intrinsic hydrovoltaic effect. In cellulose-based systems, the porous and hygroscopic nature of the matrix often facilitates the formation of an unintentional electrolyte bridge between electrodes. When non-inert metals are used, the device may inadvertently operate as a galvanic cell, where the oxidation of the metal anode (e.g., Cu → Cu^2+^ + 2e^−^) and the reduction of oxygen or water at the cathode generate a spontaneous electrochemical potential. These Faradaic processes often yield significantly higher output voltages than pure hydrovoltaic mechanisms, leading to overestimated performance metrics that reflect battery-like chemical consumption rather than sustainable energy harvesting from environmental moisture. Furthermore, many reported high-performance HEGs using reactive electrodes fail to maintain output when the metal is replaced with symmetric inert electrodes, confirming that the chemical potential difference of the electrodes, rather than the cellulose-water interaction, is the primary driver.

Therefore, for the distinct assessment of cellulose-enabled HEGs, the adoption of standardized inert electrodes is imperative. Materials such as gold (Au), platinum (Pt), or high-quality carbon-based electrodes such as graphite and carbon cloth should be employed to minimize interfacial Faradaic interference. When reactive electrodes are utilized to create complex systems, researchers must clearly delineate the contribution of redox energy from the intrinsic hydrovoltaic output through control experiments, such as testing under inert gas environments or using symmetric electrode configurations. This methodological rigor is essential for advancing the fundamental understanding of cellulose-water interactions and ensuring the reproducibility of next-generation hydrovoltaic technologies.

## Advances in Cellulose-enabled HEGs

Cellulose is among the most widely used biopolymers in HEG devices owing to its low cost, processability, biocompatibility, and biodegradability. Its hydrophilicity, arising from its abundant hydroxyl groups, promotes efficient moisture adsorption and strong affinity for water.

**Fig. 5:**
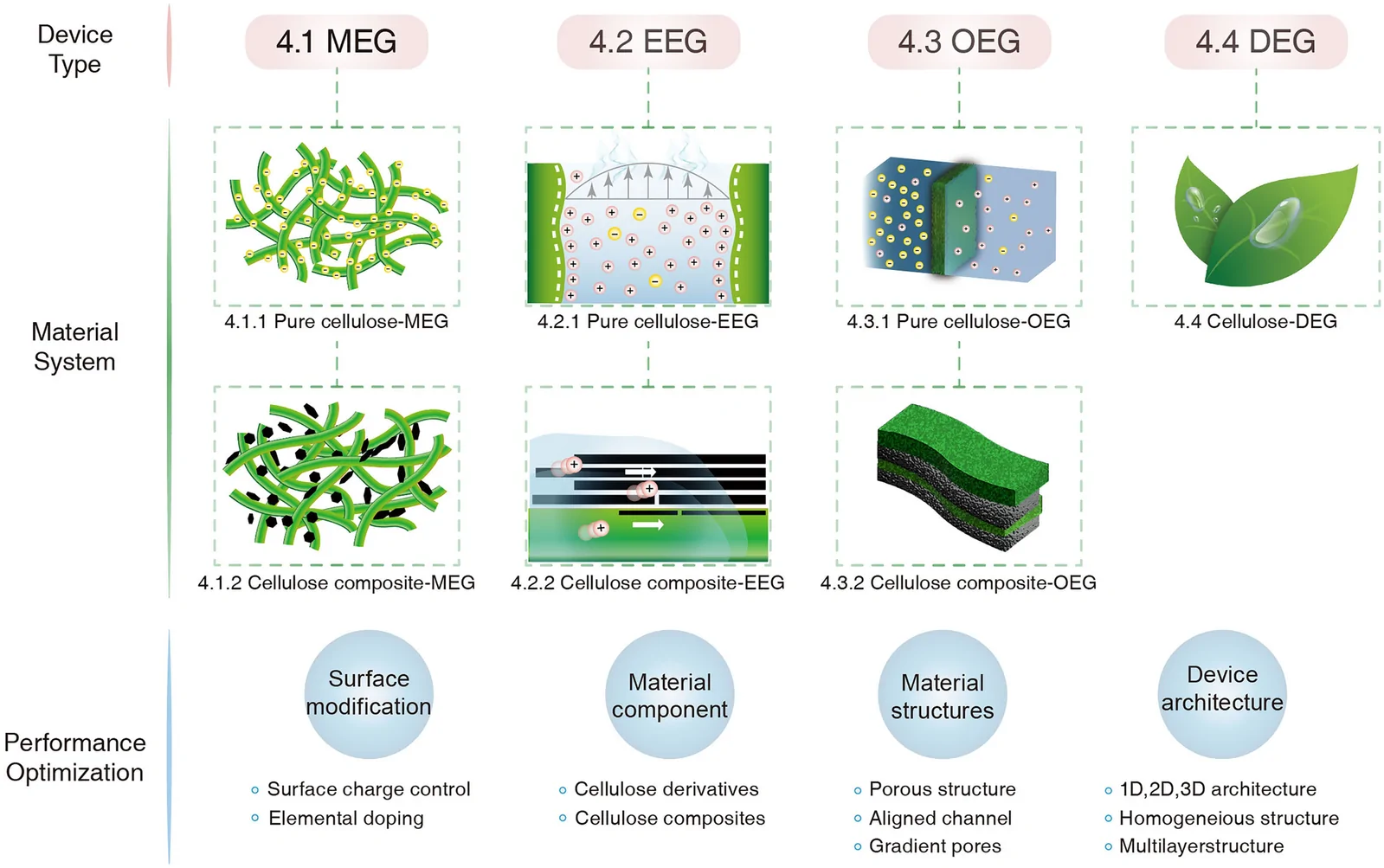
Schematic overview of the organizational structure of Section 4 in this review

Section 4 systematically categorizes cellulose-based HEGs according to their dominant charge-generation mechanisms and associated material design strategies. As summarized in Fig. 5, HEGs are classified into four primary types: MEGs, EEGs, OEGs, and DEGs. Each type corresponds to a distinct water–solid interaction pathway governing ion migration and potential difference formation. To clarify the materials hierarchy, each category includes pure cellulose systems that exploit the intrinsic surface chemistry and morphology of cellulose, as well as cellulose composite systems in which functional components are introduced to enhance ionic transport and output stability. This section also discusses performance-optimization strategies—including surface modification, material composition, material structures, and device architectures—providing a comprehensive framework for understanding how cellulose design governs hydrovoltaic behavior under diverse environmental stimuli.

### Cellulose-enabled MEG

Recent advancements in cellulose-based MEGs have focused on optimizing surface ionization and ion diffusion kinetics to drive electrical output (Table 1). In pure cellulose-based MEGs, research has prioritized overcoming low-power densities through nanomorphology engineering and the implementation of anisotropic architectures to facilitate efficient charge separation. Simultaneously, chemical modifications are strategically employed to mitigate structural collapse caused by water-induced swelling, ensuring long-term operational stability. To bypass the inherent performance bottlenecks of single-component materials, cellulose composite-based MEGs have emerged as a more direct solution. By integrating conductive nanomaterials, organic frameworks, or biomimetic structural designs, these hybrids leverage the excellent hygroscopicity of cellulose while significantly accelerating charge transport. These hybrid systems synergistically combine the intrinsic hygroscopicity of cellulose with accelerated charge-transport properties, transcending the performance ceilings of single-component materials and enabling the development of high-performance, multifunctional hydrovoltaic devices.

**Table 1:** Summary of cellulose-enabled MEGs

Active material	Structure	Device structure	Voltage (V)	Current density (μA cm^−2^)	Power density (μW cm^−2^)	Electrode	Condition	Mark	References
CNF/CNT/Citric acid	Aerogel	Bilayer	0.703	39	28.9	Cu/Cu	RH 30%	Biodegradation, crosslinking	[40]
CNT and CMC composite//CNT and quaternary ammonium salted cellulose (Q-CNF)	Aerogel	Asymmetric bilayer aerogel	0.668	6.4	0.871	Al/Ag	RH 90%		[90]
Covalent organic framework (COF-2SO_3_H) into a carboxylated CNF network (CNF-C)	Aerogel	Coin-type	0.55	0.34	0.212	Stainless-steel mesh (SS)	RH 100%		[91]
MOF/modified CNF	Aerogel	Monolayer	0.302	3.7	1.12	C@Al/C@Al	RH 99%		[92]
TEMPO-CNF/Quatern-CNF	Aerogel	Bilayer	0.115	0.045	–	Pt/Pt	RH 99%		[93]
TEMPO-CNF	Aerogel	Monolayer	0.11	0.022	0.0024	Pt/Pt	RH 99%		[32]
CNT-Chitosan CNF PVA	Aerogel (trilayer)	Trilayer	1.15	15.87	32.59	Ni/Ni	RH 80%	Solar desalination, double-gradient structures	[94]
CA/carbon black	Column (Fiber)	Monolayer	0.195	98.65 μA	4.78 μW	C/C	RH 50%	Cost effective using cigarette	[95]
CA	Electrospun membrane	Monolayer	0.7	3.5	2.45	Al/Au@SS	RH 90%		[96]
CA	Electrospun membrane	Monolayer	0.11	80	0.0084	Ag/Ag	RH 50%	Research on porous structures and performance	[97]
TEMPO-CNF/MXene/Polydopamine	Film	Monolayer	0.54	–	–	Cu/Al	RH 91%		[98]
Bacterial cellulose/MWNT	Film	Monolayer	0.84	2.21	0.163	Al/Cu	RH 63–77%		[99]
Cellulose filter paper/carbon black	Filter paper	Monolayer with two electrodes on same side	0.803	13 μA	2.63 μW	Cu/Zn	RH 97%		[100]
Aminated regenerated CNF	Film	Monolayer by moist from saturated salt solution	4.2	0.008	0.033	Al/Al	RH 85%	Crosslinking	[101]
GO/CNF	Film	Monolayer with two electrodes on same side	0.25	0.11	0.2	C/C	RH 30%	CNF used for moisture capture	[102]
CNF/PSSA/MXene	Film	Monolayer	0.3	1.2	0.073	Ag/Ag	RH 80%		[103]
GO/CNF composite	Film	Monolayer		0.286	–	Ti/Ti	–	–	[104]
CNF/PVA/Clay/AlCl_3_	Hydrogel	Monolayer	0.078	–	–	Cu/Cu	–	Self-healing	[105]
MXene/CNC composite double-networked with tamarind gum/polyacrylamide	Hydrogel	Monolayer	0.164	–	–	Cu/Cu	–	Deformable	[36]
Sulfated CNF (SCNF)/PVA	Hydrogel	Monolayer	0.9	92	24	Cu/Pt	RH 80%		[106]
PVA/CNT//PVA/CNF/PA/Li	Hydrogel (Bilayer)	Bilayer	1	600 μA	26.5	Pt/Al	RH 80%		[107]
CNF/poly(ionic liquid)/ionic liquid	Ionic gel	Monolayer	0.3	10 μA	516 μW cm^−3^	Ag/AgCl	RH 70%	Biodegradable in soil	[108]
AgNW coating on CA	Membrane	Monolayer	0.302	3.7	–	Cu/Cu	RH 45%		[109]
LiCl-impregnated ionic wood	Wood	Sandwich	0.75	712 μA	–	Ag/Ag@Cu	RH 86–96%		[37]
Paper impregnated with citric acid and surface-coated with MXene	Paper	Monolayer	0.275	7.6	2.1	Cu/Cu	RH 73%		[110]
Cellulose paper coated with graphite	Paper	Monolayer	0.5	0.25 μA	0.73 μW		RH 60%		[111]
FeCl_3_-wood-based PVA/chitosan/AMPS hydrogel	Wood	Monolayer	0.066	–	–	Cu/Cu	–		[112]
Ionic wood	Wood (Bilayer)	Bilayer	0.57	77 μA	0.71 μW cm^−3^	Pt/Pt	RH 85%		[113]
BC/reduced GO bilayer	Film	Bilayer	1.08	53.80 μA	3.06	Zn/Cu	RH 90%		[114]
CMC/MXene/Al^3+^ composite	Film	Monolayer	0.063	–	–	Cu/Cu	RH 90%	Crosslinking	[115]
CNF crosslinked with Al^3+^	Aerogel	Monolayer	0.95	112	106.1	Zn/SS	RH 95%	Crosslinking (Al^3+^ ion)	[116]
carboxymethylated CNF (CM-CNF)/quaternary ammonium-CNF (Q-CNF) bilayer	Film (bilayer)	Bilayer	0.7	0.807 μA	–	C/C	RH 95%		[117]
Carboxylated nanofibrillated cellulose (CNF)/polyglutamic acid (PGA)/citric acid/NaCl	Hydrogel	Monolayer	0.8	30 μA	–	Ag/C	Between 20 and 55%	Crosslinking (Na^+^ ion), long-term stability maintaining > 95% performance for ∼1000 h	[118]
CNC/PVA	Amorphous slurry	Monolayer	0.76	621	32	Ag/C	RH 90%	Scale-up by screen-printing	[119]
CBs/PVA-paper	Kirigami patterned paper	Monolayer	1.2	6.0 μA	0.25	Al/Al	RH 45%	Stretchability	[120]
CMC/citric acid/delignified pomelo peel	Hydrogel	Monolayer	1.51	740.5	101.1	Cu/Cu	RH 90%	Nanofluidic hydrogel with sub-Debye length	[121]
Poly(4-styrenesulfonic acid)/GO/Glycerine/PVA coated fabric	Fabric	Monolayer	0.55	7.08	1.14	Liquid metal-C/Ag	–		[122]
Lithium-cellulose/PVA	Film	Bilayer	1.0	0.8mA	1.506mW cm^−3^	Zn-Zn	RH 70%		[123]

#### Pure Cellulose-based MEGs

**Fig. 6:**
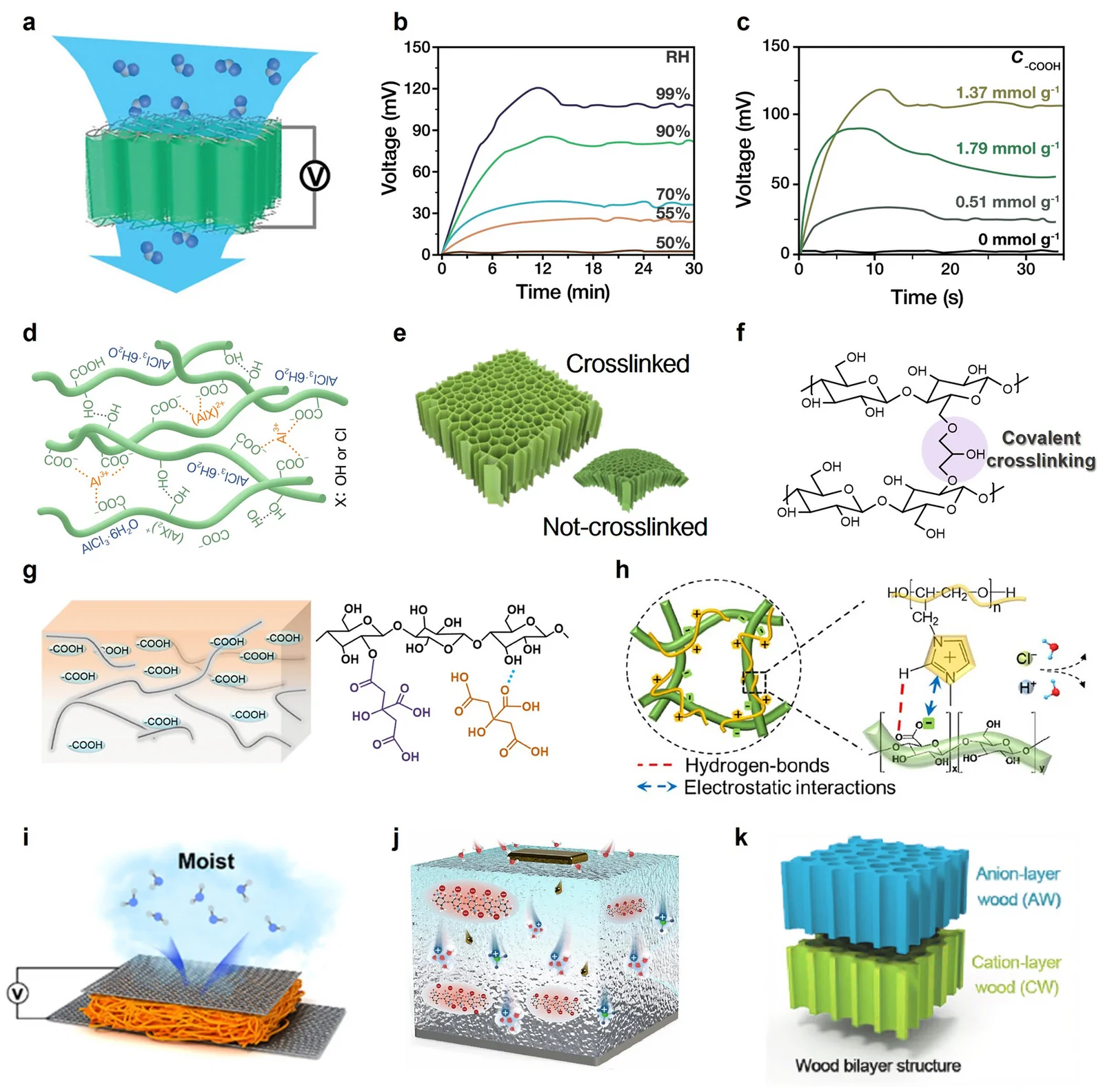
Pure cellulose-based MEGs. **a** Schematic of TEMPO-CNF-aerogel-based MEG device. **b** Voltage generation of a TEMPO-CNF-aerogel-based MEG under varying RH. **c** Voltage generation of a TEMPO-CNF-aerogel-based MEG at different carboxylic groups. **a**–**c** Reproduced with permission [32]. Copyright 2019, Wiley–VCH. **d** Proposed structures of CNF aerogels crosslinked with Al(III) loading. **e** Proposed structures of crosslinked and non-crosslinked CNF aerogels under moist airflow and ambient drying. **d-e** Reproduced with permission [116]. Copyright 2025, Elsevier. **f** Chemical structure of covalently crosslinked cellulose by epichlorohydrin. Reproduced with permission [40]. Copyright 2024, Royal Society of Chemistry. **g** Schematic of an organic-acid-gradient paper fabricated via a gradient-evaporation strategy using citric acid. Reproduced with permission [110]. Copyright 2022, American Chemical Society. **h** Schematic of CNF incorporated with poly(ionic liquid) (PIL) for free mobile ions. Reproduced with permission [108]. Copyright 2024, American Chemical Society. **i** Schematic of a MEG device fabricated with electrospun cellulose acetate (CA) nanofiber membrane. Reproduced with permission [97]. Copyright 2020, American Chemical Society. **j** Schematic of a nanocellulose-based hydrogel MEG. Reproduced with permission [106]. Copyright 2024, Elsevier. **k** Schematic of a bilayer-structured, wood-based MEG. Reproduced with permission [113]. Copyright 2022, American Chemical Society

Cellulose is a representative hygroscopic material owing to its hydroxyl groups that interact with atmospheric moisture via hydrogen bonds [124]. In CNF networks, MEGs exploit the intrinsic hydrophilicity and surface charges of cellulose fibrils to capture water vapor and form hydrated nanochannels throughout the porous matrix (Fig. 6a) [32]. CNFs contain abundant –COOH and –OH groups that readily adsorb water molecules, promoting the formation of continuous aqueous pathways. As RH varies, these hydrophilic networks establish ion-concentration gradients primarily governed by proton (H⁺) diffusion through the nanostructured cellulose framework. In some devices, MEG and EEG effects may coexist. When a humidity gradient is imposed (e.g., when one side is exposed to airflow), sustained adsorption on the wetter side and evaporation from the drier side drive convective water transport through the nanochannels. Consequently, CNF aerogels exposed to high-RH air develop an open-circuit voltage (V_oc_) that increases with RH (Fig. 6b). For example, under a modest airflow (~ 15 cm s^−1^), the output is negligible below ~ 55% RH, whereas at ~ 99% RH V_oc_ reaches approximately 0.1–0.11 V. This pronounced humidity dependence indicates that water availability and ion mobility are the key limiting factors in cellulose-based MEGs. However, excessive chemical modification can be counterproductive, potentially leading to performance degradation. For instance, an overabundance of carboxyl (–COOH) groups on TEMPO-oxidized cellulose nanofibers (TEMPO-CNFs) has been shown to decrease Voc at high-RH levels. This reduction is primarily attributed to over hydration and the subsequent structural collapse of the material (Fig. 6c). Accordingly, performance-enhancement strategies focus on increasing moisture uptake and ionic charge density through strategic engineering for an optimal balance between structure and performance.

An effective modification approach is to introduce hygroscopic salts and multivalent cations into the CNF network. Zhu et al. loaded CNF aerogels with AlCl₃·6H₂O, whose deliquescent nature markedly enhances moisture absorption. Concurrently, Al^3+^ ions coordinate with cellulose hydroxyl groups and serve as ionic crosslinkers that reinforce the fibrillar network (Fig. 6d, e) [116]. The resulting Al(III)-crosslinked CNF aerogels exhibit higher water uptake and improved structural stability in humid environments, enabling more stable and higher voltage outputs under repeated moisture exposure cycling. Notably, such metal–ion coordination can strengthen the cellulose framework without fully obstructing the nanoscale porosity. Recent studies have indicated that multivalent cations (e.g., K^+^, Ca^2+^, and Al^3+^) can bridge adjacent CNFs while preserving open ionic channels and, in some cases, may even facilitate ion transport by forming subnanometer coordination pathways. This behavior contrasts with overly dense covalent crosslinking, which can reduce the free volume.

The covalent crosslinking (e.g., using epichlorohydrin) can permanently stabilize cellulose networks against moisture-induced swelling or dissolution (Fig. 6f) [40]. However, excessive chemical modification to crosslink between cellulose chains can decrease the number of hydrophilic sites and the availability of nanochannels for water transport. Therefore, a balanced crosslinking strategy is therefore essential: a low degree of covalent crosslinking can improve the moisture tolerance and durability under an airflow while retaining sufficient porosity for rapid water diffusion and ion migration.

1. Free mobile ions for enhanced performance

Another powerful design strategy involves incorporating free mobile ions within cellulose matrices. This approach enhances the electrical output through the establishment of internal ionic gradients or by increasing the population of mobile charge carriers. Yang et al. implemented an organic-acid gradient in cellulose paper by asymmetrically infusing and drying citric acid (Fig. 6g) [110]. Citric acid was concentrated on one side of the paper and ionized upon exposure to humidity, establishing a fixed gradient of carboxylate anions across the paper thickness. Even under uniform ambient humidity, this chemical asymmetry produced a continuous voltage output on the order of tens of millivolts; under an imposed humidity difference, the device generated up to ~ 0.275 V (with ~ 7.6 μA cm^−2^ current). The underlying driving mechanism was a self-sustained ionic diffusion potential: one side of the paper maintained a higher anion concentration (and associated proton release) than the other. Therefore, upon water uptake, an internal electric field formed without requiring an external thermal gradient. Such gradient-engineered cellulose membranes expand the MEG design space by leveraging built-in chemical potential differences using inexpensive, benign reagents (here, citric acid). Moreover, incorporating functional polyelectrolytes (PIL) into cellulose can supply mobile ions and fixed charges that strengthen electrochemical driving forces. For example, Li et al. fabricated a CNF–PIL ionogel by co-assembling a cationic PIL with CNFs via complex coacervation (Fig. 6h) [108]. PIL introduced a high density of mobile cations and anions, effectively increasing the charge-carrier population within the network, while also forming an entangled polymeric matrix that improved the mechanical integrity. The CNF–PIL ionogel produced substantially higher voltages than a CNF-only film, whereas CNF ionogels without PIL exhibited much lower V_oc_, confirming that PIL ionic groups were central to the enhanced generation mechanism. Collectively, enriching cellulose with fixed ionic species—either through small-ion gradients or polymeric ionic networks—offers a robust route for amplifying the moisture-induced electrical output.

(2) Nanostructure and morphology engineering

Beyond chemical modification, engineering the physical architecture of cellulose can markedly improve MEG performance. Electrospun nanofiber membranes represent one such strategy (Fig. 6i) [97]. Electrospun CA produces submicron fiber mats with high porosity and a tunable pore-size distribution. Lyu et al. demonstrated that the post-treatment of CA nanofiber mats (e.g., compression and annealing) to adjust porosity (~ 50%–60%) and reduce the mean pore diameter (< 250 nm) systematically increased the output voltage. Higher porosity increased air–solid interfaces for water adsorption, whereas nanoscale pores favored the formation of numerous EDL-containing nanochannels. An optimized electrospun CA membrane (~ 52.6% porosity; ~ 200 nm mean pore diameter) produced significantly higher V_oc_ than its less porous or larger-pore counterparts, generating sufficient output to power small electronics (e.g., calculators) and function as a breath-responsive sensor. This improvement was attributed to the rapid moisture uptake of the membrane and establishment of dense ionic pathways within its fine, interconnected pore network. Similarly, configuring cellulose-based materials as hydrogels (Fig. 6j) can exploit the advantages of a percolating, water-rich phase [106]. Hydrogels inherently contain continuous aqueous domains that facilitate ion transport; therefore, incorporating functional groups can further increase charge density and hygroscopicity. For example, Mo et al. developed a sulfated CNF hydrogel MEG in which introducing –SO₃H groups substantially increased the water uptake and ionic conductivity. The sulfated CNF hydrogel maintained a stable voltage output over hundreds of hours, enabled by a robust ionic network and internal moisture replenishment. These results illustrate how soft, water-rich cellulose architectures can deliver high and stable performance by acting as self-contained moisture reservoirs that continuously sustain EDL-driven power generation.

(3) Architectural anisotropy—bilayer structures

Cellulose-based systems can benefit from anisotropy deliberately introduced through a bilayer structure comprising two layers with distinct morphologies, surface charges, and chemical compositions [117, 125]. A representative example is the bilayer wood membrane reported by Cai et al. (Fig. 6k) [113]. In this design, a natural basswood slice (cut perpendicular to the growth direction) was selectively modified on one face, yielding a two-layer structure comprising a hygroscopic, functionally charged surface layer atop an unmodified wood layer. The intrinsically aligned cellulose channels in the wood provided directed pathways for ion transport, while the asymmetric surface chemistry promoted preferential water adsorption at the modified layer. This bilayer wood-based MEG spontaneously adsorbed moisture from the air and established an internal moisture gradient across the membrane thickness. At 85% RH it delivered approximately 0.57 V_oc_ without external stimuli beyond ambient humidity. The aligned nanochannels and abundant dissociable groups in the top layer created a built-in electric field during water migration, analogous to one-way ion transport in a porous scaffold. By leveraging a biogenic scaffold (wood), this device also offers low cost and scalability, underscoring the potential of architecturally engineered cellulose structures.

#### Cellulose Composite-based MEGs

Recent cellulose composite–based MEGs demonstrate that hybrid architectures can effectively overcome the intrinsic limitations of pristine cellulose by integrating conductive networks, ion-selective nanochannels, and gradient designs. In this section, we systematically examine representative strategies in which the incorporation of 1D or framework-type conductive components into cellulose matrices results in substantial enhancements in output voltage and current [126], and derived a materials design perspective from these developments. Furthermore, we highlight how hierarchical organization and synergistic interactions among composite constituents enable the simultaneous optimization of the electrokinetic performance, water-transport efficiency, and mechanical robustness in cellulose-based MEGs.

1. Incorporation of electrically conductive nanomaterials

**Fig. 7:**
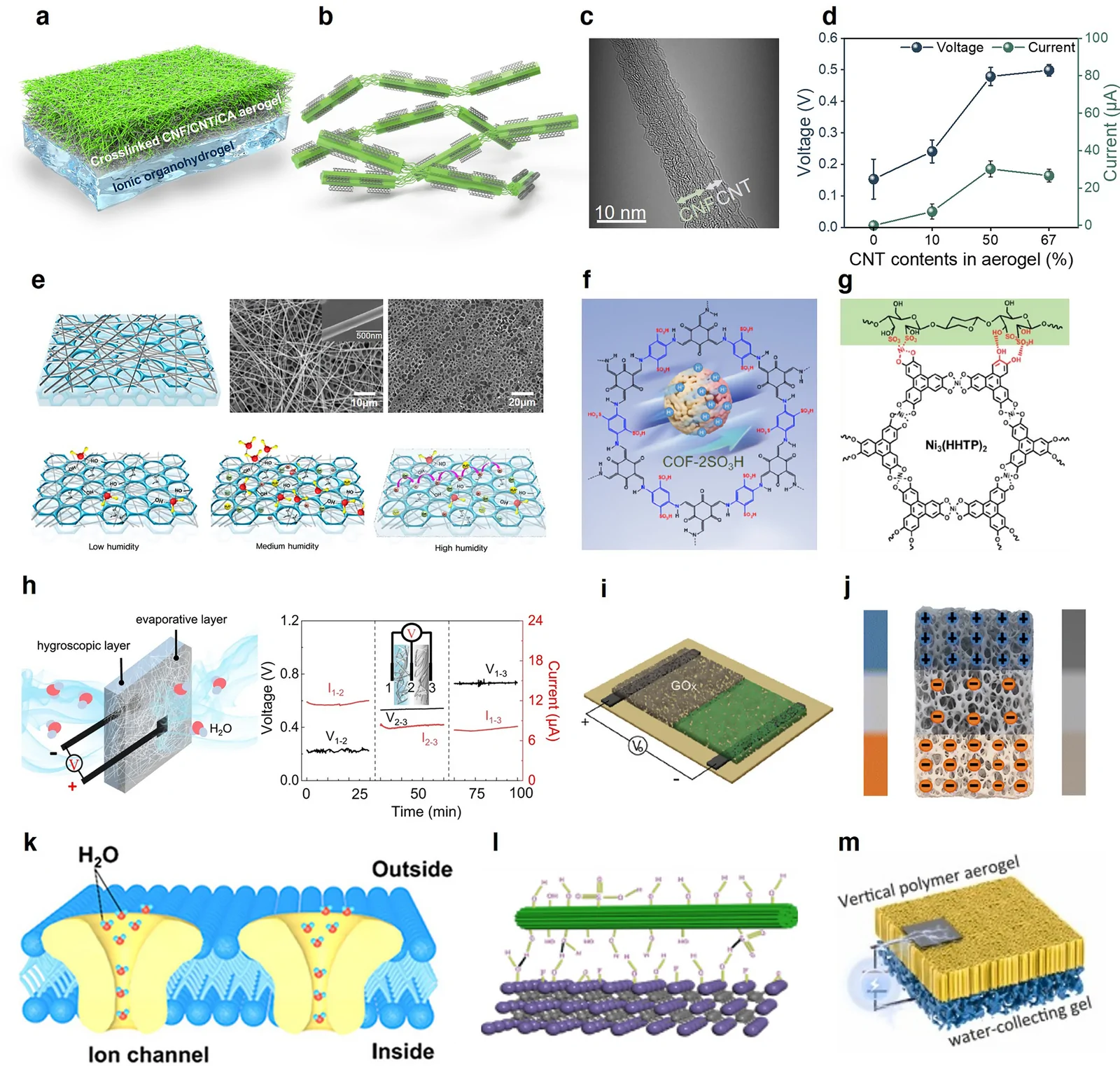
Cellulose composite-based MEGs. **a** Schematic of citric acid-crosslinked CNF/CNT aerogel-based MEG. **b** Schematic of a CNT–CNF composite. **c** TEM image showing CNT bundles aligned along the CNF surface. **d** Voltage and current enhancement as a function of CNT content in cellulose composite-based MEG. **a**–**d** Reproduced with permission [40]. Copyright 2024, Royal Society of Chemistry. **e** AgNW-coated cellulose membrane-based MEG. Reproduced with permission [109]. Copyright 2022, Elsevier. **f** Schematic of the COF-2SO_3_H structure for cellulose composite-based MEG. Reproduced with permission [91]. Copyright 2024, American Chemical Society. **g** Schematic of MOFs growth on cellulose fibrils and their mutual interactions cellulose composite-based MEG. Reproduced with permission [92]. Copyright 2022, Royal Society of Chemistry. **h** Schematic of a bilayer-structured MEG comprising a hygroscopic layer and an evaporative layer, with voltage and current shown as a function of electrode position. Reproduced with permission [37]. Copyright 2022, Springer Nature. **i** Schematic of a planar MEG device featuring horizontally arranged GOx and CNF layers. Reproduced with permission [102]. Copyright 2024, Wiley–VCH. **j** Schematic of a trilayer-structured MEG showing the ion-density and hydrophilicity gradients across the layers. Reproduced with permission [94]. Copyright 2023, Royal Society of Chemistry. **k** Biomimetic design of asymmetric CNF/GO architecture for directed moisture flow and enhanced ion-transport pathways. Reproduced with permission [104]. Copyright 2020, American Chemical Society. **l** Illustration of a robust hydrogen bonding between MXene and CNC enabling synergistic network formation. Reproduced with permission [36]. Copyright 2021, Elsevier. **m** MEG structure utilizing water-collecting gel of cellulose composite. Reproduced with permission [127]. Copyright 2024, Elsevier

Incorporating conductive nanocarbons into cellulose matrices is an effective strategy for enhancing both the mechanical integrity and electrochemical performance of MEGs. Park et al. reported a representative composite MEG in which CNFs were integrated with carbon nanotubes (CNTs) and chemically crosslinked using citric acid to stabilize the network (Fig. 7a) [40] In this design, CNFs provide a hydrophilic matrix that supports ion generation and promotes homogeneous CNT dispersion owing to their amphiphilic character. Transmission electron microscopy (TEM) revealed that CNTs were distributed as thin bundles along the CNF surfaces (Fig. 7b, c). The authors attributed this dispersion to the amphiphilic nature of cellulose, where the hydrophobic (200) planes enriched in C–H groups interacted with the CNTs via van der Waals forces in the water. Accordingly, van der Waals interactions between the hydrophobic (200) surface and sp^2^-carbon framework of the CNTs enabled effective dispersion without additional chemical functionalization. Increasing the CNT content markedly improved both V_oc_ and the short-circuit current (I_sc_); however, excessively high CNT loadings deteriorated the performance because of aggregation and impaired dispersion (Fig. 7d). Consistent with this trend, a pure CNF aerogel (0% CNT) generated only ~ 0.18 V, whereas introducing CNTs increased both the voltage and current output by establishing efficient electronic pathways and enhancing ion collection at the CNT surfaces. An optimal CNT loading was therefore required; an excessively high CNT content (e.g., 67 wt%) caused aggregation and reduced I_sc_ by hindering uniform dispersion and ion access. Overall, the crosslinked CNF/CNT composite integrated a robust, hydrophilic cellulose scaffold with a well-distributed conductive network, resulting in a substantially enhanced and more stable hydrovoltaic performance.

Incorporating metal nanomaterials is another effective approach to enhance charge transport in cellulose-based MEGs. Jiang et al. developed a cellulose-based MEG by integrating silver nanowires (AgNWs) into an asymmetric porous cellulose acetate (CA) membrane (Fig. 7e) [109]. The membrane exhibited a highly ordered honeycomb-like porous structure with enhanced hydrophobicity and pore regularity. AgNWs were drop-cast onto one side of the membrane to form a conductive nanowire network, producing an asymmetric bilayer configuration. This AgNW layer significantly increased electrical conductivity and provided continuous electron-transport pathways. This asymmetric configuration enabled moisture-driven electricity generation by facilitating proton dissociation at oxygen-containing cellulose groups and directing charge transport through the conductive AgNW network. Under continuous moisture exposure, the optimized MEG achieved a maximum V_oc_ of 302 mV and an I_sc_ density of 3.7 μA cm^−2^. The AgNW-cast cellulose MEG exhibited pronounced humidity-dependent behavior driven by proton dissociation and transport. At low humidity (15%–45% RH), strong hydrogen bonding restricted water mobility, limiting ion generation and producing weak output. At intermediate humidity (45%–75% RH), partial bond disruption enabled proton release and increased current. At high humidity (75%–90% RH), increased water adsorption and ionization enabled rapid proton hopping via the Grotthuss mechanism, yielding a sharp rise in voltage (up to 302 mV) and a fast response (~ 1.62 s). Collectively, these results demonstrated the high-humidity sensitivity and efficient moisture-induced energy conversion of the membranes.

(2) Organic framework for mechanical and electrical enhancements

Embedding covalent organic frameworks (COFs) within a cellulose matrix is another viable route for increasing ionic conductivity and enhancing moisture-to-electric conversion [128–131]. Xie et al. developed a CNF-based aerogel hybridized with a proton-conductive sulfonic acid-functionalized COF (COF-2SO_3_H), yielding a composite with hierarchical porosity, abundant surface functionality, and high ionic mobility (Fig. 7f) [91]. Carboxylated CNF (CNF-C) served as both a dispersion medium and a mechanical scaffold, enabling uniform distribution and immobilization of COF-2SO_3_H via hydrogen bonding. The embedded COF provided ordered proton-conducting nanochannels and a high density of ionizable –SO_3_H groups, which acted synergistically with the –COOH groups of the CNF-C to promote ion release and directional transport under a humidity gradient. The optimized COF-2SO_3_H/CNF-C aerogel exhibited significantly increased moisture uptake (approximately 1.6-fold relative to CNF-C) and an ionic conductivity of ~ 1.5 × 10⁻^3^ S cm^−1^ at 100% RH. In a coin-cell-type MEG configuration, the composite delivered a maximum V_oc_ of 1.0 V, an I_sc_ density of 0.75 μA cm^−2^, and a maximum power density of 0.212 μW cm^−2^ at 60 kΩ external resistance under ambient humidity. The output remained stable over repeated wet–dry cycling and across different electrode materials, supporting the conclusion that ion generation and conduction are intrinsic to the composite architecture.

Additionally, Li et al. enhanced the performance of cellulose-based MEGs by incorporating MOFs (Fig. 7g) [92]. They proposed a strategy to simultaneously harvest energy and potable water from atmospheric moisture using biohybrid fibrils composed of sulfonated CNFs coated with conductive and photothermal MOFs, such as Ni₃(HHTP)₂ or Ni₃(HITP)₂. The MEG device was fabricated through the oxidation and sulfonation of cellulose fibers, followed by chelation and MOF nucleation on the fiber surface, yielding hybrid fibrils with granular MOF shells and diameters of 15–32 nm. The resulting membranes exhibited hierarchical porosity and a high specific surface area (~ 234 m^2^ g⁻^1^), which significantly enhanced moisture uptake (~ 1.5 g g⁻^1^ at 99% RH) and electrical conductivity (up to 4.2 S m⁻^1^ under high humidity). The device generated a sustained V_oc_ of ~ 0.65 V and harvested water at up to 0.8 g g⁻^1^ h⁻^1^, while maintaining stable performance for over 18 h. Compared to neat CNF membranes and other nanofibrillar systems, the MOF-coated biohybrids delivered higher voltage output, attributable to enhanced hydration-driven ion dissociation and the intrinsic electronic conductivity of the MOF components.

Collectively, these COF- and MOF-based strategies demonstrate that integrating nanoporous ionic conductors into cellulose scaffolds not only amplifies hydration-driven charge generation but also enables sustainable, high-performance MEG platforms with multifunctional operation under real-world conditions.

(3) Structural engineering

Recent studies have shown that composite design strategies—including layered architectures, internal gradients, bioinspired channels, and reinforced networks—can significantly enhance moisture-electric generation in cellulose-based MEGs. Guo et al. developed a bilayer device comprising a hygroscopic, salt-infused cellulose layer laminated onto a hydrophobic evaporative layer (Fig. 7h) [37]. This bilayer cellulose-MEG leveraged coupled atmospheric moisture absorption and evaporation to sustain continuous water transport and long-term power generation. The hygroscopic layer, which was fabricated using cellulose paper impregnated with lithium chloride (LiCl), absorbed moisture from the air and subsequently released ions, thereby generating a potential difference. The evaporative layer, which consisted of cellulose paper embedded in carbon black, promoted rapid moisture evaporation. Together, these processes drove continuous water movement and prolonged electricity generation. This asymmetric structure maintained a persistent moisture gradient and continuous interfacial water transport, yielding stable outputs (~ 0.78 V, 7.5 μA for > 10 days) under ambient conditions. Electrodes placed on the exterior of each layer harvested the resulting potential difference, and the bilayer MEG maintained a robust performance across a wide range of temperatures and humidity levels.

Beyond vertical stacks, Yao et al. also investigated horizontally arranged bilayer configurations (Fig. 7i), in which laterally adjacent hygroscopic and evaporative regions established an in-plane humidity gradient [102] Thin films comprising glucose oxidase (GOx) and CNF nanoporous structures could be assembled either in a planar arrangement or as vertically stacked layers to form heterogeneous MEG devices. In the planar configuration, both materials underwent water adsorption and desorption at their surfaces. Differences in nanostructure and surface chemistry—particularly the density and type of hygroscopic functional groups—led to a distinct moisture uptake behavior in GOx versus CNF. This mismatch produced different levels of surface charge accumulation, sustaining a voltage difference between the two regions. The resulting charge–concentration gradient drove electron depletion on one side and electron enrichment on the other, generating a measurable electrical output; at 60% RH, the planar device achieved a V_oc_ of ~ 350 mV. Conversely, the vertical configuration exploited differences in moisture adsorption kinetics and charge-storage capabilities between the top GOx layer and bottom CNF layer along the thickness direction, thereby establishing a vertical charge–concentration gradient. The extent of charge accumulation within the CNF layer depended on the thickness of the GOx layer; a GOx thickness of 1.2 µm yielded a V_oc_ of ~ 150 mV. This work expanded the accessible MEG geometries and demonstrated that planar moisture diffusion could drive ion transport and voltage generation.

Building on layered architectures, Deng et al. developed a trilayer composite aerogel featuring dual internal gradients ion-concentration and hydrophilicity gradients (Fig. 7j) [94]. By engineering each layer with distinct surface charge and hydrophilic properties, they established a well-defined dual gradient throughout the structure. Specifically, the three layers were designed to provide progressively varying hydrophilicity and ion density within the integrated structure. The bottom layer, which functioned as the hygroscopic component, was an aerogel composed of carboxylated single-walled carbon nanotubes (SWNTs), polyvinyl alcohol (PVA), and CNFs. This layer was strongly hydrophilic and carried a negative surface charge. The middle layer, assembled from SWNTs and a chitosan/CNF composite, exhibited intermediate hydrophilicity and charge characteristics, and served as a transport pathway for water molecules and ions between the top and bottom layers. The top layer, which acted as the evaporation interface, consisted of aminated SWNTs and chitosan, conferring hydrophobicity and a positive surface charge. In this device, an ultrahigh ionic charge gradient (surface potentials ranging from − 30.2 to + 27.6 mV) was coupled with a gradual wettability transition (water contact angles ranging from 32° to 111° across the thickness) to promote directional charge-carrier diffusion while sustaining a continuous water flow. Under optimized environmental conditions, the trilayer aerogel MEG achieved a maximum V_oc_ of 1.45 V, an I_sc_ of 117 µA, a power density of 32.59 mW cm^−2^, and an energy density of 165.23 mWh cm^−2^, demonstrating remarkable performance for a moisture-driven energy device. This design illustrates a clear structure–function principle: the spatial grading of chemical functionality (ion donors) and water affinity can tailor the internal electrochemical environment to maximize the streaming potential. Compared with uniform structures, such multilayer gradients more effectively coordinate ion transport, bringing MEGs closer to practical high-output applications.

(4) Biomimetic structure

Natural transport strategies have inspired new architectures for hydrovoltaic generators [6, 17, 132–137]. In living systems, water molecules often traverse narrow, protein-lined channels in a highly ordered and directional manner (Fig. 7k) [104]. Biomimetic designs aim to emulate biological ion channels—protein pores that selectively permit specific ions to pass while excluding others—to enhance charge separation under moisture gradients. Li et al. developed an MEG based on asymmetrically patterned CNF/GO composite films, inspired by the selective ion-transport behavior of biological channels. This patterned structure, created via surface imprinting during film formation, directed moisture flow through confined channels, thereby increasing the rate and directionality of water diffusion. Under a humidity gradient, this asymmetric architecture promoted proton dissociation from surface functional groups and guided ion migration, enabling continuous electricity generation. Overall, the study demonstrates that biologically inspired nanoscale patterning can optimize ion-transport pathways in cellulose-based MEGs and improve their energy-conversion efficiency.

(5) Leveraging the intrinsic properties of cellulose for MEG

The intrinsic properties of cellulose can be effectively harnessed to enhance the electrochemical performance and impart multifunctional attributes to MEG systems. The abundant hydroxyl groups of cellulose enable synergistic network formation with inorganic and polymer components through robust hydrogen bonding. For example, He et al. integrated MXene into a cellulose nanocrystal (CNC) matrix, utilizing their complementary surface chemistries to develop a high-performance MEG with enhanced ionic conductivity and mechanical resilience (Fig. 7l) [36]. This strategic coupling underscores how the intrinsic chemical profile of cellulose facilitates the assembly of multifunctional hydrovoltaic devices with superior durability. Also, the inherent hygroscopicity of cellulose derivatives facilitates efficient moisture harvesting. For instance, Park et al. utilized a hydroxypropyl cellulose (HPC) mixed with konjac glucomannan (KGM) as water-collecting gel in MEG device (Fig. 7m) [127]. By functioning as a continuous moisture reservoir, the HPC-KGM layer maintained a steady water supply to the active electricity-generating layer under low-humidity conditions. Collectively, these advancements demonstrate that the multifaceted intrinsic properties of cellulose, ranging from its chemical tunability to superior hygroscopicity, are instrumental in engineering high-performance and environmentally resilient MEG systems.

### Cellulose-enabled EEGs

Cellulose-based EEGs generate electricity via evaporation-driven capillary flow within charged pores, which induces a streaming potential. Performance hinges on sustaining a rapid evaporation-wicking cycle and maximizing liquid-surface interactions, and various strategies to enhance these effects have been reported (Table 2). Pure cellulose systems leverage inherent capillary architectures, specifically focusing on the optimization of woven fabric structures and the naturally aligned microchannels found in wood. While these natural frameworks provide effective liquid transport, their electrical output is often limited by low surface charge density and high internal resistance. Consequently, cellulose-composite-based EEGs integrated with conductive fillers or polymers have been developed to enhance charge-carrier concentration and facilitate faster transport kinetics. By combining the superior wicking capability of cellulose with engineered surface chemistry and improved conductivity, these structural and material refinements directly address the requirements for high-output, continuous evaporation-driven energy conversion.

**Table 2:** Summary of cellulose-enabled EEGs

Active material	Structure	Device structure	Voltage (V)	Current density (μA cm^−2^)	Power density (μW cm^−2^)	Electrode	Condition	Mark	References
T-CNF/carboxylmethyl chitosan/PEDOT:Tos	Aerogel	Generation after dropping solution	0.37	–	–	Ag,Cu	KBr solution 34.7 g 100 mL^−1^, 20 °C	Crosslinking	[138]
Carbon black-coated CA microfiber	Cylinder (Microfiber)	Generation after dropping solution	0.3	100 μA	4.58 μW	Alligator Clip	100 μL of 3.3 M CaCl_2_, RH 40%		[139]
Carbon black-coated cotton fabric	Fabric	Generation after dropping solution	0.74	22.5 μA	2.02 μW	Alligator Clip	2.67 M of CaCl_2_	Self-operating	[140]
Fabric cloth	Fabric	Supplying water from beaker filled with electrolyte solution	~ 0.7	3.4 μA	0.154	Cu/Cu	1 mM CuSO_4_	Using of commercial fabric	[31]
PEDOT:PSS-coated cotton	Fabric (Cotton)	Generation after dropping solution	0.8	13 μA cm^−3^	0.45	Alligator Clip	0.4 M aqueous NaCl solution		[141]
MXene/PANI/cotton fabric	Fabric (Cotton)	Generation after dropping solution	0.688	7.55 mA	1.3 mW	Alligator Clip	3 M of NaCl solution		[142]
CNT coated on cotton	Fabric (Cotton)	Generation after dropping solution	0.71	51 μA	280 μW g^−1^	Iron electrode	DI water		[143]
Carbon black-coated cotton fabric	Fabric (Cotton)		0.53	3.91 μA	255.5 nW	Alligator Clip	0.25 mL of water		[144]
CNT/regenerated cellulose composite	Fiber	Fiber with two electrodes on same side	0.16	0.171 μA	0.4 mW cm^−3^	Cu/Cu	1 M NaCl	Fabrication by continuous wet-spinning method and stitching	[38]
CMC paper	Film (Paper)	Generation after dropping solution	0.25	0.01 μA	–	ITO/Au@SS	RH 70%	Using commercial paper	[145]
Metal-sputtered bacterial CNF	Film		0.935	500	404	Pt/Al	50 μL Water		[146]
Regenerated wood hydrogel	Hydrogel	Supplying the water reservoir, placed in a glass Petri dish	0.55	7 μA	1.35	Pt/Pt	RH 40%	Effect between porous structure by regeneration of cellulose and hydrovoltaic performance	[39]
Janus membrane made of functionalized graphene oxide (FGO)-coated deacetylated cellulose acetate (dCA) electrospun nanofiber	Membrane	Supplying the water reservoir, placed in a glass Petri dish	0.316	0.997	–	Cu/Cu	RH 46%	Degradation in soil	[147]
MoS_2_-functionalized filter paper	Paper	Generation after dropping solution	0.25	21.1	5	Alligator Clip	100 μL of DI water		[148]
Carbon black-impregnated cellulose sponge	Sponge	Generation after dropping solution	0.47	477 μA	224.2 μW	Alligator Clip	3.3 M CaCl_2_ solution, RH of 45–50%	Hydrogen production system	[149]
Delignified wood	Wood	Sandwich	1.1	320 μA	6.75	Pt/Pt	2 M NaOH	Using wastewater	[150]
Cellulose/PEDOT:PSS	Wood	Supplying the water reservoir, placed in a glass Petri dish	0.385	11 μA	0.198 μW cm^−3^	Cu/Cu	26.5% NaCl		[151]
Wood sponge soaked in carbon black	Wood	Generation after dropping solution	0.65	1.2 mA	216 μW	Cu/Cu	3 M LiCl		[152]
Citric acid-treated wood (Beech)	Wood	Sandwich	0.08	0.4	0.45 μW	C@PET/C@PET	RH 60%	Using natural wood	[153]
Carboxylated sugarcane	Aerogel	Supplying water from beaker	0.47	8.2 μA	0.0367	Both SS steel	Sea water	Using sugar cane	[154]
Top: CNF@Hexadecyl Trimethyl Ammonium Bromide (CTAB)-MXene layer; Bottom: polytetrafluoroethylene (PTFE)	Membrane	Bilayer, Floating on	0.3438	66.2 mA	0.75 mW	–	RH 30% with sun illumination		[155]
T-CNF/CNT crosslinked with Fe^3+^	Aerogel	Supplying the water reservoir, placed in a glass Petri dish	0.85	10 μA	3.82	Al/Al	NaCl solution	Aerogel in large-scale preparation	[156]
Ti_4_O_7_ nanofiber embedded T-CNF	Fiber	Supplying the water reservoir, placed in a glass Petri dish	0.94	13.7 μA	0.58 μW	C/C	RH 25% with sun illumination		[157]
CNT coated-wood-based ionic hydrogel(polyacrylamide)	Wood film	Bilayer	1.4	0.1 mA	35	C/C-Zn	RH 25–70%	Operation 1000 h in the natural environment, Scale-up by screen-printing	[158]
Citric acid-treated wood	Wood	Sandwich	0.266	4.3 μA	0.041	SS/SS	RH 50%		[159]
Carbon black/cotton	Fiber	Gradient Janus aerogel fiber	0.6	4.5 μA	1.25 μW	C/C	RH 22%		[42]
Carbon foam-coated cotton cloth	Fabric (Cotton)	Sandwich	0.33	14.4 μA	–	Cu/Cu	RH 40–50%		[160]
CNC/PVA/CNT coated on paper	Paper	Sandwich	0.55	60 μA	75.20 μW cm^−3^	Alligator Clip	RH 50–60%		[161]
SWNT@AgNP-coated nanofiber membrane/PVA@PSS hydrogel/LiCl@cotton	Membrane	Trilayer	0.74	621.64 μA	18.72	C/Al	RH 90%		[162]
MXene/PPy	Fabric (Cotton)	Core–shell hetero structure	0.15	2 mA	150 μW	Alligator Clip	RH 50%		[163]
Cellulosic balsa wood	Wood	Sandwich	0.77	148 μA	8.35 μW	C/Cu	1.2 M CaCl_2_ solution		[164]
Cellulose/CNT aerogel fiber	Fiber	Generation after dropping solution	0.51	2.1 μA	8.327 mW cm^−3^				[165]
CNC/MXene-coated cotton fabric	Fabric (Cotton)	Generation after dropping solution	0.707	7.61 μA	4.38 μW cm^−3^	C/C	1 M LiCl solutions		[166]

#### Pure Cellulose-based EEGs

**Fig. 8:**
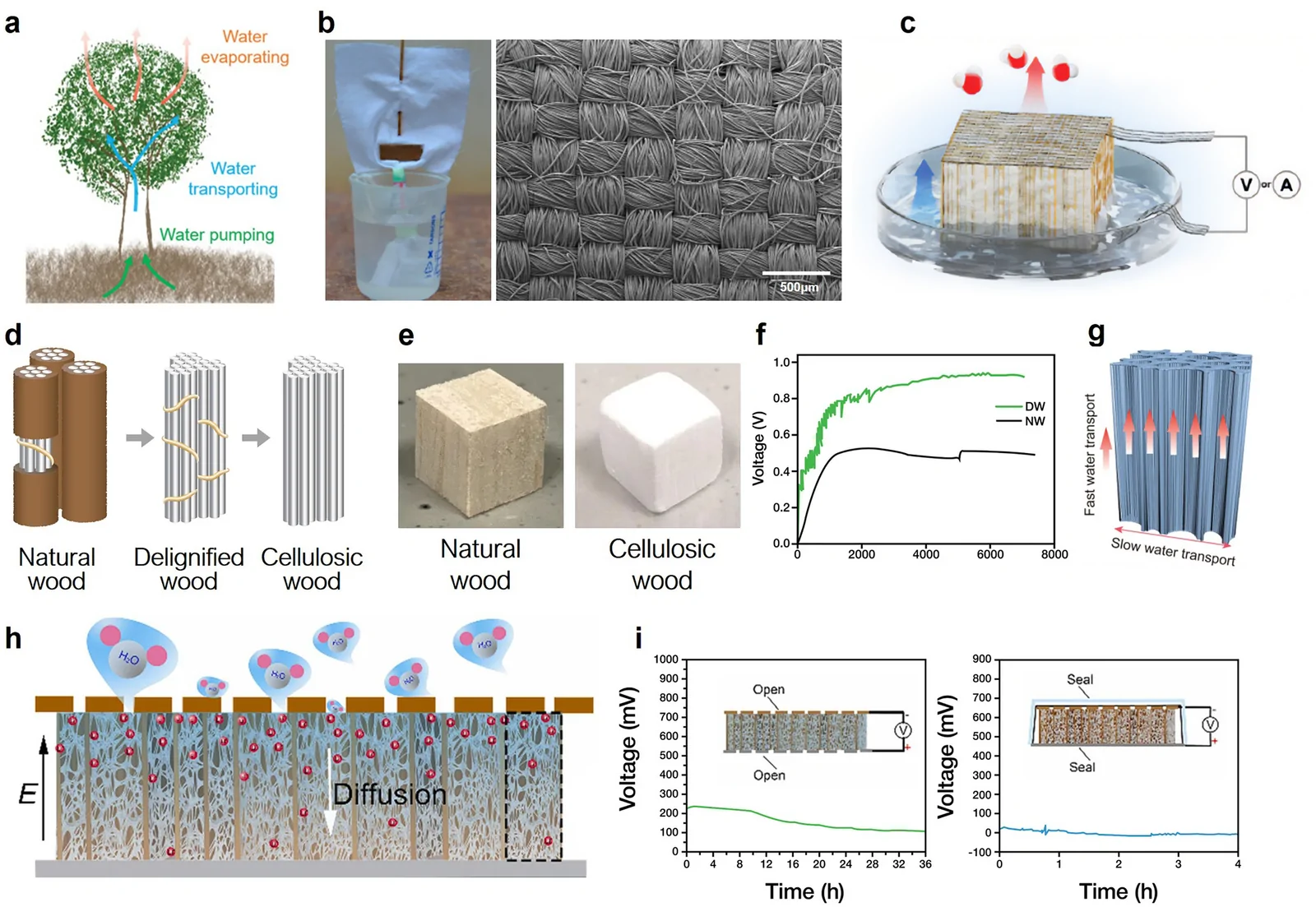
Pure cellulose-based EEGs. **a** Bioinspired water-transport system mimicking plant transpiration to drive EEG. Reproduced with permission [38]. Copyright 2012, Wiley–VCH. **b** Photograph of a fabric-based EEG and SEM image of the fabric. Reproduced with permission [31]. Copyright 2019, American Chemical Society. **c** Schematic of a wood-based EEG. Reproduced with permission [39]. Copyright 2023, Wiley–VCH. **d** Delignification process from natural wood to cellulosic wood. **e** Photograph of natural wood and delignified cellulosic wood. Reproduced with permission [151]. Copyright 2023, Elsevier. **f** Voltage output of EEG with delignified wood and natural wood immersed in a NaOH solution (2 M) with DI-water added on top. **g** Schematic of water transport in wood along parallel and vertical growth directions. **f, g** Reproduced with permission [150]. Copyright 2024, American Chemical Society. **h** Schematic of an ionic wood-based EEG, and **i** its voltage in open and sealed environments. **h, i** Reproduced with permission [168]. Copyright 2022, Elsevier

Cellulose, which is a plant-derived biomaterial, can generate electricity in EEG systems via mechanisms analogous to plant transpiration [167]. In cellulose-based systems, this process is essentially a biomimetic analog of plant transpiration, where water is drawn from the roots to the leaves and generates subtle bioelectric potentials. By mimicking this natural procedure, engineered cellulose architectures can transduce evaporation energy into electrical power. A representative transpiration-inspired design uses a porous cellulose scaffold (often a lightweight nanocellulose aerogel) to emulate capillary water transport in the plant xylem (Fig. 8a) [38]. Water is absorbed at the base, transported through cellulose microchannels, and evaporates from the top surface, creating a continuous ionic current within the fluid. As long as evaporation persists, sustained water and ion transport establish a potential difference between the wet (bottom) and dry (top) ends of the device. Consequently, unlike moisture-powered systems that rely on transient hydration changes, EEGs can deliver a steady DC output provided that a water supply and an evaporation driving force (e.g., heat or dry air) are available. Mechanistically, the voltage originates from an ion-concentration gradient and the associated streaming potential along the cellulose network: as water flows through the charged cellulose pores, it advects counterions, leading to charge separation and the formation of an electric field.

1. Fabric-type cellulose EEGs

Pure cellulose substrates, including cotton fabrics and paper, have been used as simple yet effective EEG platforms. Suman et al. fabricated membranes from fabrics of different grades and types and investigated their relationships with power-generation performance (Fig. 8b) [31]. In a typical configuration, a cellulose fabric functions as an evaporation-induced power generator when one end is immersed in saline water and the other is exposed to air. The woven cotton cloth provides a network of nano- to microscale pores that spontaneously wick water upward via capillary action. As saline water ascends and evaporates from the large surface area of the fabric, dissolved ions are continuously transported through the fibrous network, generating an evaporation-coupled streaming current along the textile. An EDL forms on the charged cellulose fiber surfaces, such that as water flows, excess cations are preferentially carried toward the evaporation side, yielding a measurable voltage between the wet and dry ends. Because this process relies on the intrinsic surface properties of cellulose, it requires no external pumping, enabling a low cost and mechanically robust approach. In field tests, approximately 50 wet garments dried under sunlight (3000 m^2^ total area) produced 10 V over 24 h, which was sufficient to power an LED for more than 1 h. These results underscore the scalability of pure cellulose EEGs, for which an increased evaporation area or device stacking can increase total output. Nevertheless, untreated fabrics are limited by a low surface charge density and poor internal conductivity, which constrain the achievable voltage and power. Despite these limitations, cotton fabrics are inexpensive, widely available, and intrinsically flexible, supporting the practical potential of fabric-type cellulose EEGs.

(2) Wood-type cellulose EEGs

Natural wood provides another compelling cellulose-based architecture for EEGs by leveraging its hierarchical porosity and inherently directional water-transport pathways. Wood is a native composite of aligned cellulose fibers (together with lignin and hemicellulose) organized into channels corresponding to xylem vessels, which naturally conduct water upward in trees. This anisotropic, tube-like microstructure enables directed evaporation-driven flow and is therefore well suited for cellulose-based EEG designs (Fig. 8c) [39]. However, unmodified wood contains substantial noncellulosic components (e.g., hydrophobic, and electrically neutral lignin) that can impede water flux and limit charge generation.

To assess the full potential of wood as a hydrovoltaic generator, chemical pretreatments have been employed to obtain pure or nearly pure cellulose frameworks [169]. An effective strategy is delignification, in which lignin and hemicellulose are removed (often via sodium hydroxide or other oxidative treatments), leaving a white, porous cellulose scaffold. Delignification opens the fine pores of wood and increases its overall porosity and surface charge (Fig. 8d). The appearances of natural wood and delignified cellulosic wood are compared in Fig. 8e [151]. After treatment, the wood becomes markedly lighter in color, indicating the removal of brown lignin, and adopts a more open, sponge-like morphology. The resulting cellulose-rich wood preserves the native aligned microchannel network while exhibiting a substantially higher hydrophilicity and internal surface area. The removal of lignin also exposes additional hydroxyl and carboxyl groups in the cellulose; these groups can ionize in water, increasing the charge density along the channel walls. Tian et al. compared the wood-based EEG performances of natural wood and delignified cellulose wood (Fig. 8f) [150]. Upon addition of deionized (DI) water, delignified wood produced a V_oc_ of approximately 1 V, whereas natural wood generated ~ 0.5 V under identical conditions. This difference is attributable to the lower content of surface functional groups in untreated wood and the limited DI-water infiltration associated with its poorer hydrophilicity. Collectively, these results underscore the importance of chemical pretreatment in wood-based EEG systems. Water transport in wood is also highly anisotropic (Fig. 8g); the aligned cellulose microfibrils enable efficient capillary rise along the longitudinal direction, and the charge-rich channel walls promote a larger ionic imbalance between the bottom (wet) and top (dry) electrodes. Accordingly, the voltage output is orientation-dependent; higher voltages are obtained along the grain (growth direction) than across the grain because a continuous water column is maintained primarily within the longitudinal channels. Capillary-driven flow is significantly faster parallel to fiber growth than transverse to it, and this structural directionality directly influences the power output.

Another strategy to improve wood-based EEG performance is to incorporate ionic or hygroscopic compounds into cellulose, creating “ionic wood” or cellulose-based ion conductors. Liu et al. developed a cellulose EEG based on ionic wood that harvested ambient moisture using a nanostructured, ion-loaded wood scaffold (Fig. 8h) [168]. In this approach, natural wood is treated (e.g., with LiCl or other electrolyte solutions) to form continuous networks of salt nanocrystals along the inner surfaces of the cellulose microchannels. These salt networks strongly bind water and provide a readily available ion reservoir. Because the ions are integrated into the scaffold, the electrical output can be sustained despite external fluctuations. Consequently, ionic wood can generate a moisture-driven current even in ambient air without immersion in liquid water. Under typical indoor humidity, such devices have been reported to produce a stable voltage on the order of a few hundred millivolts (up to ~ 750 mV), along with a relatively high current (~ 712 μA), effectively functioning as a self-powered battery charged by moisture. Nevertheless, continuous evaporation (or water uptake by the hygroscopic medium) is required to drive ion motion. When the system is isolated from ambient evaporation, it eventually reaches equilibrium, and the electricity generation ceases. Figure 8i tracks the long-term voltage of an ionic wood generator under open versus sealed conditions. In open air, where moisture can continuously leave the wood, the device maintains a steady voltage. In contrast, in a sealed chamber, the voltage gradually decays to zero as ion transport diminishes and the system equilibrates. This experiment confirms that evaporation serves as the driving force of the hydrovoltaic cycle by sustaining ion transport and renewing charge separation. Continuous water evaporation is therefore essential to maintain the output.

In summary, pristine cellulose materials can directly convert evaporation into electricity through capillary-driven water transport and an intrinsic surface charge; however, chemical and structural modifications can markedly enhance the EEG performance. Removing nonessential components (e.g., lignin) or incorporating functional additives can increase water uptake, tune ion selectivity, and improve electrical conductivity, thereby increasing the generated voltage and current. Examples ranging from simple cotton fabrics to engineered wood aerogels collectively illustrate how both natural and modified cellulose architectures can function as effective hydrovoltaic generators. These systems draw inspiration from biological water-transport pathways, while improving the performance through materials design—for example, by increasing pore volume, introducing additional charged sites, or embedding conductive networks to maximize the streaming potentials. The result is a class of sustainable devices that harvest electricity from evaporating water. Beyond reinforcing fundamental understanding of ion–fluid interactions in biopolymers, evaporation-driven generation also supports the development of renewable energy technologies, including self-powered sensors and off-grid power sources that operate using only ambient water and heat.

#### Cellulose Composite-based EEGs

Although pristine or chemically modified cellulose structures have demonstrated promising evaporation-driven energy generation, their relatively low conductivity and limited control over ion selectivity often constrain the achievable output. Composite design strategies offer a practical route to address these limitations by integrating conductive nanomaterials, ion-selective interfaces, and scalable fabrication approaches within a cellulose matrix. This section summarizes recent advances in cellulose-based EEGs that use hybrid compositions to improve performance, durability, and functional versatility.

1. Integration of conductive fillers

**Fig. 9:**
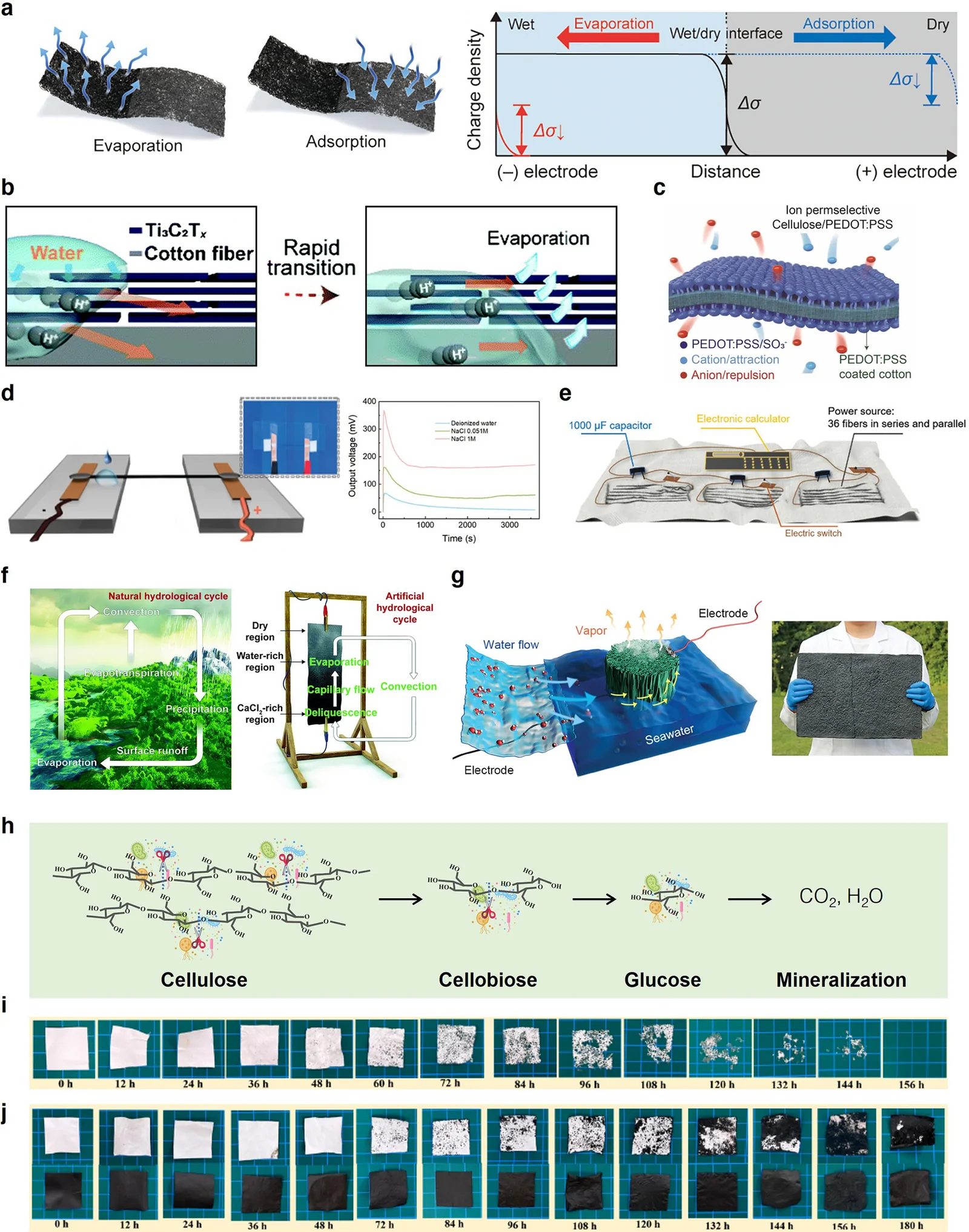
Cellulose composite-based EEGs. **a** Schematic of a carbon black-coated fabric-based EEG under evaporation and adsorption, and analysis of decreased V_oc_ values during these processes. Reproduced with permission [144]. Copyright 2019, American Chemical Society. **b** Schematic of two hydraulic-flow mechanisms through Ti_3_C_2_T_x_ nanosheet layers. Rapid capillary-driven wicking (left) and relatively slow evaporation-induced diffusion (right). Reproduced with permission [142]. Copyright 2022, Royal Society of Chemistry. **c** Schematic of an ion-permselective PEDOT:PSS-coated cotton-based EEG system. Reproduced with permission [141]. Copyright 2022, Elsevier. **d** Schematic of a fiber-structured EEG composed of a cellulose/CNT composite and the voltage of cellulose/CNT composite fibers at different NaCl concentrations; **e** Fabric woven from cellulose/CNT composite fibers. **d, e** Reproduced with permission [38]. Copyright 2022, Wiley–VCH. **f** Schematic of self-operating EEG system using a carbon black-coated cotton fabric. Reproduced with permission [140]. Copyright 2020, Royal Society of Chemistry. **g** CNF/CNT composite aerogel-based EEG and a photograph of a large-scale aerogel. Reproduced with permission [156]. Copyright 2024, Wiley–VCH. **h** Schematic illustration of the biodegradation cycle of cellulose. **i** Photographs of the cellulose matrix at different stages of degradation during the soil burial test. **j** Photographs of Janus-structured cellulose/GO composites at different stages of degradation during the soil burial test. **h**–**j** Reproduced with permission [147]. Copyright 2023, American Chemical Society

Embedding conductive nanomaterials within cellulose frameworks is a well-established strategy to facilitate charge collection and increase streaming-current output in EEGs. Representative fillers include MXenes, metal oxides, and carbon-based materials such as GO, carbon black, and CNTs. Kim et al. addressed the intrinsic limitations of cellulose by coating a hydrophilic cotton fabric with conductive carbon black (Fig. 9a) [144]. The carbon-coated fibers formed an interconnected conductive network that promoted electron transport and provided a high surface area for EDL formation. From a manufacturing perspective, carbon black coatings offer significant advantages because it enables scalable, low-cost fabrication via simple dip-coating. During operation, water evaporation through the coated fabric drives ion transport along the cellulose channels; the conductive carbon network enables rapid charge redistribution, thereby increasing current output. In carbon-black-coated EEGs, V_oc_ is typically high immediately after wetting, then gradually decreases as water evaporates and is retained by the hygroscopic matrix. This trend is consistent with the two-stage generation process of the device: an initial surge upon wetting followed by a slower decay as moisture is depleted. Notably, the peak output of carbon-infused cellulose substantially exceeds that of untreated fabric, highlighting how conductive fillers can reduce internal resistance and enable the so-called pseudostreaming current along the conductive network.

2D nanomaterials, such as MXene (Ti_3_C_2_T_x_), have been incorporated as conductive fillers to fabricate high-performance cellulose composites [170–174]. MXene nanosheets offer metal-like conductivity and unique water-transport pathways, owing to their layered structures. In Ti_3_C_2_T_x_–cellulose EEGs, water permeation proceeds via two sequential mechanisms (Fig. 9b) [142]. First, rapid capillary wicking transports water through micrometer-scale pores and channels in the composite, quickly wetting the device. This is followed by the slower diffusion of water molecules through the interlayers of the stacked MXene sheets, driven by continuous surface evaporation. This two-stage hydraulic transport—fast wicking followed by slow, diffusion-limited interlayer migration—helps sustain moisture supply to the evaporation front over extended periods. In parallel, the highly conductive MXene network facilitates efficient electron transport, enabling the streaming ions to generate higher voltages and currents across the device. By leveraging the layered morphology and high conductivity of MXenes, these composite generators deliver enhanced and more sustained outputs. Accordingly, MXene-integrated cellulose EEGs have been reported to achieve higher power densities and, under optimized conditions, approach watt-scale power generation, highlighting the value of conductive-filler integration for advancing hydrovoltaic devices toward practical operation.

(2) Integration of conductive polymers

Beyond inorganic fillers, incorporating conductive polymers into cellulose EEGs is a widely adopted strategy because of their excellent compatibility with hydrophilic networks, dispersibility, and mechanical flexibility. A representative example is PEDOT:PSS, which can be conformally deposited onto cellulose fibers to create an electronically conductive and ionically functionalized surface (Fig. 9c) [141]. PEDOT:PSS confers electronic conductivity to otherwise insulating cellulose, while the PSS component (a sulfonate-bearing polyelectrolyte) introduces a high density of immobile negative charges on the fiber surfaces. When a PEDOT:PSS-coated cotton fabric is used in an evaporation-driven generator, the coating functions as an ion-selective interface: the negatively charged sulfonate groups promote cation transport while suppressing anion migration. This ion-permselective behavior helps sustain charge separation between the wet and dry ends, thereby increasing the streaming potential relative to uncoated cellulose. Simultaneously, the PEDOT-rich backbone provides a continuous electronic percolation pathway, improving charge collection and transport. Collectively, these effects yield a substantial performance enhancement, with PEDOT:PSS-coated cellulose EEGs producing higher voltage and current outputs than bare cellulose. For example, coated cotton devices can sustain outputs on the order of hundreds of millivolts during continuous operation, whereas uncoated cotton typically decays more rapidly to lower voltages. The conductive-polymer layer can also improve stability and durability by adhering strongly to the fibers, mitigating structural degradation, and maintaining conductivity during swelling and drying cycles. Thus, conductive-polymer integration not only increases immediate power generation through improved surface charge and electronic transport, but also enhances the reliability and application readiness of cellulose EEGs for wearable and textile-based power sources.

(3) Structural design strategies

Beyond chemical modification, structural design provides additional routes for enhancing EEG performance and broadening their applicability. One notable innovation is the fabrication of fiber-type cellulose EEGs, in which the active material is processed into thin, flexible fibers or yarns. Fu et al. developed composite fibers by integrating regenerated cellulose with CNTs, yielding a fiber-type cellulose EEG (Fig. 9d) [38]. These regenerated cellulose/CNT fibers spontaneously wick water along their length and evaporate it from their surface, thereby generating an axial streaming current. Even a single fiber a few hundred micrometers in diameter can produce a measurable voltage when one end is wetted with saline, and the other end is exposed to air. The electrical output of such a fiber can be tuned by controlling the electrolyte conditions. For example, increasing the saline concentration increases the density of charge carriers and enhances the voltage output, typically up to a saturation plateau. The fiber form factor is also advantageous for integration: it is mechanically robust and can be bent or woven into fabrics, enabling wearable and deformable EEG formats. In their demonstration, a bundle of 108 individual cellulose/CNT fibers was woven into a cloth-like panel (Fig. 9e). This multifiber assembly functioned as a scaled generator, with each fiber contributing to the overall output. By connecting fibers in parallel to increase current or in series to increase voltage, the woven device delivered higher power than a single fiber. This result highlights the scalability of the fiber strategy: larger textiles can be produced by adding more fiber threads without altering the underlying evaporation-driven mechanism. Accordingly, fiber- and fabric-based EEGs support practical implementations, including self-powered smart garments and curtains that produce electricity, as well as modular panels for large-area energy harvesting.

Structural design can also be used to control electrical output performance and operating mode. A particularly promising direction is the development of self-operating EEG systems capable of continuous operation without manual water replenishment. A key limitation of evaporation-driven generators is the gradual drying in the absence of periodic water supply. To address this challenge, Kim et al. integrated an artificial hydrological cycle into a carbon-coated cellulose EEG to establish a closed-loop moisture supply (Fig. 9f) [140]. In this device, a carbon-black-coated cotton fabric served as the power-generating element, while a hygroscopic salt (calcium chloride) functioned as an internal water recirculator. During operation, the water evaporating from the wet fabric was captured by CaCl_2_ placed nearby (or incorporated into a reservoir adjacent to the fabric). Deliquescent CaCl_2_ absorbed water vapor and subsequently released it—condensing and dripping back into the fabric—analogous to rainfall replenishing terrestrial surfaces in the natural water cycle. This closed-loop moisture exchange allowed the device to sustain an asymmetric wet state over extended periods. In addition, CaCl_2_ contributed directly to device performance: dissociated Ca^2+^ ions entered the cellulose/carbon network and enhanced the ionic transport, while the saline environment helped stabilize the electrical resistance of the fabric. The analogy to the natural water cycle highlights how a deliquescent medium can be leveraged to enable autonomous, long-term operation. Such self-operating composite systems substantially improve the practical utility of hydrovoltaic generators and advance their prospects for real-world deployment, where maintenance-free, continuous power generation is required.

Research efforts are also focused on improving the practicality and manufacturability of cellulose-based EEGs through materials and process optimization. Xu et al. developed floating and scalable EEG designs that harvest energy from evaporation over abundant water sources using an ambient-dried CNF/CNT composite aerogel as the floating generator (Fig. 9g) [156]. In this design, the cellulose-based generator floats on the surface of a water body (e.g., a lake or reservoir), continuously drawing water from below and releasing vapor into the air above. Owing to its ultralight, porous architecture, the CNT-infused CNF aerogel is buoyant and remains partially submerged while keeping its upper surface exposed for evaporation. The aerogel’s porosity and capillarity support continuous wicking of water from the submerged side to the evaporating surface, thereby sustaining the evaporation-driven flow cycle as long as the device remains on the water. The percolating CNT network endows the aerogel with sufficient electrical conductivity to collect and transport charges over macroscopic distances. Notably, the composite can be produced as large monoliths without specialized equipment, and the use of ambient drying (rather than freeze-drying) supports cost-effective and scalable fabrication. Accordingly, a large CNF/CNT aerogel sheet with lateral dimensions on the order of tens of centimeters was fabricated to generate electricity from natural evaporation. This floating, large-scale EEG concept leveraged an effectively limitless water reservoir and could increase total power output simply by expanding device area. These results suggest the feasibility of deploying arrays of lightweight cellulose composite aerogels on open water as distributed renewable power generators.

(4) Biodegradable cellulose EEG

The sustainability of cellulose-enabled HEG devices is significantly bolstered by their potential for end-of-life biodegradation, a feature that distinguishes them from conventional inorganic-based devices. Enzymes and microbiota in soil hydrolyze cellulose chains into oligoglucans and cellobiose, which are further broken down into glucose. (Fig. 9h) These molecules are ultimately mineralized into CO_2_ and H_2_O, completing the natural composting process. For instance, Wang et al. developed cellulose-composite-based EEG using a Janus membrane based on functionalized graphene oxide (GO) and deacetylated cellulose acetate (CA) nanofibers. (Fig. 9i) [147]. To substantiate the environmental benefits of the device, they conducted a soil burial test to examine the biodegradation behavior of the composite. While the hydrophilic deacetylated CA matrix demonstrated a clear physical disintegration over time, the carbon-based components and other non-degradable fillers incorporated into the matrix did not decompose naturally (Fig. 8j). If buried directly in soil, these persistent materials may lead to environmental accumulation and potential toxicity, underscoring the necessity for effective separation or recovery strategies for non-degradable fillers. Furthermore, because most current studies, including this example, rely primarily on photographic evidence to monitor physical breakdown, a more scientifically rigorous approach is required. Future evaluations should include quantitative analyses, such as measuring the CO_2_ evolution rate during the biodegradation process, to precisely determine the degree and rate of mineralization.

In summary, innovations in materials and structural design have substantially broadened the functionality and application scope of cellulose-based hydrovoltaic systems. These cellulose composites not only deliver improved performance (higher voltages and currents, and more sustained operation) but also enable practical device formats, including wearable power textiles, autonomous environmental energy harvesters, and large-area power pads operating on water surfaces.

### Cellulose-enabled OEGs

Strategies for enhancing the performance of cellulose-based OEGs can be broadly categorized into three approaches: (1) chemical functionalization or structural regulation of neat cellulose, (2) fabrication of cellulose-based composites with other functional materials, and (3) optimization of device architecture (Table 3). Accordingly, this section is organized into three subsections, each detailing the corresponding strategy.

**Table 3:** Summary of cellulose-enabled OEGs

Active material	Testing condition	Voltage (V)	Current (μA)	Power density (W m^−2^)	Maximum energy-conversion efficiency	Long-term operation	t^+^	References
GO/CNF assembled membrane	0.5 M/0.01 M KCl	0.11 V	180 A m^−2^	4.19	30%	–	0.8	[34]
Oppositely charged aligned BC biofilm	0.5 M/0.01 M NaCl	0.16	14.5 μA	0.58 μW	–	15 d	–	[35]
negatively charged carboxymethyl BC membranes	0.5 M–0.01 M NaCl	0.1175	12 A m^−2^	2.25	–	6 mo	0.974	[175]
positively charged chitosan quaternary ammonium BC membranes	0.5 M/0.01 M NaCl	0.085	6 A m^−2^	0.42	–	6 mo	0.079	[175]
graphitic carbon nitride/CNF membrane	0.5 M/0.01 M KCl	–	/	0.15	19.65%	30 d	–	[176]
Layered cellulose/polyaniline (PANI) membrane	0.5 M/0.01 M NaCl	0.18	225 A m^−2^	11.7	44.9%	16 d	0.974	[41]
CNC/PVA/GO membrane	0.5 M/0.01 M KCl	0.045	2 µA	6.5	–	25 d	–	[177]
AAM/BC DN hydrogels	0.5 M/0.01 M KCl	0.056	1.6 µA	7.63	–	10 d	–	[178]
BC nanofiber/2D BN nanosheet composite membrane	0.5 M/0.01 M KCl	~ 0.14	119 A m^−2^	4.59	~ 38%	7 wk	0.963	[179]
BC nanofiber/2D BN nanosheet composite membrane	0.5 M/0.01 M KCl; 50 K temperature gradient	0.165	241 A m^−2^	10	–	–	–	[179]
Aligned cellulose/CNT nanofluidic fibers	0.5 M/0.01 M KCl	~ 0.08	~ 38 A m^−2^	3.19	36.8%	43 d	0.93	[180]
Stacked montmorillonite nanosheets/intercalated cellulose nanofibers	0.5 M/0.01 M NaCl	0.108	17.85 µA	8.61	21%	> 30 d	0.92	[181]
COF-LZU1/CNT–CNF nanofluidic hybrid membranes	0.5 M/0.01 M NaCl	0.02704	1.28 µA	4.26	–	–	–	[182]
Fe_3_O_4_ embedded amino-functionalized cellulose membrane/poly-l-lysine modified PET	0.5 M/0.01 M NaCl	0.0452	18.7µA	4.9	5.32%	–	–	[183]
ultrasmall MoS_2_ nanosheet/CNF composite membrane	0.5 M/0.01 M NaCl	~ 0.11	~ 80 A m^−2^	2.3	–	10 d	–	[184]
BC/MXene membrane	0.5 M/0.01 M NaCl	0.1745	121 A m^−2^	5.3	–	–	0.954	[185]
MXene/CNF membranes	100 mM/0.1 mM KCl	0.08	0.98 µA	0.15	20%	10 d	0.75	[186]
Alternatingly stacked TEMPO-oxidized CNFs and MXene membranes	0.5 M/0.01 M KCl	0.154	6 µA	6.96	42%	14 d	0.8	[187]
CNC/PVA@UiO-66-(COOH)_2_ composite membrane	0.5 M/0.01 M KCl	–	–	5.1	17.2%	19 d	0.5	[188]
Mn-based MOF/TEMPO-oxidized CNFs hybrid membrane	0.5 M/0.01 M KCl	~ 0.085	~ 0.03 µA	1.87	36%	12 d	0.93	[189]
TEMPO-oxidized Bacterial CNFs/GO fibers	0.05 M/0.001 M KCl	0.0918	15.3 A m^−2^	0.35	38%	15 d	0.94	[190]
Metallic-phase WS2/CNFs composite membranes	0.5/0.01 M NaCl	0.05581	4.37 µA	1.99	–	–	–	[191]
PNIPAM brushes-grafted sulfated nanocellulose membrane	0.5 M/0.01 M KCl; 50 °C	0.125	10.25 µA	10.1	–	16 d	–	[192]
negatively and positively charged BC membrane pairs	0.5/0.01 M NaCl	–	–	44.1 mW m^−2^	–	–	–	[193]
aligned CNF membrane	0.5 M/0.01 M KCl	–	–	658 mW m^−2^	40%	–	0.948	[194]
Molecular self-assembled cellulose	0.5 M/0.01 M KCl	–	–	2.27	–	100 d	0.97	[195]
High-aligned oppositely charged nanocellulose/MXene aerogel membranes	0.5 M/0.01 M NaCl	–	–	8.87	33.6%	168 h	0.91	[196]
oppositely charged CNC intercalating GO membranes	0.5 M/0.01 M KCl	0.176	24 µA	4.73	29%	7 d	0.89	[197]
negatively charged BC/GO and positively charged BC/layered double hydroxide membrane pairs	0.5 M/0.01 M NaCl	–	–	0.7	48%	60 d	1	[198]

#### Pure Cellulose-based OEGs

Native cellulose typically lacks the charge density, structural robustness, and nanofluidic control required for high-performance OEGs; therefore, a range of modification strategies, spanning from surface chemical grafting to hierarchical structural reconstruction, have been developed to improve the ion-selective and nanofluidic properties of cellulose-based OEGs.

The most straightforward cellulose modification route involves covalent grafting of ionic groups onto the hydroxyl-rich cellulose backbone. Zhang et al. used a phase-inversion process to fabricate a positively charged cellulose membrane (PPC) via etherification with 2,3-epoxypropyltrimethylammonium chloride (EPTAC) [199]. This functionalization endowed the membrane with high cationic charge density (zeta potential up to + 14 mV) and produced uniform nanochannels (~ 7.2 nm), thereby improving anion selectivity and lowering internal resistance (11 kΩ). Under a 50-fold salinity gradient, the PPC membrane delivered a power density of 2.2 W m⁻^2^ and maintained structural integrity for 300 days across a broad pH range (3–12). These results underscore that ionic functionalization can effectively modulate Donnan potentials and Debye screening effects, which are central to selective ion transport.

**Fig. 10:**
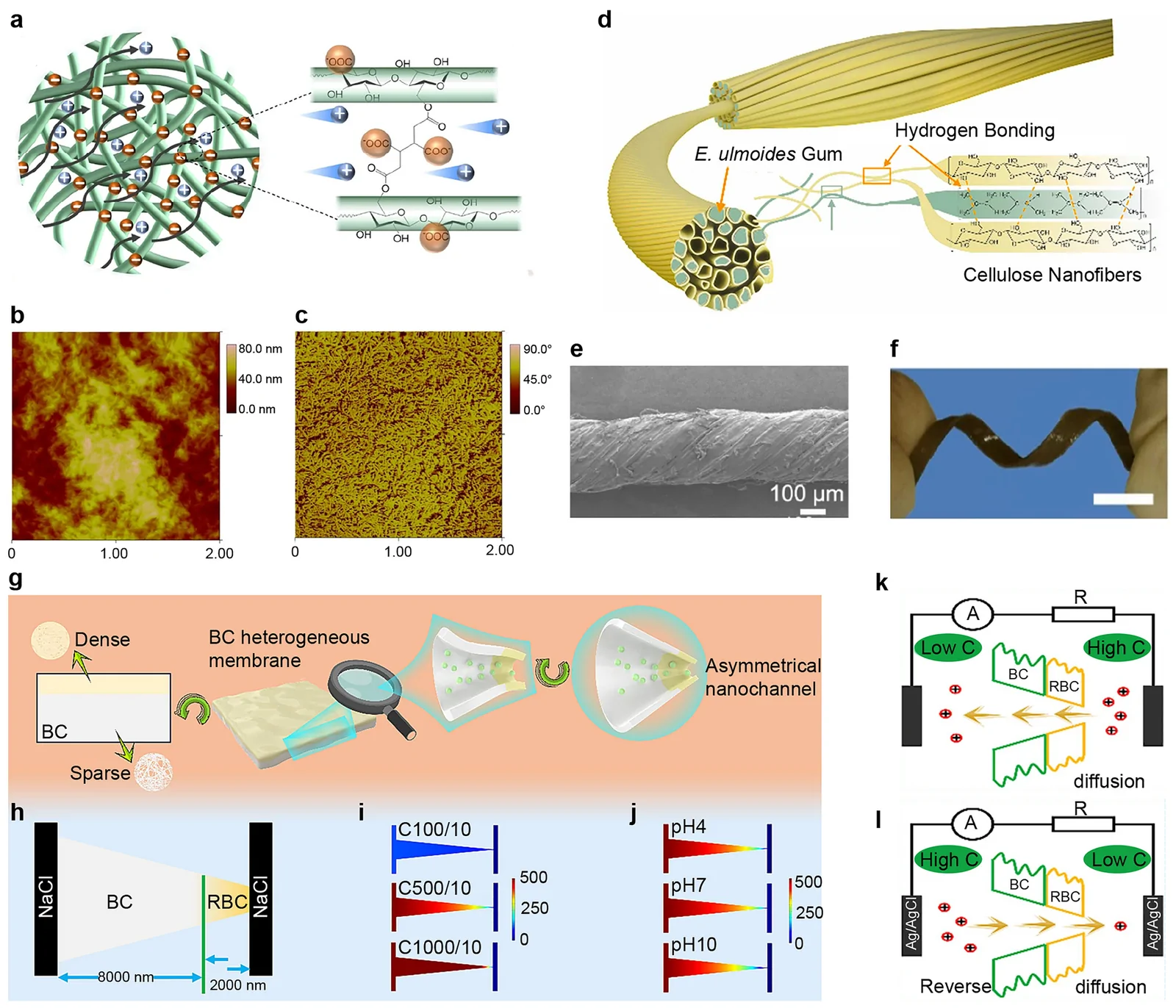
Pure cellulose-based OEGs. **a** Schematic of a nanofibrillar cellulose network crosslinked to form ion-selective pathways that enable efficient cation transport. **b, c** AFM phase and topography images of a BTCA (20 wt%) crosslinked nanocellulose membrane surface, showing the crosslinking-induced morphological evolution. Reproduced with permission [200]. Copyright 2022, American Chemical Society. **d** Schematic of the fabrication process of a bark-derived ionic cable. **e** SEM image of the ionic cable, showing its densely packed structure and highly aligned cellulose fibers. **f** Optical images of the flexible bark film after alkalinization; the resulting ionic cable maintains mechanical integrity under substantial bending. **d**–**f** Reproduced with permission [201]. Copyright 2024, Elsevier. **g** Schematic of an asymmetric nanochannel structure designed to achieve directional ion transport. **h** Illustration of the Poisson–Nernst–Planck (PNP) model used to describe ion migration within nanochannels. **i** Simulated output performance under concentration gradients of 10 ×, 50 ×, and 100 × at neutral pH (pH = 7). **j** Simulated output at a fixed 50-fold concentration gradient across a range of pH values, demonstrating pH sensitivity. **k, l** Schematics of forward and reverse ion diffusion in BC-based heterogeneous membranes during osmotic energy-harvesting tests. **g**–**l** Reproduced with permission [202]. Copyright 2024, Elsevier

Beyond simple surface grafting, chemical crosslinking can reinforce structural integrity while simultaneously regulating charge density and channel dimensions. Yang et al. developed 1,2,3,4-butanetetracarboxylic acid (BTCA)-crosslinked nanocellulose membranes by esterifying CNFs with BTCA (Fig. 10a–c) [200]. By varying the BTCA content, they synergistically tuned both the membrane’s negative charge density and nanochannel size. The optimal formulation (20 wt% BTCA) produced overlapping Debye layers at low salt concentrations (~ 1 mM), yielding high ion selectivity (cation transference number, t⁺ ≈ 0.81) and a power density of up to 8.87 mW m⁻^2^. Crosslinking also markedly improved water stability, enabling 12 h of continuous operation with no structural degradation. This dual regulation of chemical functionality and network topology represents an important advance in cellulose nanofluidics.

Structural densification and hierarchical pore engineering are essential for promoting directional ion transport and reducing tortuosity. Song et al. reported a bark-derived ionic cable fabricated by delignifying the bark of *E. ulmoides* and densifying its lignocellulosic structure via wet twisting (Fig. 10d–f) [201]. This process removed hemicellulose/lignin and increased hydroxyl availability, thereby increasing the zeta potential (− 31.17 mV) and ionic conductivity (3.36 × 10⁻^3^ S cm⁻^1^). The aligned CNFs and natural adhesive EUG provided high mechanical strength (~ 31.5 MPa) and dimensional stability. Under a 1000-fold salinity gradient, the cable delivered a power density of 0.51 W m⁻^2^, which increased to 1.45 W m⁻^2^ under simulated seawater/river gradients. This study underscored the value of top-down optimization of biomass architecture, leveraging both natural fiber alignment and composite binding to form robust nanofluidic conduits.

At the highest level of complexity, the monocomponent heterostructure design enables asymmetric ion transport and directional selectivity without multilayer composites. Zhang et al. introduced asymmetric nanoconfinement through ionic liquid (IL)-induced unidirectional regeneration of BC (Fig. 10g–l) [202]. The resulting monocomponent heterogeneous membranes exhibited a gradient in fiber density across the two sides, forming asymmetric nanochannels that favored unidirectional ion diffusion. Under a 100-fold salinity gradient, the membrane achieved a V_oc_ of up to 40 mV and a power density of 0.70 W m⁻^2^, with enhanced performance under alkaline conditions (up to 0.60 W m⁻^2^ at pH 10). By avoiding interfacial defects typical of multimaterial heterostructures, this approach enables intrinsic directional selectivity. This strategy opens new avenues for single-material heterostructuring in osmotic applications.

Collectively, these studies reveal a clear trend: cellulose modification for osmotic energy conversion has progressed from straightforward ionic surface grafting toward increasingly precise control of network architecture and nanofluidic asymmetry. Each added level of structural complexity improves distinct performance metrics—including charge density, conductivity, mechanical robustness, and selectivity—highlighting the broad design space for cellulose-based membrane engineering.

#### Cellulose Composite-based OEGs

Combining CNFs with functional materials provides a versatile and scalable route to engineer nanofluidic channels with enhanced ionic conductivity, selectivity, and structural robustness. In this section, we categorize recent advances in cellulose-based composites according to the incorporated components: inorganic fillers, organic/polymeric systems, MOFs, COFs, and multifunctional hybrids with stimuli-responsive behavior. This framework facilitates a systematic comparison of how specific material combinations modulate the physicochemical properties of nanofluidic membranes and optimize their performance in OEGs.

1. Incorporation of inorganic fillers

Various inorganic nanomaterials have been incorporated into cellulose-based hybrid systems. Inorganic fillers—particularly those with high surface charge density, layered morphologies, or intrinsically ion-selective transport properties—can substantially improve both the electrochemical performance and mechanical stability of cellulose-based OEGs. In this section, we categorize recent advances into three representative groups according to inorganic filler type: carbon-based fillers, conventional layered/semiconductive inorganics, and emerging two-dimensional MXenes.

**Fig. 11:**
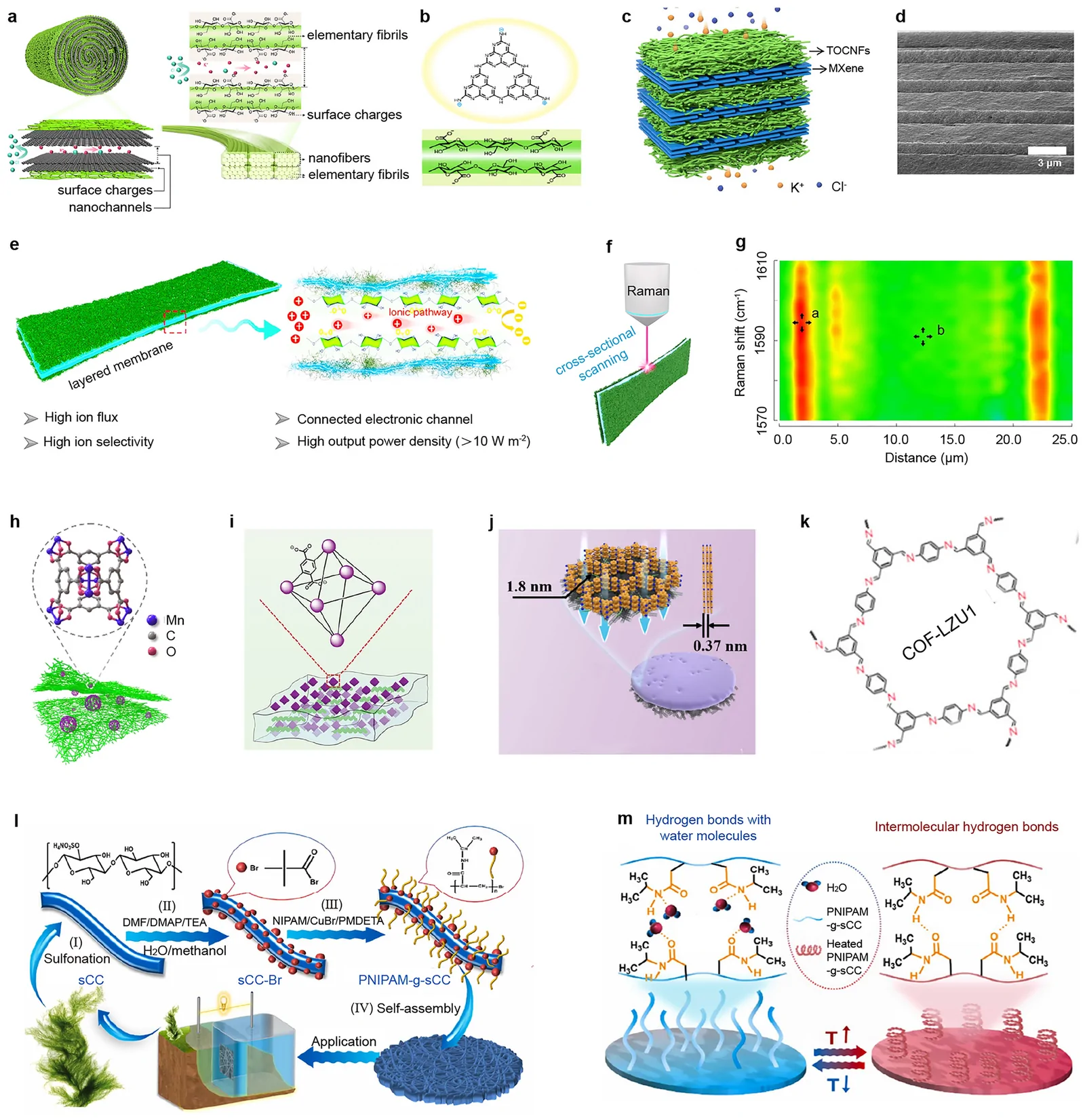
Cellulose composite-based OEGs. **a** Schematic of 2D nanofluidic channels formed by overlapping Debye layers between adjacent GO sheets, enabling selective cation transport. In parallel, elementary fibrils in TEMPO-oxidized bacterial cellulose (TOBC) nanofibers create 1D nanofluidic pathways that confer similar ionic selectivity. Reproduced with permission [190]. Copyright 2021, American Chemical Society. **b** Structural illustration of a graphitic carbon nitride (g-C₃N₄)/CNF composite membrane. Reproduced with permission [176]. Copyright 2022, Elsevier. **c** Schematic representation of multistage heterogeneous nanochannels driving transmembrane ion transport under a concentration gradient. **d** Cross-sectional SEM image of a layered MXene/TOCNFs membrane, showing dense lamellar alignment. **c**–**d** Reproduced with permission [187]. Copyright 2024, Elsevier. **e** Schematic of an aligned layered membrane architecture that achieves both high ion flux and high ion selectivity. **f**,** g** Raman spectral mapping showing the heterogeneous distribution of polyaniline on the membrane surface; **g** presents the corresponding 2D Raman image of polyaniline spatial dispersion. **e**–**g** Reproduced with permission [41]. Copyright 2024, American Chemical Society. **h** Schematic of a hybrid membrane comprising MOFs integrated with TEMPO-cellulose nanofibers (T-CNF). Reproduced with permission [189]. Copyright 2024, Elsevier. **i** Schematic of the structure of a CNC/PVA@UiO-66-(COOH)₂ composite membrane incorporating CNCs and polyvinyl alcohol (PVA) as the polymer matrix. Reproduced with permission [188]. Copyright 2024, Wiley–VCH. **j** Schematic of a COF-LZU1@CNT–CNF nanofluidic hybrid membrane combining COFs, CNTs, and CNFs. **k** Atomic structure model of the COF-LZU1 framework. **j**,** k** Reproduced with permission [182] Copyright 2022, American Chemical Society. **l** Schematic of the fabrication process of the thermoresponsive PNIPAM-g-sCC (poly(N-isopropylacrylamide)-grafted sulfonated cellulose composite) membrane. **m** Schematic of the thermoresponsive gating mechanism of the PNIPAM-g-sCC membrane under varying temperature conditions. **l**,** m** Reproduced with permission [192]. Copyright 2023, Elsevier

Carbon-based fillers, particularly GO and CNTs, have become prominent inorganic additives for improving the osmotic energy-harvesting performance of cellulose-based membranes [34, 177, 180, 190]. GO–cellulose hybrids leverage the high surface area and negative surface charge of GO, together with the structural reinforcement and electrostatic contributions of CNFs, thereby enhancing ion selectivity and lowering transport resistance. For example, GO/CNF membranes showed expanded interlayer spacing (1.32 nm), reduced activation energy (6.58 kJ mol⁻^1^), and a peak power density of 7.2 W m⁻^2^ at 323 K [34]. Similarly, ternary composites comprising GO, PVA, and CNCs had improved lamellar uniformity and mechanical integrity, delivering 3.9 W m⁻^2^ under a 50-fold NaCl gradient [177]. For fibrous architectures, wet-spun GO–TOBC hybrid fibers integrated 1D and 2D transport pathways, producing 0.53 W m⁻^2^ with 38% efficiency; this performance was supported by a high surface charge (ζ = –60.7 mV) (Fig. 11a) [190]. In a more advanced design, regenerated cellulose nanofluidic fibers (RCNFs) incorporating acidified CNTs (up to 40 wt%) formed highly aligned 3–4 nm channels and maintained an ionic conductivity of 0.07 S cm⁻^1^ [180]. These RCNFs sustained a power density of 2.57 W m⁻^2^ over 43 days and retained an underwater mechanical strength of 29 MPa. Collectively, these studies highlight the synergistic roles of nanocarbon fillers in enhancing charge transport and structural durability, enabling high-performance and long-term stable osmotic energy devices.

Cellulose/inorganic semiconductor composites have also been used to improve OEGs, with steady gains in power output, ion selectivity, and structural stability. Early systems incorporating boron nitride with CNFs demonstrated enhanced thermo-osmotic energy harvesting, achieving a power density of ~ 1.2 W m⁻^2^ under a thermal gradient [179]. Further advances included integrating graphitic carbon nitride (g-C₃N₄) into cellulose frameworks to create lamellar nanochannels with a high surface charge density (Fig. 11b) [176, 203, 204]. These membranes maintained an ionic conductivity of 0.009 S cm⁻^1^ and delivered 0.15–0.22 W m⁻^2^ at room temperature and 333 K, respectively, with stable operation over 30 days. To address the limitations of long ion-transport pathways typically found in 2D laminates, composites based on ultrasmall MoS₂ nanosheets (~ 100 nm) were developed [184, 205, 206]. By shortening ion pathways and reducing membrane impedance (48 kΩ), these membranes achieved 2.3 W m⁻^2^ at room temperature, which further increased to 5.5 W m⁻^2^ at 80 °C. A fully natural 2D nanofluidic membrane composed of montmorillonite and CNFs showed significantly improved performance, as interlocking layered structures and coupled space/surface charge effects promoted highly selective cation transport [181]. This membrane achieved a record power density of 8.61 W m⁻^2^ under a 50-fold NaCl gradient and maintained long-term stability (> 30 days), with consistent performance across membranes of up to 700 cm^2^. Collectively, these advances indicate a shift from simple surface charge enhancement toward optimized nanoconfinement and multidimensional architectures, supporting scalable and sustainable osmotic energy-harvesting technologies.

Recent advances in MXene–cellulose composite membranes have highlighted their potential as high-performance platforms for osmotic energy harvesting by integrating ion selectivity, mechanical robustness, and biocompatibility [207]. In a representative design, negatively charged bacterial cellulose (NBC) was interwoven with Ti₃C₂Tx MXene nanosheets to form laminated nanofluidic membranes via vacuum filtration [185]. This hybrid architecture exploited the high zeta potential (− 61 mV) and hydrophilicity of NBC to enhance ion flux and membrane stability, achieving a maximum power density of 5.3 W m⁻^2^ under a 50-fold NaCl gradient. Notably, when a saline gelatin hydrogel was employed as the solid-state electrolyte, the device maintained a power output of 2.58 W m^2^ and showed excellent in vitro and in vivo biocompatibility, underscoring its potential for implantable medical devices. Building on this approach, a mechanically reinforced MXene/CNF membrane was fabricated through sequential alignment and hydrogen bonding between TEMPO-oxidized CNFs and MXene sheets [186]. The resulting lamellar structure exhibited high ionic conductivity (4 × 10⁻^3^ S cm⁻^1^) and tensile strength (171.2 MPa), together with aqueous stability over 10 days. Under a 1000-fold KCl concentration gradient, the composite delivered a power density of 0.15 W m⁻^2^, with minimal performance decay and stable operation across a range of pH values and channel lengths. The CNF-induced expansion of interlayer spacing (up to 1.58 nm) and the increased surface charge (up to − 4.1 mC m⁻^2^) promoted charge-governed ion transport. More recently, a soil-inspired, multistage heterogeneous structure was realized by alternately stacking TOCNF and MXene layers to emulate stratified ion-sieving systems (Fig. 11c, d) [187] In this design, the “soft” TOCNF layer acted as the selective gate, whereas the “hard” MXene layer provided a conductive scaffold, creating hierarchical channels with confined interfacial charges. This configuration delivered a peak power density of 6.96 W m⁻^2^ under 0.5/0.01 M KCl and a record output of 95.13 W m⁻^2^ under hypersaline gradients (5/0.5 M NaCl). Furthermore, the device exhibited cation transference numbers exceeding 0.80 and high mechanical strength (200.5 MPa), while remaining stable in aqueous environments over 2 weeks. Collectively, these results indicate that rational multilayer integration of 1D and 2D materials can synergistically tune ion selectivity and transport resistance, thereby advancing the performance ceiling of membrane-based blue-energy technologies.

(2) Embedded polymeric fillers

While inorganic fillers often provide strong ionic selectivity and mechanical reinforcement, polymeric fillers offer greater chemical tunability, processability, and compatibility with cellulose substrates. By decoupling ionic and electronic transport pathways or constructing charge-regulated 3D networks, these systems can enhance ion flux and structural integrity, enabling high output under complex environmental gradients.

Xie et al. developed an anisotropic layered membrane comprising regenerated cellulose and surface-deposited polyaniline (PANI), wherein highly oriented cellulose nanochannels facilitated ion conduction and an external PANI network enabled efficient electronic transport (Fig. 11e–g) [41]. With a Herman’s orientation factor of 0.81 and markedly reduced internal resistance (only 1/68 that of a blend membrane), this architecture delivered a power density of 11.7 W m⁻^2^ under a 50-fold NaCl gradient and retained over 90% of its performance after 16 days, demonstrating robust structural stability and efficient charge transport.

In a complementary approach, Sun et al. prepared a double-network (DN) hydrogel by photopolymerizing an acrylic acid-based copolymer (AAM) within the microporous matrix of BC [178]. This strategy converted micron-scale pores into interconnected nanochannels (~ 19.2 nm), substantially increasing ion selectivity and surface charge density (from − 1.14 to − 2.59 mC m⁻^2^). The resulting AAM/BC DN hydrogel exhibited strong cation-selective transport and pronounced pH responsiveness, delivering a power density of 7.63 W m⁻^2^ under alkaline conditions (pH 11) and up to 45.5 W m⁻^2^ via acid–base neutralization. Furthermore, it demonstrated excellent applicability under practical conditions, generating 28.4 W m⁻^2^ when harvesting osmotic energy from black liquor wastewater and seawater.

(3) Composites with MOF/COF hybrids

The incorporation of porous crystalline frameworks, such as MOFs and COFs into cellulose-based membranes represents a promising route for enhancing ion transport through synergistic structural and interfacial engineering [182, 188, 189]. These frameworks provide uniform nanochannels, high surface areas, and readily tunable functionality that complement the fibrous and hydrophilic nature of cellulose substrates.

Wang et al. fabricated a CNC/PVA composite membrane containing 27.4 wt% UiO-66-(COOH)₂ via ambient-temperature in situ growth (Fig. 11i) [188]. The membrane featured interconnected 3D nanochannels and a high negative surface charge density, which together promoted selective ion transport. Under a 50-fold KCl gradient, it delivered a peak power density of 5.10 W m⁻^2^, approximately fourfold higher than that of the MOF-free counterpart. The system also demonstrated superior monovalent selectivity (K⁺/Mg^2^⁺ ≈ 16), long-term structural stability (19 days), and robustness under natural water gradients, highlighting the potential of MOF–cellulose hybrids for scalable blue-energy devices. Similarly, Fu et al. incorporated Mn-BTC MOFs into TEMPO-oxidized CNF (T-CNF) to produce flexible hybrid membranes (Fig. 11h) [189]. The MOFs both expanded the effective nanochannel dimensions and enhanced the hydrophilicity, thereby increasing ionic flux. Under a 50-fold KCl gradient, the hybrid membrane achieved a maximum power density of 1.87 W m⁻^2^, approximately five times that of pristine T-CNF. Notably, the ion selectivity corresponded to a cation transference number (t⁺) of 0.93, and the energy-conversion efficiency reached 36%, which are among the highest reported values for CNF-based nanofluidic membranes. This performance was maintained for 12 days, and series-connected devices generated > 1.8 V, sufficient for powering low-power electronics.

To address interfacial transport bottlenecks, Li et al. deposited a COF-LZU1 layer onto CNT/CNF supports to obtain an asymmetric COF–CNT–CNF hybrid membrane (Fig. 11j, k) [182]. The COF layer provided densely packed, aligned ~ 2 nm pores for ion sieving, whereas the CNT–CNF scaffold offered a 3D ionic buffering space. This architecture promoted directional ion migration, lowered the interfacial resistance, and effectively suppressed the concentration polarization. Under real seawater–river water gradients, the hybrid membrane delivered 4.26 W m⁻^2^, outperforming many earlier nanofluidic RED systems, and maintained a stable performance across variations in pH, electrolyte identity, and temperature.

In summary, MOF/COF hybrid membranes leverage hierarchical channel architectures and charge engineering to improve osmotic energy conversion. Future efforts should focus on tailoring framework chemistry and multiscale architecture to further increase selectivity, reduce internal resistance, and improve mechanical durability under practical applications.

(4) Composites coupled with other energy-conversion principles

Beyond passive structural optimization, several cellulose-based composites incorporate active coupling mechanisms such as photothermal, photoelectric, or thermoresponsive effects—to further enhance osmotic energy conversion and enable stimuli-responsive functionalities.

A representative example is the Fe_3_O_4_–RC/PLL system, in which photothermal gating is realized by embedding Fe_3_O_4_ nanoparticles into a regenerated cellulose matrix and integrating it with a PLL-modified PET substrate [183]. Upon solar illumination, the Fe_3_O_4_ induces localized heating, triggering a conformational transition in PLL (α-helix to β-sheet) that modulates nanochannel geometry and enhances ionic rectification (up to 116.76), yielding a power output of 4.9 W m^−2^. Gao et al. developed a WS_2_/CNF membrane that leveraged the high conductivity and photo-responsiveness of metallic-phase WS_2_ [191]. In this membrane, CNFs acted as mechanical reinforcers and space-charge donors, enabling cation-selective transport. Under illumination, the asymmetric charge redistribution in the WS_2_ nanosheets generated a photo-induced voltage, effectively doubling the power density to 1.99 W m^−2^.

Lin et al. developed a PNIPAM-grafted sulfated nanocellulose membrane that exhibited temperature-gated ion transport. Below LCST (~ 37 °C), extended PNIPAM chains impeded ion transport; above LCST, coil-to-globule transitions reopened the nanochannels, increasing conductance and selectivity (Fig. 11l, m) [192]. This reversible gating enabled a tunable energy output, which reached 10.1 W m^−2^ at 50 °C and powered small electronic devices in wearable RED prototypes.

Overall, the wide range of reported power densities for cellulose-based OEGs (≈0.1–95 W m⁻^2^) reflects both intrinsic material properties and experimental conditions. Intrinsically, surface charge density and ζ potential govern ion selectivity and electrostatic coupling; membranes incorporating charged fillers or chemically oxidized celluloses typically enhance counterion transport while suppressing co-ion leakage. Channel confinement is also pivotal: when the pore size approaches the Debye length, overlapping EDLs exclude co-ions and enable unipolar conduction, substantially increasing output. Structurally, well-aligned or layered architectures reduce tortuosity and internal resistance, supporting high ion flux. Externally, differences in concentration gradient, electrolyte composition, temperature, and test configuration further contribute to interstudy variability. Accordingly, systems that combine high surface charge, optimal nanoconfinement (a ≈ λ_D_), and large salinity differentials consistently deliver the highest power densities.

#### Device Architecture Engineering

The integration of oppositely charged membrane pairs has emerged as a highly effective structural strategy for OEGs. Such configurations enable complementary cation and anion transport under salinity gradients, thereby maximizing electrochemical potential and ionic flux. The following section classifies membrane-pair systems and discusses their material compositions, nanochannel structures, ion selectivities, and energy-conversion performances.

1. Naturally aligned BC membrane pairs

Natural BC membranes have been chemically modified to produce positively charged (PBC) and negatively charged (NBC) variants via etherification and TEMPO-mediated oxidation, respectively [193–195]. Zhang et al. showed that wet-drawing could assemble oppositely charged membranes into aligned anisotropic structures (P-ABC and N-ABC), markedly improving ion selectivity and ionic conductivity [35, 208]. The alignment of nanofibrils induced highly ordered channel-like nanopores that promote effective EDL overlap, leading to high cation and anion transference numbers (t^+^ ≈ 0.83 for NBCM and ≈ 0.15 for PBCM). Using such BC membrane pairs, the maximum output power density reached 0.72 W m^−2^, with the stability exceeding 60 days [208]. Tandem stacks comprising up to 20 membrane pairs yielded voltages above 1.8 V, sufficient to power small electronic devices, underscoring the scalability of this approach.

(2) Inorganic-filler composite membranes with charge complementarity

Hybrid membranes that integrate 1D BC nanofibers with 2D inorganic nanosheets (e.g., GO and layered double hydroxides (LDHs)) have been investigated [198]. These systems form lamellar nanofluidic architectures with well-defined subnanometer ion channels. In NBC/NGO and PBC/PLDH composite pairs, charge-complementary ion-selective membranes combine high ionic conductivities with strong selectivities. Under a 50-fold NaCl gradient, this dual-membrane system delivered an output power density of 0.70 W m^−2^, while individual NBC membranes yield up to 4.86 W m^−2^ when tested with Ag/AgCl electrodes. This exceptional performance is attributed to the interplay between the high surface charge density and nanoconfined ion transport.

(3) Structural densification and directional porosity enhancements

To improve the structural order and mechanical integrity, freeze-casting-derived and intercalated membrane architectures have been developed [175, 196, 197]. For example, oppositely charged nanocellulose/MXene aerogels [196] and CNC-intercalated GO membranes [197] exploit directionally aligned porosity and enhanced robustness to support efficient ion transport. Similarly, stretched BC biofilms with increased charge density exhibit higher transmembrane current and voltage outputs than their unstretched counterparts [175]. Although these approaches typically yield moderate output power densities (~ 0.5 W m^−2^), they highlight the importance of structural anisotropy and mechanical reinforcement for practical membrane applications.

In conclusion, the strategic assembly of oppositely charged membrane pairs—based on pristine BC, composite lamellar architectures, or engineered porous frameworks—provides a robust and scalable platform for high-performance osmotic energy harvesting. By balancing the ion selectivity, channel geometry, and structural integrity, these systems offer practical pathways toward sustainable blue-energy generation.

### Cellulose-enabled DEGs

**Fig. 12:**
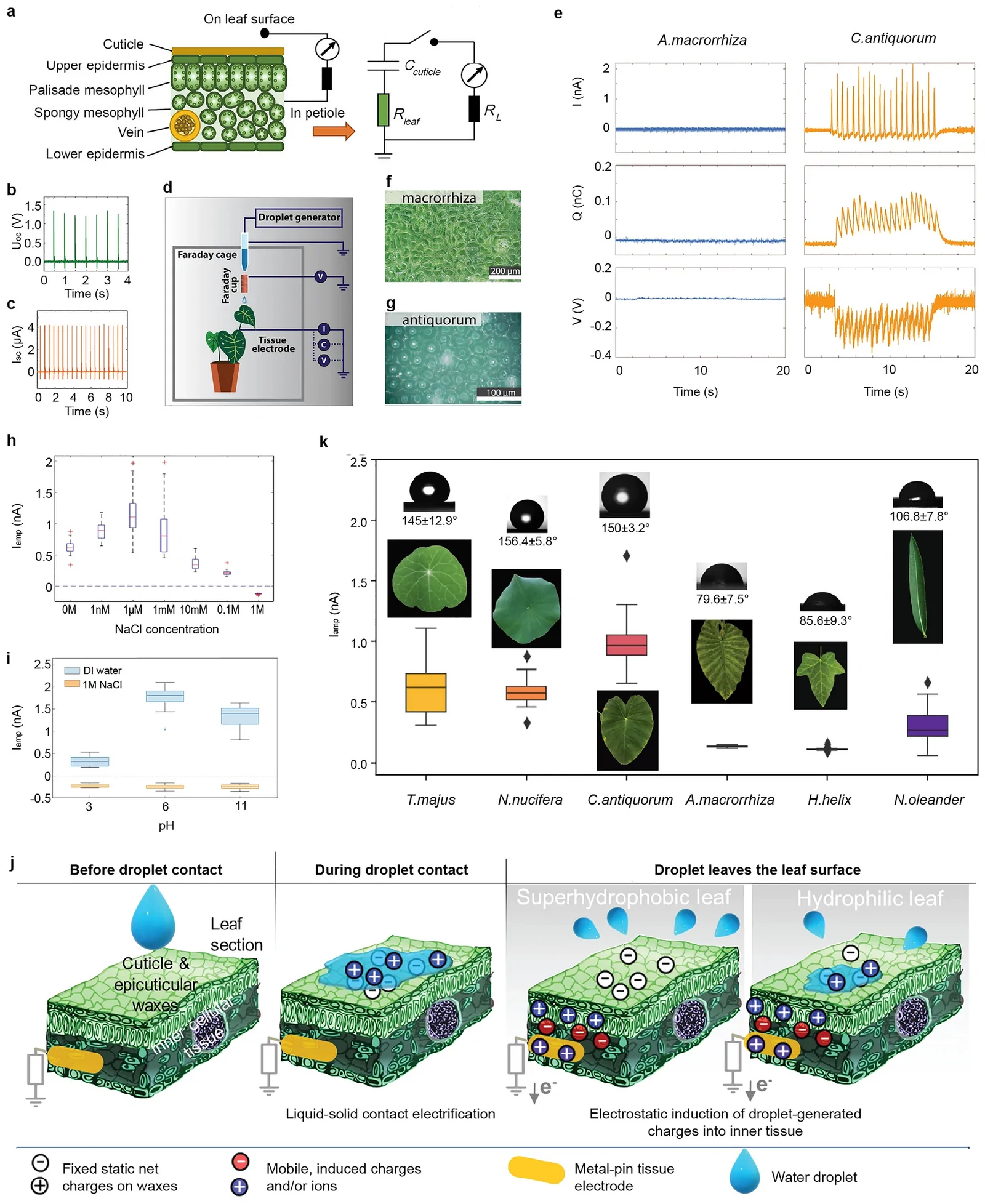
Cellulose-based DEGs. **a** Schematic of the cross section of a leaf connected to an external circuit and its equivalent circuit. **b** Open-circuit voltage and **c** short-circuit current generated by the water droplet impact on a *Mytilaria laosensis* Lec. leaf. **a-c** Reproduced with permission [33]. Copyright 2020, American Chemical Society. **d** Schematic of plant leaf-based DEGs. **e** DEG output characteristics using two different leaves. **f, g** Digital microscopy of the living leaf showing the surface cellular structure. **h, i** Output current of a *C. antiquorum* leaf-based DEG at different ion concentrations and pH levels. **j** Proposed electricity-generation mechanism of leaf-based DEGs. **k** Output current of different plant leaf-based DEGs. Reproduced with permission [209]. Copyright 2022, Springer Nature

Cellulose, the primary structural component of plant leaves, provides a naturally porous and ion-permeable framework that can act as both a biodegradable substrate and an ionic electrode in droplet-based electricity generation. When a water droplet impacts the leaf surface, the cuticular wax layer—a dielectric film covering the cellulose epidermis—forms a transient liquid–solid junction, whereas the underlying cellulose-rich mesophyll acts as an ionic conductor connected to the vascular network. This configuration yields a self-contained circuit in which the droplet serves as the top electrode and dynamic switch. Upon contact, the circuit transitions from an “off” to an “on” state, releasing stored surface charges through the cellulose-based conductive tissue; as the droplet retracts, the current reverses due to charge redistribution across the interfacial capacitance (Fig. 12a). In situ measurements on living leaves have reported open-circuit voltages of ~ 1.3 V, short-circuit currents of ~ 4 μA, and power outputs approaching 1 μW at a 200 kΩ load resistance (Fig. 12b, c) [33]. Collectively, these findings confirm that the cellulosic plant matrix provides both mechanical compliance and an intrinsic ionic pathway for charge transport, establishing a fully biodegradable droplet energy harvester without external electrodes or synthetic polymers.

A subsequent mechanistic investigation correlated the interfacial electrical signal with the chemical composition and micro-/nanostructure of the wax layer overlying the cellulose substrate (Fig. 12d–k) [209] Superhydrophobic leaves with well-preserved wax crystals exhibited significantly stronger voltage peaks than dewaxed or molten-wax surfaces, indicating that both the surface chemistry and hierarchical morphology govern the charge-separation efficiency (Fig. 12e–g). Because the wax layer is directly anchored to the cellulosic epidermal wall, its microstructure controls droplet adhesion, contact-line mobility, and consequently dA/dt. When the wax is removed, the exposed cellulose fibers exhibit higher surface energy and hydrophilicity, leading to persistent water films that electrically short-circuit the device and suppress output. Electrolyte conditions further modulate the response: low ionic strengths (< 1 mM) slightly enhance charge transfer, whereas higher concentrations screen interfacial charges and can even induce polarity reversal (Fig. 12h). Variations in pH and pre-charged droplets also affect signal amplitude, indicating that cellulose-supported interfacial chemistry is strongly coupled to environmental ion dynamics (Fig. 12i).

More recently, artificial cellulose-based DEGs have begun to move beyond natural leaf systems by introducing hydrophobic engineering, dielectric enhancement, and rational electrode design into cellulose substrates. A representative example is hydrophobic sisal cellulose paper, in which cellulose paper was modified by physical adsorption of hydrophobic polymers or by chemical grafting of MMA to improve wettability and dielectric properties, and then integrated into interdigital droplet-harvesting devices [210]. The enhanced hydrophobicity suppressed water retention on the cellulose surface and promoted more effective charge separation during droplet impact and motion, while the increased dielectric constant further amplified the electrical output. As a result, the optimized device delivered open-circuit voltages up to 16 V and a peak power density of 8.2 mW m^−2^, and also exhibited a nearly linear dependence of output on droplet velocity, enabling raindrop sensing in addition to energy harvesting. These results indicate that, for cellulose-based artificial DEGs, surface hydrophobization is not merely a passive protection strategy against wetting, but a key interfacial design parameter that determines droplet detachment behavior, charge retention, and output stability.

At a smaller structural scale, CNCs have also emerged as promising DEG building blocks because their high crystallinity, ordered molecular arrangement, and film-forming capability provide a stable dielectric matrix for droplet-triggered interfacial electrification. In a recent study, a Nafion-functionalized CNC film coated with a paraffin hydrophobic layer was assembled into a DEG with top and bottom electrodes, where droplet contact, spreading, and retraction induced transient charge redistribution and reversible current generation at the liquid–solid interface [211]. The Nafion coating introduced fluorinated and sulfonic groups that strengthened interfacial electrostatic interactions, while the cellulose nanocrystal scaffold preserved structural integrity and enabled durable operation. The resulting device produced voltages up to ~ 90 V, currents of ~ 5 μA, and a peak power density of ~ 310 mW m^−2^, together with stable operation over 7 days.

Besides cellulose nanocrystals, cellulose paper combined with biomass-derived lignin has provided another route toward sustainable artificial DEGs. By esterifying lignin with palmitoyl chloride and spray-coating it onto cellulose paper, a petal-inspired superhydrophobic interface with multiscale roughness was constructed, which markedly improved droplet rolling, rebound, and interfacial drainage [212]. Compared with untreated cellulose paper and unmodified lignin-coated paper, the petal-like lignin/cellulose surface showed much higher water repellency (contact angle up to 163°) and more stable droplet-triggered electrical output, yielding voltages of 4.7 V and currents of 470.1 nA under simulated raindrop impact. Importantly, this work shows that, in artificial cellulose-based DEGs, surface micro/nanostructure and wettability regulation are inseparable from electrical performance: by minimizing water-film formation and maintaining rapid contact–separation dynamics, the biomimetic cellulose interface sustains more efficient charge generation under continuous rainfall.

To better position DEGs within interfacial energy-harvesting systems, a brief comparison with TENGs is instructive. DEGs mainly rely on dynamic interfacial charge redistribution during droplet impact, spreading, sliding, or detachment, often accompanied by electrical double-layer reconstruction and capacitive effects at the liquid–solid interface. In contrast, TENGs are primarily based on contact electrification and electrostatic induction during contact–separation processes. Despite these differences, both systems depend strongly on interfacial properties such as surface chemistry and wettability. However, DEGs are generally more relevant to liquid-phase or humid environments, whereas TENGs are more commonly discussed in mechanically driven contact–separation systems in dry environment.

Collectively, these studies establish a coherent model of cellulose-based DEGs as bio-derived capacitive systems in which (i) the cellulose framework provides the ionic conduction network and structural elasticity, (ii) the wax-functionalized surface atop the cellulose regulates wettability and charge generation, and (iii) their coupled dynamics dictate both the magnitude and sign of the transient current. This mechanistic understanding offers a blueprint for designing artificial cellulose platforms—for example, nanocellulose films or aerogels with engineered hydrophobic microtextures—that emulate the natural leaf’s ability to harvest energy from raindrops while remaining biodegradable, flexible, and environmentally compatible. By translating the intrinsic cellulose–wax–water interplay to engineered materials, next-generation sustainable DEGs could convert ubiquitous droplet motion into electrical power under ambient conditions, further advancing the cellulose-enabled hydrovoltaic paradigm.

Compared with the above three types of HEGs, cellulose-based materials have been less explored in DEGs. This is partly because DEG performance strongly depends on droplet dynamics, including impact, spreading, sliding, and detachment, which are closely related to surface wettability and adhesion. Efficient DEG systems often require hydrophobic or low-adhesion surfaces to facilitate rapid droplet motion and dynamic interfacial charge redistribution. In contrast, cellulose is intrinsically hydrophilic and is more commonly used in systems relying on continuous water transport and ion migration in nanochannels, such as evaporation-driven generators. Therefore, current cellulose-related DEG studies are more frequently based on natural leaf structures or bioinspired surface modification rather than purely artificial cellulose nanochannel systems. This also suggests that surface wettability engineering and bioinspired structural design may be important directions for developing cellulose-based DEGs in the future.

## Applications of Cellulose-enabled HEG Devices

### Power Sources

**Fig. 13:**
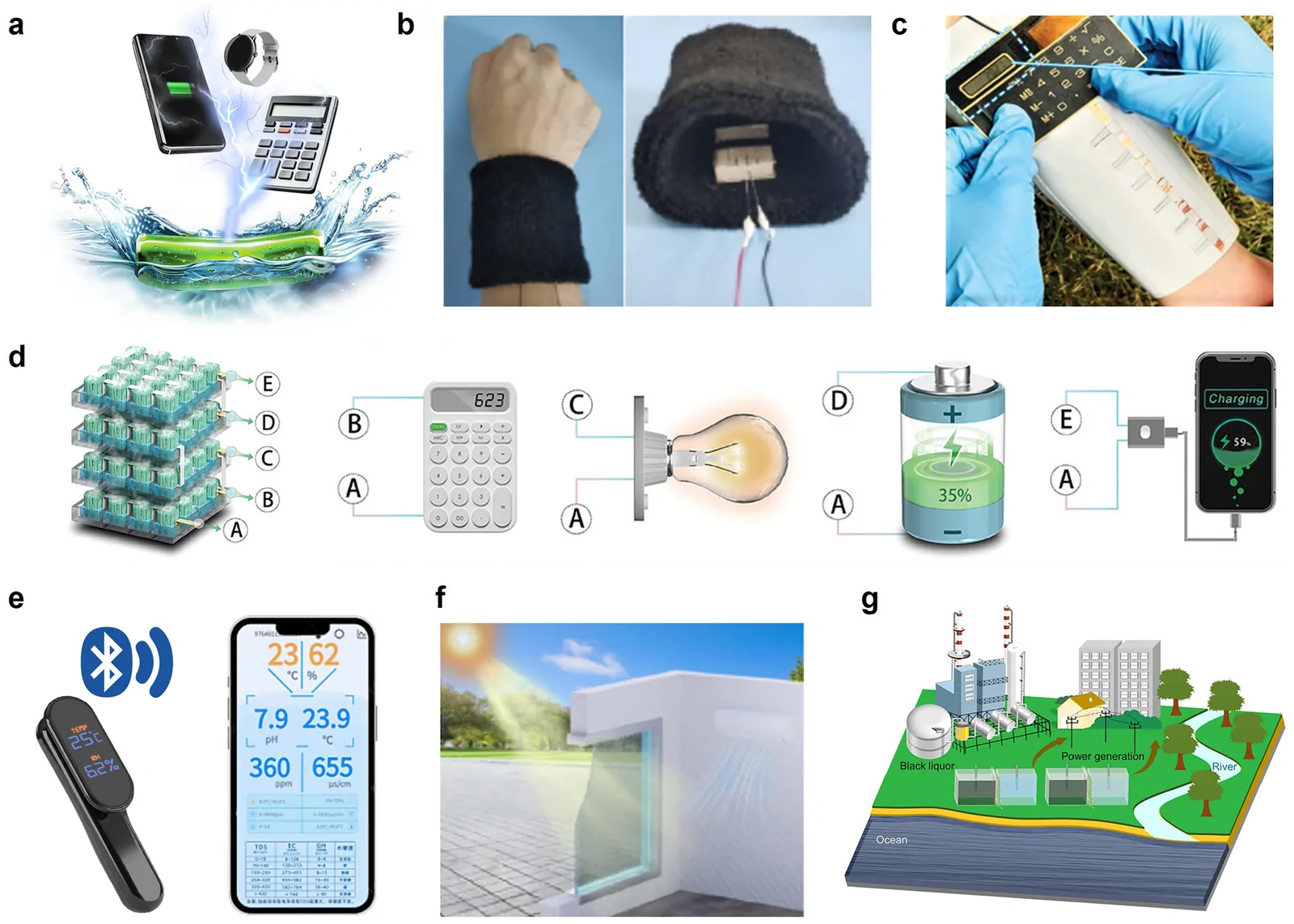
Applications of cellulose-based HEGs as power sources. **a** Schematic of a cellulose-HEG-based power source. **b** Arrayed cellulose-HEG modules charging a battery. Reproduced with permission [150]. Copyright 2024, American Chemical Society. **c** Sweat-enabled cellulose-HEG directly powering a digital calculator on a flexible substrate. Reproduced with permission [38]. Copyright 2022, Wiley–VCH. **d** Schematic of an integrated system of cellulose-HEG arrays for scaled electricity generation. Reproduced with permission [156]. Copyright 2024, Wiley–VCH. **e** Cellulose-HEG integrated with a sensor module for wireless environmental data transmission to a smartphone. Reproduced with permission [143]. Copyright 2023, Elsevier. **f** Smart window systems powered by cellulose-HEGs using indoor–outdoor humidity gradients. Reproduced with permission [108]. Copyright 2024, American Chemical Society. **g** Conceptual diagram of cellulose-based HEGs integrated into large-scale biomass recycling and power-generation systems. Reproduced with permission [178]. Copyright 2023, Elsevier

One of the most direct applications of cellulose-HEG devices is as sustainable power sources, generating electrical energy without external power input (Fig. 13a). In early development, their relatively low output limited demonstrations to low-power devices such as LED lights and e-paper displays. However, recent advances in materials engineering, device integration, and surface functionalization have substantially increased both the voltage and current outputs [42].

For example, three capacitors connected in series were able to power an electronic calculator, underscoring the practical feasibility of textile-based cellulose-HEG devices for low-power electronics. Zhang et al. demonstrated a wearable, moisture-powered wristband composed of arrayed cellulose-EEG modules that continuously harvested ambient humidity to charge small batteries (Fig. 13b) [150]. The cellulose-EEG array produced a steady DC output in real time from either atmospheric moisture or perspiration, enabling the charging of the coin-cell battery of the wristband. The device also remained mechanically robust under bending and movement, highlighting the flexibility and wearability of the cellulose-derived generators. Similarly, flexible and biocompatible cellulose-HEG devices have directly powered electronic calculators using body moisture (e.g., sweat), demonstrating their potential for wearable energy systems (Fig. 13c) [38]. Fu et al. further demonstrated that 36 RC/40 wt% acidified CNT fibers, woven into flexible fabrics and connected in series or parallel, could charge a 1000 μF capacitor to 0.4 V within just 10 min.

Stacking cellulose-HEG devices into multilayer assemblies is an effective strategy for achieving output levels matched to application-specific voltage and power requirements. By connecting multiple cellulose-based generator layers in series and/or parallel, the total V_oc_ can be increased from the sub-volt range of a single unit to several volts or more. Du et al. showed that a stacked cellulose-HEG array can directly power common devices when appropriately configured (Fig. 13d) [156]. In their system, varying the number of stacked cellulose-HEG films tuned the available voltage and enabled operation of a digital calculator, illumination of an LED bulb, charging of a battery module, and intermittent charging of a smartphone.

Moreover, cellulose-based HEG devices have been successfully integrated into environmental sensing platforms, enabling wireless transmission of humidity, temperature, and pH data to smartphones without external batteries. For example, Li et al. developed a cotton-based HEG device coupled with a sensing module to enable the real-time monitoring of water-quality parameters, including pH, hardness, and conductivity (Fig. 13e) [143]. Three HEG units connected in series generated sufficient power to operate both the sensors and wireless communication module, thereby eliminating the need for an external battery. The measured data were transmitted directly to a smartphone, demonstrating the potential of cellulose-based HEG devices as compact and autonomous power sources for wireless environmental sensing.

Yao et al. proposed cellulose-based HEG devices for smart window applications (Fig. 13f) [108] and developed a cellulose-based ionogel film comprising CNF, PIL, and IL, wherein the cellulose component provided high optical transparency, strong moisture permeability, and robust mechanical strength. These attributes enable the ionogel to be applied in building-integrated energy systems, such as smart windows that harvest electricity from indoor–outdoor humidity gradients. The combination of optical clarity, breathability, and structural integrity supported efficient energy harvesting from ambient humidity differences while maintaining suitability for practical window applications.

Wang et al. proposed a large-scale osmotic energy-harvesting strategy that integrated cellulose-based HEG devices into biomass-recycling systems (e.g., pulp mills), where cellulose waste streams and environmental moisture could be leveraged simultaneously for energy recovery (Fig. 13g) [178]. They developed a BC-based DN hydrogel with interconnected nanochannels to efficiently convert salinity gradients—derived from sources such as black liquor and seawater—into electricity. Given the high hydrophilicity, tunable chemistry, and scalability of BC, this approach offers a low cost and sustainable route for energy recovery from industrial effluents in coastal and riverside facilities.

Collectively, these applications highlight the increasing versatility and scalability of cellulose-based HEG devices, positioning them not only as eco-friendly power sources for miniaturized electronics but also as potential contributors to sustainable urban and industrial energy networks. This breadth of use underscores both the current practicality and longer-term potential of cellulose-based HEG in multifunctional energy systems.

### Self-Powered Health Care Monitoring Sensors

Self-powered cellulose-based HEG shows significant potential in healthcare applications, particularly for the continuous, real-time monitoring of physiological signals. Conventional healthcare sensors for tracking breathing or movement typically rely on external power supplies, which limit their long-term use in wearable and portable systems [213]. Conversely, cellulose-based HEG devices can harvest ambient moisture or mechanical deformation to generate an electrical output, enabling autonomous, battery-free signal acquisition. In addition, cellulose is a natural, biocompatible material, supporting its use in healthcare monitoring devices.

**Fig. 14:**
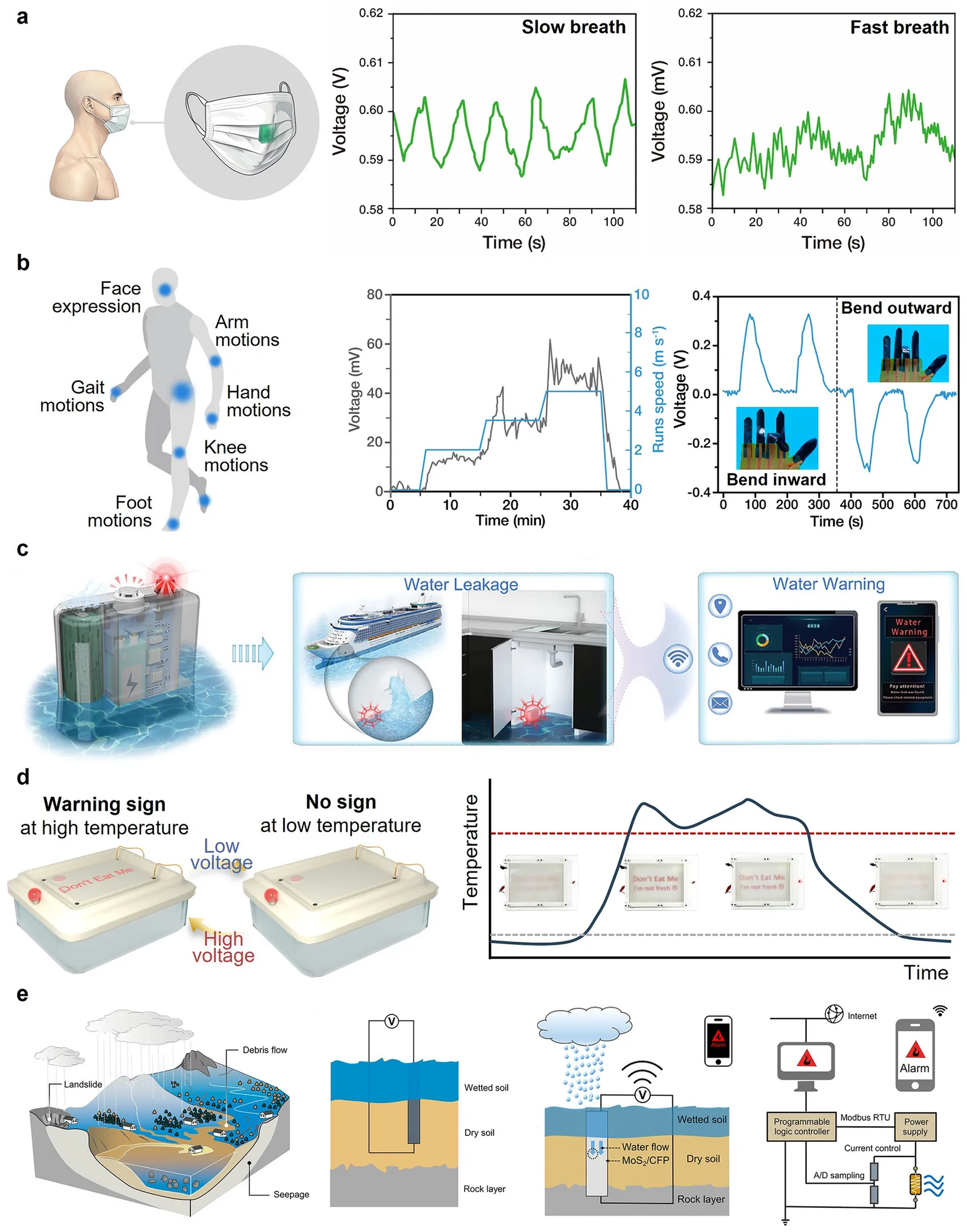
Applications of cellulose-based HEGs in health care monitoring sensors and warning systems. **a** Concept of a self-powered breathing sensor employed in a cellulose-based HEG. Reproduced with permission [96]. Copyright 2022, Elsevier. **b** Concept of a self-powered water-stimuli-responsive physical sensor employed in a cellulose-based HEG. Reproduced with permission [32]. Copyright 2019, Wiley–VCH. Reproduced with permission [103]. Copyright 2021, American Chemical Society. **c** Schematic of a cellulose-based HEG–assembled sensor system for water leakage detection and warning. Reproduced with permission [156]. Copyright 2024, Wiley–VCH. **d** Self-powered cellulose-based HEG smart packaging for food freshness monitoring. Reproduced with permission [40]. Copyright 2024, Royal Society of Chemistry. **e** Early warning and forecasting of geological seepage using a cellulose-based HEG. Reproduced with permission [148]. Copyright 2023, American Chemical Society

Breath monitoring is among the most promising and widely demonstrated applications of cellulose-based HEG (Fig. 14a) [96]. Respiration is a key vital sign; monitoring the breathing rate and depth can support health assessment in elderly and infirm patients, facilitate sleep apnea screening, and indicate oxygen demand and exercise intensity during physical activity. Most energy-harvesting, self-powered devices for monitoring breathing operate by sensing the voltage changes induced by fluctuations in the ambient humidity. In this context, cellulose-based MEGs integrated into wearable masks or patches can detect humidity variations during inhalation and exhalation, generating characteristic voltage profiles associated with slow, normal, and rapid breathing cycles. These periodic signals enable the noninvasive tracking of the respiratory rate and intensity, supporting applications such as sleep apnea detection, physical-activity monitoring, and the early identification of respiratory disorders. Such systems have been implemented using cellulose-based membranes embedded within conventional face masks, enabling real-time monitoring without compromising user comfort or requiring battery replacement.

Another important healthcare application of cellulose-based HEG devices is self-powered, water-stimuli-responsive physical sensing (Fig. 14b) [109]. In wearable healthcare systems, these sensors can support personal health management by converting moisture-related physical changes into electrical signals that reflect activity intensity, posture, or rehabilitation progress. Unlike conventional motion sensors that rely solely on mechanical deformation, cellulose-based HEG devices respond sensitively to water-related stimuli, including perspiration, local humidity, and vapor flux generated during movement. When attached to the skin or placed near joints, changes in the sweat rate and surface moisture during bending or stretching modulate water–ion transport at the cellulose interface, yielding distinct voltage outputs. For example, a finger-mounted cellulose-based HEG device produces alternating voltage signals during flexion and extension, consistent with dynamic moisture redistribution at the surface. Similarly, increased perspiration and vapor flow during fast running enhance ionic diffusion within the device, resulting in a higher open-circuit voltage that correlates with exertion level. Collectively, these features indicate that cellulose-based HEG devices can function as self-powered, water-stimuli-responsive physical sensors that convert physiological moisture fluctuations and mechanical movement into quantifiable electrical outputs. Their inherent flexibility, breathability, and biocompatibility further support the stable and comfortable operation of long-term wearable monitoring device.

### Self-Powered Warning Systems

Beyond basic sensing, cellulose-based HEG devices can enable long-term condition monitoring and abnormality detection. Xu et al. proposed a cellulose-based HEG leak-detection unit for the continuous monitoring of stray moisture (Fig. 14c) [156]. A hygroscopic cellulose element is positioned near plumbing or other water fixtures; when leakage occurs, water is wicked into the porous network, initiating capillary flow. This flow drives ion migration and establishes an electrical potential that can be harvested to power a small radio or indicator. In practice, the device remains inactive under dry conditions but transmits a wireless alert (e.g., via a low-power transmitter or a blinking LED) immediately upon moisture detection. Because the system is entirely powered by the harvested leakage energy, it eliminates the need for batteries. This autonomous operation enables real-time, maintenance-free surveillance of kitchens, bathrooms, and industrial piping. For example, cellulose-based MEGs have been reported to generate sufficient voltage to power sensors and LEDs, demonstrating the feasibility of fully self-powered leak alarms.

Park et al. demonstrated a self-powered smart packaging label that reports food freshness through its voltage output (Fig. 14d) [40]. In this system, a warning label bearing the phrase “Do not Eat” is printed and covered with a polymer-dispersed liquid crystal (PDLC) film. Under normal conditions, the PDLC remains opaque, concealing the label. However, upon exposure to elevated temperatures, the cellulose-based MEG generates sufficient output voltage to trigger a phase transition in the PDLC, rendering it transparent and revealing the warning message. This design allows consumers to instantly determine whether a package has been subjected to undesirable thermal exposure during storage or transport. To enable continuous temperature monitoring and visual alerts, the system incorporates a light sensor, an LED, and an Arduino-based control unit. If high-temperature exposure persists beyond a predetermined duration, the PDLC remains transparent and triggers LED illumination, providing a clear indication of the temperature breach. This self-powered packaging an approach is particularly well suited to cold-chain logistics, where access to external power supply is limited. It offers a sustainable strategy for the real-time quality monitoring of perishable products, including fresh foods and pharmaceuticals. Furthermore, the cellulose-based MEG units are enzymatically degradable and recyclable, providing an environmentally friendly alternative to conventional battery-powered devices and reducing electronic-waste concerns.

Ling et al. developed a self-powered water-seepage early warning system based on a cellulose-HEG (Fig. 14e) [148]. In this design, the cellulose sensor is placed in soil or a subsurface tunnel to absorb groundwater seepage. Under dry conditions, the ionic flow, and thus the output voltage, is minimal, whereas heavy rainfall or a rise in the water table markedly increases the moisture and ion content. When the generated voltage or current exceeds a preset threshold, the module automatically activates and issues a warning (e.g., by powering a radio beacon connected to a monitoring network). This concept was implemented in a “soil-powered” landslide sensor, in which soil ions generated electricity that activated the system only when moisture surpassed a critical level. Because the cellulose-based HEG device continuously harvests energy from ambient soil moisture, it can remain dormant until needed and operate for many years without batteries. Such self-sustaining seepage sensors can be deployed on hillsides or behind levees to provide long-term, real-time early warnings of floods and landslides.

## Conclusion, Challenges, and Outlook

### Conclusion

In this review, we systematically examined the role of cellulose across three main HEG categories—MEGs, EEGs, and OEGs—and highlighted its multifaceted contributions to charge separation, ion transport, and water management. Through an in-depth analysis of chemical modification strategies, composite formation, structural design, and device architecture, we illustrated the rapid progress and broad potential of cellulose-based HEG devices as power sources for electronic devices and self-powered sensing systems. To date, cellulose-based HEG devices in the MEG, EEG, and OEG categories have received significant attention. By contrast, only a limited number of DEG-type cellulose-based HEG devices have been reported, leaving considerable scope for both fundamental investigations and technological breakthroughs.

**Fig. 15:**
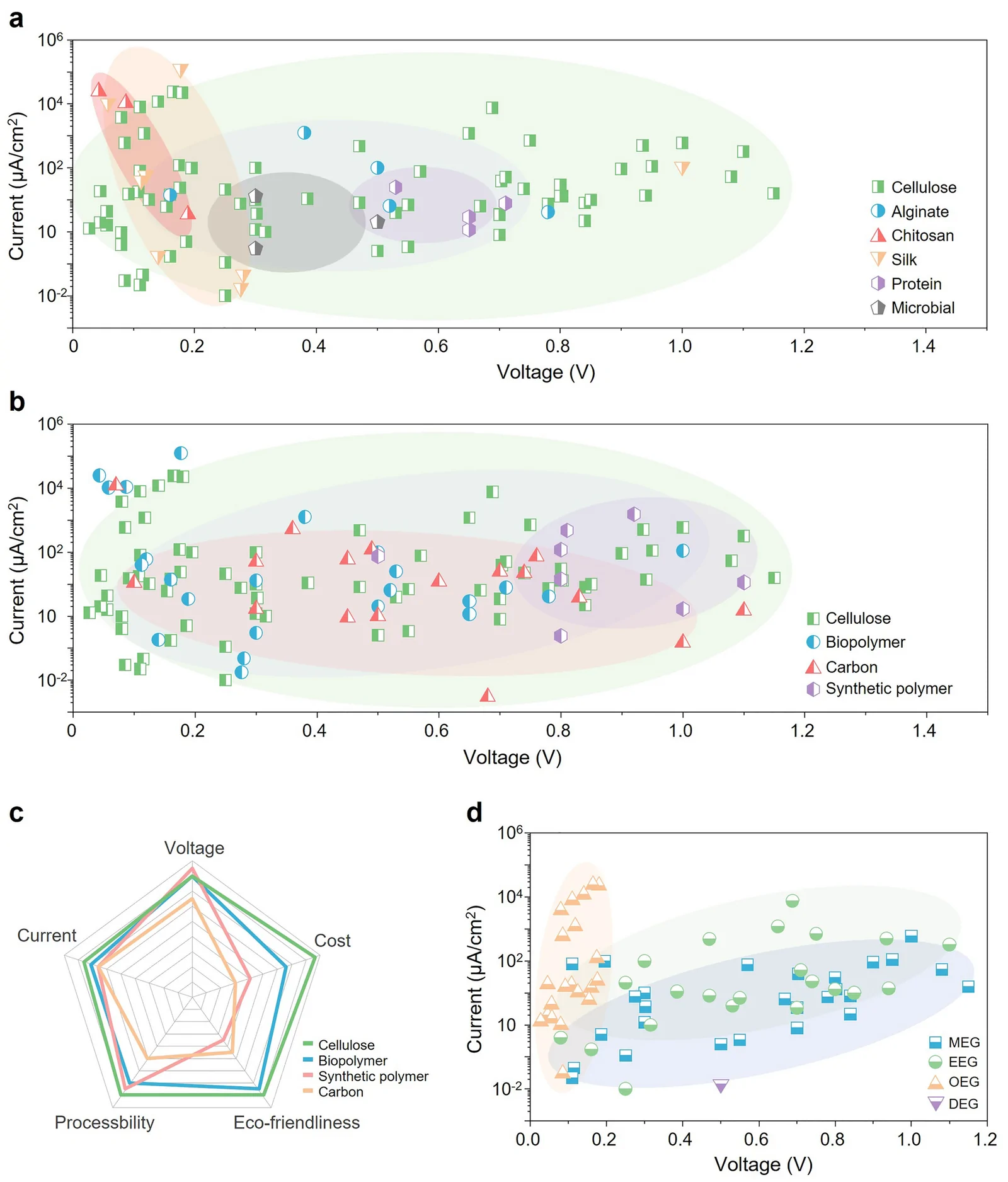
Comparative analysis of cellulose-based HEGs in terms of material origin, classification, and device type. **a** Electrical performance distribution across HEGs based on different biopolymer materials. **b** Comparison of electrical performance of HEGs across broad material classes. **c** Radar plot benchmarking representative HEG materials based on voltage output, current density, processability, eco-friendliness, and cost. **d** Electrical performance comparison of MEG-, EEG-, OEG-, and DEG-type cellulose-based HEGs

As shown in Fig. 15, cellulose-based HEG devices are comprehensively compared based on their material composition, classification, and device type. Among biopolymers (alginate, chitosan, silk, protein, and microbial), cellulose-based HEG devices exhibit the broadest and most balanced voltage and current ranges. They have also been the subject of the largest number of published papers, underscoring both their versatility and maturity in the field (Fig. 15a). A detailed analysis of the performance metrics for each biopolymer-based HEG device revealed distinct operating ranges. Cellulose-based HEG devices span wide ranges in both voltage (up to 1.15 V) and current density (up to 24,100 μA cm^−2^), supporting their broad applicability. Alginate-based HEG devices show mid-range performances in both voltage (0.16–0.78 V) and current density (4.13–1,260 μA cm^−2^). Chitosan- and silk-based HEG devices generally exhibit lower voltage outputs (0.043–0.189 and 0.058–1.0 V, respectively) but can deliver exceptionally high current densities. Notably, silk-based HEG devices reach the highest reported current density (124,000 μA cm^−2^), while chitosan-based HEG devices also achieve high values (up to 25,000 μA cm^−2^). By comparison, protein- and microbial-based HEG devices tend to exhibit lower voltage and current outputs and have been the subjects of relatively few studies, suggesting that these subfields remain at an early stage of development.

The analysis was further extended to broader material classes, including cellulose, other biopolymers, carbon-based materials, and synthetic polymers (Fig. 15b). Cellulose-based HEG devices maintain a balanced performance profile and show notably higher voltage outputs compared with other biopolymers. Although biopolymer- and carbon-based HEG devices can reach high peak current densities (up to 124,000 μA cm^−2^ for biopolymers and 12,000 μA cm^−2^ for carbon), their voltage ranges are slightly narrower. In contrast, synthetic polymer-based HEG devices exhibit a relatively higher voltage range (0.5–1.25 V) but a more limited current output (0.24–1,550 μA cm^−2^), indicating a distinct performance trade-off. Collectively, these comparisons emphasize that cellulose is a uniquely versatile platform that combines broad, balanced voltage and current outputs, positioning it as a highly versatile and promising material for HEG applications.

A multicriteria assessment (Fig. 15c) further supported the strong overall positioning of cellulose-based HEG devices relative to devices based on natural polymers, carbon materials, and synthetic polymers. In addition to the electrical performance (voltage and current density), this evaluation considered attributes central to sustainable and scalable deployment, including processability, eco-friendliness, and cost-effectiveness. Recent advances in structural optimization and interfacial engineering have substantially narrowed the performance gaps with other material systems, and cellulose-based HEG devices no longer exhibit a clear disadvantage in power-related metrics. Furthermore, cellulose enables aqueous, low-temperature fabrication and is compatible with large-area, roll-to-roll manufacturing in various formats such as films, fibers, aerogels, and porous membranes. Abundant renewable feedstocks and mature supply chains further enhance its cost-effectiveness and eco-friendliness. Nevertheless, several intrinsic limitations remain when benchmarking cellulose against alternative materials, including its reduced durability under harsh or persistently humid environments, the need for precise and reproducible control of the nanocellulose microstructure, and the sensitivity of capillarity-driven ion transport to the porosity and water uptake. These factors can limit its long-term stability and reproducibility compared with some synthetic polymers or carbon-based materials and, therefore, remain important targets for ongoing material and device engineering.

Device-type comparisons (Fig. 15d) indicated that OEGs typically deliver higher current densities but lower voltages, whereas MEGs and EEGs generally produce higher voltages and broader current–density ranges. Within these categories, EEG-type HEG devices show slightly superior overall performance than MEG-type devices. The data for DEGs remains too limited to establish meaningful performance trends.

### Challenges

Despite substantial progress in cellulose-enabled HEGs, several key challenges must be addressed to achieve robust, scalable, and practically deployable performance.

#### Fundamental Charge-transport Mechanisms and Theoretical Modeling Gaps

Despite substantial progress in cellulose-enabled HEGs, several key challenges must be addressed to achieve robust, scalable, and practically deployable performance. The hierarchical structure of cellulose—comprising crystalline domains, amorphous regions, and abundant hydroxyl groups—governs water adsorption, ion transport, and surface charge regulation. However, a primary theoretical challenge lies in the inadequacy of classical electric double layer (EDL) and Debye length models for these systems. These frameworks are predicated on ideal, rigid, and uniformly charged solid–liquid interfaces; however, cellulose-based materials exhibit non-rigid, dynamic swelling behavior and an evolving hierarchical porous structure during hydration. This leads to a dynamic reconstruction of the EDL that deviates significantly from classical assumptions, requiring new “correction schemes” and boundary definitions that account for the intrinsic properties of swelling cellulose.

Furthermore, the fundamental mechanism of internal charge transport, specifically the Grotthuss proton hopping mechanism, remains insufficiently integrated with the core characteristics of cellulose. Proton transport efficiency is intrinsically tied to the continuity of the hydrogen-bond network, which is directly modulated by the crystallinity, chemical modification, and moisture-induced swelling of the cellulose matrix. Currently, a robust structure–activity relationship that links the dynamic evolution of this hydrogen-bond network to macroscopic proton transport efficiency has yet to be established. Bridging these gaps will require advanced in situ characterization and multiscale modeling to accurately link molecular-scale proton dynamics and non-ideal EDL behavior to the macroscopic electrical output observed in hierarchical porous systems.

#### Multi-mechanism Coupling and Theoretical Efficiency Limits

In this review, we described 4 types cellulose-enabled HEGs (MEGs, EEGs, OEGs, and DEGs). A major challenge is the inherent coupling of multiple hydrovoltaic mechanisms within porous cellulose. Although HEGs are often categorized as independent modes, these processes rarely occur in isolation. For instance, humidity gradients often induce local evaporation, which simultaneously generates streaming potentials. Systematic studies on the synergistic or antagonistic effects between these coupled drivers are currently lacking. Developing standardized methods to quantitatively decouple these contributions remains a core, unresolved hurdle in the field. Additionally, the theoretical maximum energy-conversion efficiency for each HEG category is not yet well-defined. It is crucial to determine how closely current cellulose-based systems approach their thermodynamic and kinetic limits. Addressing this gap requires sophisticated experimental designs to isolate specific drivers and standardized benchmarking to distinguish their individual contributions. Such clarity is essential for the rational optimization of high-performance hydrovoltaic devices.

#### Scalability and Process Reproducibility

Despite strong laboratory-scale performance, the scalability of cellulose-enabled HEGs remains a major bottleneck. Controlling film thickness, maintaining uniform moisture gradients, and ensuring consistent ionic distribution across large areas are technically challenging. Furthermore, scalable fabrication methods (e.g., roll-to-roll coating and printing) can introduce defects that compromise electrical stability. The absence of standardized manufacturing protocols further limits batch-to-batch reproducibility and hinders industrial translation.

#### Stability under Humid Environments and Intrinsic Limitations of Cellulose

Cellulose is inherently hydrophilic, and while this property underpins hydrovoltaic electricity generation, it also promotes swelling, delamination, structural collapse and mechanical degradation during prolonged exposure to humid conditions. Such instability issues cause performance drift and shorten device lifetime. Specifically, swelling-induced mechanical and electrochemical degradation poses a significant challenge, as it disrupts the fiber network and alters ion-transport pathways. In long-term high-humidity environments, cellulose-based devices are also vulnerable to microbial degradation, mildew, and the hydrolysis or loss of chemically modified functional groups. These are unique bottlenecks that distinguish cellulose-based HEGs from inorganic or synthetic polymer systems. A key challenge lies in balancing moisture sensitivity for energy generation with sufficient structural robustness for continuous operation. Strategies including surface passivation, crosslinking, and hybrid encapsulation are under investigation; however, trade-offs among ion transport, electrical output, and long-term durability remain unresolved.

#### Material and Interfacial Engineering Limitations

Chemical modifications (e.g., carboxylation, sulfonation, and polymer grafting) have been employed to enhance charge-carrier mobility and ion selectivity. However, performance degradation often stems from functional-group loss and ion depletion within the modified matrix. A systematic comparative analysis of the long-term efficacy of various modification strategies is currently lacking. Similarly, incorporating conductive fillers or 2D nanomaterials can enhance output yet often introduces interfacial mismatch and increased internal resistance. Also, these modification strategies often compromise mechanical flexibility or environmental compatibility. For example, in ionic liquid-modified systems, the leaching and leakage of additives during hydration cycles pose significant operational risks. Biotoxicity and poor long-term compatibility with the cellulose network remain core obstacles to industrialization. Co-optimizing bulk transport, stable interfacial charge transfer and environmental sustainability remains a substantial engineering challenge.

#### Standardization of Evaluation and Electrode Effects

Standardized testing protocols are essential to accurately assess the intrinsic performance of cellulose-based HEGs. The use of non-inert metal electrodes (e.g., Al, Zn, and Cu) can induce unintended Faradaic reactions. These electrochemical processes complicate the distinction between true hydrovoltaic output and battery-like contributions. Furthermore, electrode corrosion serves as a core mechanism of performance degradation that must be systematically monitored. Even when inert electrodes (e.g., carbon, liquid metals, Au, and Pt) are used, interfacial contact between cellulose and electrodes also significantly affects charge-collection efficiency. Under mechanical bending or humid conditions, these interfaces face high delamination risks. Future studies should evaluate different electrode-integration strategies and discuss effective mitigation approaches to prevent such failures. Beyond electrode effects, the lack of standardized reporting for device dimensions—specifically membrane area and thickness—frequently leads to inconsistencies in power density benchmarks. Establishing benchmarking metrics is necessary for meaningful cross-study comparisons and rational device optimization. Such standardization will support the rational optimization of devices and provide an in-depth summary of the key engineering challenges in the field.

### Outlook

Cellulose-enabled HEGs constitute a promising platform for sustainable and multifunctional energy harvesting, leveraging biocompatibility, flexibility, and environmental friendliness. Future progress should integrate advances in data science, materials design, and device engineering to accelerate the transition from proof-of-concept demonstrations to practical technologies.

#### AI-assisted Design and Predictive Modeling

Data-driven approaches can substantially accelerate materials discovery and optimization. Machine-learning models trained on structural, chemical, and electrokinetic datasets could predict optimal cellulose architectures and compositions that maximize charge-transfer efficiency. Coupling multiscale simulation with high-throughput experimentation could further enable closed-loop design workflows that rapidly identify performance-governing parameters.

#### Integration into Flexible and Wearable Electronics

The inherent softness, lightweight nature, and biocompatibility of cellulose make HEGs ideal candidates for flexible and skin-conformal electronics. These devices can harvest energy continuously from ambient humidity or perspiration, enabling self-powered biosensors, health monitoring systems, and potentially implantable medical devices. Realizing such applications will require multilayer, mechanically robust architectures that maintain stable power generation under repeated deformation.

#### Hybridization with Complementary Energy-harvesting Systems

Integrating cellulose-enabled HEGs with triboelectric, photovoltaic, or thermoelectric technologies can broaden the operational window and mitigate intermittency. Developing impedance-matched interfaces and adaptive power-management circuits will enable reliable multi-modal harvesting, facilitating continuous operation across diverse environmental conditions.

#### Sustainability Assessment through LCA and TEA Frameworks

To ensure that cellulose-enabled HEGs are truly eco-friendly, comprehensive life cycle assessment (LCA) and techno-economic analysis (TEA) should be incorporated early in development. Open databases and standardized system boundaries would support consistent evaluation, guiding material choices, process design, and commercialization toward genuinely sustainable outcomes.

#### Pathways to Scalable Manufacturing and Commercialization

Bridging laboratory demonstrations to industrial production requires reproducible, low cost, and environmentally benign fabrication. Roll-to-roll coating, inkjet printing, and freeze casting are promising for large-area production, but process uniformity and humidity control must be improved. Incorporating AI-assisted process control and digital quality monitoring could further enhance consistency and throughput at industrial scale.

In summary, cellulose-based HEGs represent a compelling intersection of sustainable materials science, energy conversion, and environmental engineering. Realizing their full potential will require a coordinated effort that couples fundamental mechanistic understanding, innovative material design, system-level integration, and scalable manufacturing. With sustained interdisciplinary collaboration, cellulose-enabled HEGs could emerge as the cornerstones of next-generation low-carbon, self-powered technologies.

## Abbreviations

BC: Bacterial cellulose
CA: Cellulose acetate
CMC: Carboxymethyl cellulose
CNC: Cellulose nanocrystals
CNF: Cellulose nanofibers
CNT: Carbon nanotubes
COF: Covalent organic frameworks
DEG: Droplet energy generation, Droplet energy generator
EDL: Electric double layer
EEG: Evaporation energy generation, Evaporation energy generator
GO: Graphene oxide
HEG: Hydrovoltaic energy generation, Hydrovoltaic energy generator
HPC: Hydroxypropyl cellulose
IL: Ionic liquid
MC: Methyl cellulose
MEG: Moisture energy generation, Moisture energy generator
MOF: Metal–organic framework
OEG: Osmotic energy generation, Osmotic energy generator
PANI: Polyaniline
PEDOTPSS: Poly(3,4-ethylenedioxythiophene): poly(styrene sulfonate)
PSSA: Poly(styrenesulfonic acid)
PVA: Poly(vinyl alcohol)
TEMPO: 2,2,6,6-Tetramethylpiperidinyloxy
TOCNF: TEMPO-oxidized cellulose nanofibers
